# Formamido-Diterpenes from the South China Sea Sponge *Acanthella cavernosa*

**DOI:** 10.3390/md10071445

**Published:** 2012-07-02

**Authors:** Ying Xu, Jun-Hui Lang, Wei-Hua Jiao, Ru-Ping Wang, Ying Peng, Shao-Jiang Song, Bao-Hua Zhang, Hou-Wen Lin

**Affiliations:** 1 Laboratory of Marine Drugs, Department of Pharmacy, Changzheng Hospital, Second Military Medical University, Shanghai 200003, China; Email: xy30490@126.com (Y.X.); zhenshanmei.hui@163.com (J.-H.L.); weihuajiao@hotmail.com (W.-H.J.); wangruping_sy@yahoo.com.cn (R.-P.W.); 2 College of Traditional Chinese Materia Medica, Shenyang Pharmaceutical University, Shenyang 110016, China; Email: yingpeng1999@yahoo.com.cn; 3 Eastern Hepatobiliary Surgical Hospital, Second Military Medical University, Shanghai 200438, China

**Keywords:** *Acanthella cavernosa*, formamido-diterpenes, cytotoxicity, antifungal activity

## Abstract

Seven new formamido-diterpenes, cavernenes A–D (**1**–**4**), kalihinenes E and F (**5**–**6**), and kalihipyran C (**7**), together with five known compounds (**8**–**12**), were isolated from the South China Sea sponge *Acanthella cavernosa*. Structures were established using IR, HRESIMS, 1D and 2D NMR, and single X-ray diffraction techniques. The isolated compounds were assessed for their cytotoxicity against a small panel of human cancer cell lines (HCT-116, A549, HeLa, QGY-7701, and MDA-MB-231) with IC_50_ values in the range of 6–18 μM. In addition, compound **9** showed weak antifungal activity against *Trichophyton rubrum* and *Microsporum gypseum* with MIC values of 8 and 32 μg/mL, respectively, compound **10** displayed weak antifungal activity against fungi *Candida albicans*, *Cryptococcus neoformans*, *T. rubrum*, and *M. gypseum* with MIC values of 8, 8, 4, and 8 μg/mL, respectively.

## 1. Introduction

**Figure 1:**
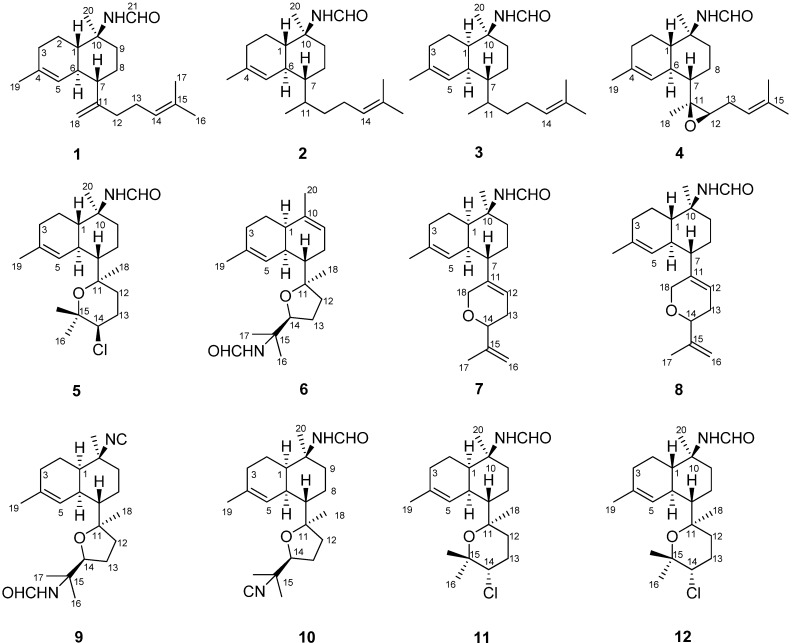
Structures **1**–**12** isolated from *Acanthella cavernosa*.

Marine sponges of the genus *Acanthella* have proven to be a rich source of new diterpenes and sesquiterpenes containing nitrogenous functional groups, including isonitrile (–NC), isothiocyanate (–NCS), isocyanate (–NCO), and formamide (–NHCHO) functionalities, which show various promising biological activities [1,2,3,4,5,6,7]. Diterpenes isolated from this genus have demonstrated cytotoxic [8], anthelmintic [9,10], antimalarial [11], antimicrobial [12], antifungal [1,13,14], and antifouling activities [15,16,17]. In general, these diterpenes can be divided into two types, defined by having a *trans*- or *cis*-decalin moiety. The diterpene precursor has been supposed to be geranylgeraniol, which cyclizes to form a *cis*- or *trans*-biflorane skeleton [6]. In our previous study, we had reported the isolation of eight new diterpenes, kalihinols M–T, together with seven known compounds from the CH_2_Cl_2_ extract of the sponge *A. cavernosa* [18]. As part of our interest on bioactive secondary metabolites from the marine sponges of the genus *Acanthella*, a petroleum ether extract of *A.cavernosa* was investigated and found to be cytotoxic. Further bioassay-guided fractionation of the extract led to the isolation of 12 formamido-diterpenes (**1–12**), including seven new ones (**1–7**) (Figure 1). Details of the isolation, structure elucidation, cytotoxic and antifungal activities of these compounds are described herein.

## 2. Results and Discussion

The sponge *A. cavernosa* was exhaustively extracted with acetone, and after being subjected to extensive column chromatography on silica gel, Sephadex LH-20, ODS, and semipreparative HPLC, compounds **1–12** were obtained. The known compounds, kalihipyran A (**8**) [16], 15-formamido-kalihinene (**9**) [6], 10-formamido-kalihinene (**10**) [6], and kalihinenes X (**11**) and Y (**12**) [15], were identified by comparison of their spectroscopic data with literature values.

In the ^1^H and ^13^C NMR spectra of compounds **1–7**, most signals were doubled in the ratio of ~1.8:1, 2.2:1, 1.1:1, 1.4:1, 2.1:1, 1.9:1, and 1.1:1, respectively. This suggested that 1-7 existed as equilibrium mixtures derived from the s-*trans* and s-*cis* forms of formamide groups, as in the cases of **8–12** [6,15,16], which was supported by IR absorptions (**1**, 1670; **2**, 1681; **3**, 1668; **4**, 1681; **5**, 1682; **6**, 1686; and **7**, 1668 cm^−1^) [16]. The assignments of the formamide groups (NH and CHO) in **1–7** for s-*trans* isomers and s-*cis* isomers were confirmed by HSQC and COSY data (Table 1 and Table 2).

**Table 1:** ^1^H NMR data of compounds **1**–**7** (CDCl_3_, *J* in Hz).

Position	1 *^a^*^, *c*^	2 *^b^*^, *c*^	3 *^a^*^, *c*^	4 *^a^*^, *c*^	5 *^b^*^, *c*^	6 *^a^*^, *c*^	7 *^a^*^, *d*^
1	1.24 m	1.26 m *^e^*	1.65 m *^e^*	1.30 m *^e^*	1.30 m	1.96 m	2.40 m
2a	1.84 m	1.34 m	1.73 m	1.85 m	1.84 m *^e^*	1.87 m	1.48 m *^e^*
2b	1.30 m	1.26 m *^e^*	1.65 m *^e^*	1.30 m *^e^*	1.28 m	1.48 m	1.48 m *^e^*
3a	1.99 m *^e^*	1.97 m *^e^*	1.99 m *^e^*	2.00 m *^e^*	1.93 m *^e^*	2.01 m	2.00 m *^e^*
3b	1.99 m *^e^*	1.97 m *^e^*	1.99 m *^e^*	2.00 m *^e^*	1.93 m *^e^*	1.89 m	2.00 m *^e^*
5	5.24 br s	5.47 br s	5.49 br d (4.0)	5.69 br s	6.37 br s	5.70 br s	5.39 br s
6	2.05 m	1.91 m	2.15 m	2.18 m	2.11 m	2.20 m	2.20 m
7	1.73 m	1.14 m	1.43 m *^e^*	1.17 m	1.58 m	1.62 m	1.78 m *^e^*
8a	1.66 m	1.53 m	1.43 m *^e^*	1.43 m	1.10 m	1.74 m	1.55 m *^e^*
8b	1.50 m	1.26 m	1.26 m	1.57 m	1.75 m	1.94 m	1.55m *^e^*
9a	1.89 m	1.88 m	1.65 m *^e^*	1.90 dt (9.5, 3.5)	1.89 m	5.32 br s	1.55 m *^e^*
9b	1.62 m *^e^*	1.56 m	1.55 m *^e^*	1.60 m	1.62 m		1.55 m *^e^*
11		1.97 m *^e^*	1.75 m				
12a	1.99 m *^e^*	1.26m *^e^*	1.24 m *^e^*	2.62 t (6.5)	1.28 m *^e^*	1.78 m	5.61 m *^e^*
12b	1.99 m *^e^*	1.26 m *^e^*	1.24 m *^e^*		1.28 m *^e^*	1.63 m	
13a	2.13 m	1.97 m *^e^*	1.99 m *^e^*	2.23 m *^e^*	2.27 m	1.83 m	2.18 m
13b	1.99 m *^e^*	1.97 m *^e^*	1.90 m	2.23 m *^e^*	1.97 m	1.56 m	2.13 m
14	5.13 t (5.5)	5.11 t (6.0)	5.08 br s	5.19 t (6.5)	3.93 m	3.75 t (7.0)	3.89 br s
16	1.69 s	1.69 s	1.67 s	1.72 s	1.39 s	1.31 s	4.88 s
							5.01 s
17	1.62 s *^e^*	1.60 s	1.59 s	1.62 s	1.31 s	1.20 s	1.78 s *^e^*
18	4.89 br s	0.78 d (6.9)	0.83 d (6.5)	1.26 s	1.22 s *^e^*	1.18 s	4.16 br s *^e^*
	4.80 br s						4.16 br s *^e^*
19	1.62 s *^e^*	1.67 s	1.65 s *^e^*	1.68 s	1.65 s	1.68 s	1.62 s
20	1.28 s	1.22 s	1.43 s *^e^*	1.27 s	1.22 s *^e^*	1.68 s	1.55 s *^e^*
NH	5.89 d (12.5)	6.10 d (11.8)	5.62 d (12.0)	5.92 d (12.0)	5.66 d (12.5)	5.86 m	5.61 m *^e^*
CHO	8.30 d (12.0)	8.27 d (12.3)	8.24 d (12.5)	8.29 d (12.5)	8.28 d (12.5)	8.19 d (12.5)	8.08 d (2.0)

*^a^* Measured at 500 MHz; *^b^* Measured at 400 MHz; *^c^* Data for major s-*trans* isomer; *^d^* Data for major s-*cis* isomer; *^e^* Overlapped.

**Figure 2:**
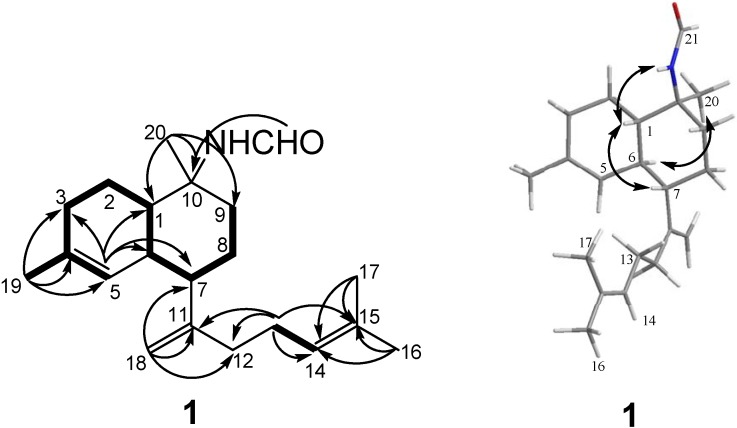
COSY (▬), key HMBC (→), and key NOESY correlations of **1**.

Cavernene A (1) was isolated as a colorless oil. A molecular formula of C_21_H_33_ON was established by the [M + Na]^+^ ion peak at *m/z* 338.2461 in the HRESIMS and supported by NMR data (Table 1 and Table 2), indicating six degrees of unsaturation. The ^1^H NMR spectrum showed the presence of four tertiary methyl [*δ*_H_ 1.28 (3H, s), 1.62 (6H, s), and 1.69 (3H, s)] and four olefinic proton signals resonated at *δ*_H_ 4.80 (1H, br s), 4.89 (1H, br s), 5.13 (1H, t, *J* = 5.5 Hz), and 5.24 (1H, br s). The ^13^C NMR and DEPT spectra of 1 displayed 21 carbon resonances for four methyls, seven methylenes (one olefinic), six methines (two olefinic and one formamido), and four quaternary carbons (three olefinic). The above moieties accounted for four of six degrees of unsaturation, indicating a bicyclic structure for 1. The COSY correlations of H-9/H-8/H-7/H-6/H-1/H-2/H-3/H-5/H-19, together with the HMBC correlations of H_3_-19/C-3, C-4, and C-5, H_3_-20/C-1, C-9, and C-10, H-5/C-1, C-3, C-6, and C-7, and CHO/C-10 indicated the presence of a decalin moiety (Figure 2). The HMBC correlations from the geminal methyls (H_3_-16 and H_3_-17) to C-14 and C-15, from terminal olefinic protons (H_2_-18) to C-7, C-11, and C-12, and from H-13 to C-11, C-12, C-14, and C-15 and COSY correlation between H-13 and H-14 revealed the presence of an isoprenoid unit homolog in 1 and connectivity of the two moieties between C-12 and C-7 through the quaternary carbon C-11.

**Table 2:** ^13^C NMR data (CDCl_3_, *δ* in ppm) of compounds **1**–**7**.

C	1 *^a^*		2 *^b^*		3 *^a^*		4 *^a^*		5 *^b^*		6 *^a^*		7 *^a^*	
	s- *trans*	s- *cis*	s- *trans*	s- *cis*	s- *trans*	s- *cis*	s- *trans*	s- *cis*	s- *trans*	s- *cis*	s- *trans*	s- *cis*	s- *cis*	s- *trans*
1	48.8	45.3	49.0	45.7	45.6	41.1	48.4	45.0	48.8	46.1	40.1	40.1	40.9	45.4
2	22.8	23.1	22.9	23.4	19.1	19.5	22.7	23.1	23.3	23.7	24.6	24.7	18.8	19.1
3	30.96	31.04	30.8	30.9	31.3	31.4	30.8	30.9	30.5	30.6	30.5	30.6	31.1	31.2
4	134.4	134.1	134.9	134.6	134.3	134.2	134.8	134.3	132.4	131.9	131.1	130.8	134.5	134.4
5	123.3	123.8	121.8	122.3	124.3	124.0	122.3	122.7	125.7	126.2	127.1	127.3	123.9	124.2
6	39.5	39.4	38.4	38.2	35.2	34.8	38.6	38.4	39.5	39.2	36.5	36.5	37.0	36.5
7	50.3	50.3	44.6	44.4	42.3	42.0	45.2	45.2	51.2	50.4	44.9	45.2	43.3	43.6
8	29.7	29.9	20.9	20.9	20.1	19.9	25.7	25.8	24.5	24.7	29.48	29.51	28.4	28.4
9	42.0	37.6	41.8	37.4	33.0	33.7	41.7	37.3	42.1	37.8	120.2	120.5	33.5	32.8
10	55.5	57.2	55.6	57.3	55.5	57.1	55.3	56.8	55.4	56.9	136.9	136.8	56.8	55.4
11	151.3	151.9	30.8	30.8	31.3	31.1	62.33	62.7	76.9	77.2	86.4	86.3	139.4	139.0
12	34.2	34.2	35.65	35.69	35.7	35.8	62.27	62.33	31.0	31.7	37.2	37.1	118.6	118.9
13	26.3	26.3	26.2	26.2	26.2	26.3	27.0	27.0	25.9	26.1	25.6	25.8	29.6	29.6
14	124.2	124.3	124.6	124.8	124.7	124.9	119.3	119.5	64.2	64.5	84.2	84.5	77.2	76.8
15	131.7	131.6	131.3	131.1	131.2	131.1	134.2	134.1	74.2	74.4	54.5	55.9	145.4	145.3
16	25.7	25.7	25.7	25.7	25.7	25.7	25.7	25.7	29.7	29.7	27.4	25.1	110.5	110.6
17	17.8	17.8	17.7	17.7	17.7	17.7	18.0	18.0	28.9	28.4	23.8	21.7	18.9	18.9
18	110.0	109.6	13.3	13.3	13.3	13.3	18.5	18.5	21.9	21.2	20.3	21.1	67.63	67.58
19	23.3	23.4	23.6	23.7	23.5	23.4	23.55	23.60	23.8	23.8	23.92	23.87	23.3	23.4
20	19.0	18.9	18.8	18.7	27.2	23.7	18.9	18.7	19.0	18.5	21.53	21.47	23.6	29.7
21	162.8	160.4	163.1	160.5	162.8	160.0	162.8	160.4	162.7	160.4	163.1	160.9	160.1	162.8

*^a^* Measured at 125 MHz; *^b^* Measured at 100 MHz.

The relative configuration of 1 was established on the basis of NOESY data (Figure 2). The NOESY correlations of H-1/H-7 and NH/H-1 indicated that these protons were on the same face of the decalin ring and arbitrarily assigned β-orientations. The α-orientation of H-6 was determined by the NOESY correlation between H_3_-20 and H-6. In addition, the carbon resonances at *δ*_C_ 48.8 (C-1), 39.5 (C-6), 42.0 (C-9), and 19.0 (C-20) in 1 further confirmed the *trans* fusion of the decalin ring [6,8,15]. Cavernene A (**1**) can be envisaged as a decomposable intermediate product of isocyanobifloradiene epoxides in the plausible biogenetic pathway of kalihiprans [3,19].

Cavernene B (**2**) was obtained as a colorless oil. Its molecular formula of C_21_H_35_ON was deduced from the HRESIMS (*m/z* 340.2617 [M + Na]^+^) combined with its NMR data (Table 1 and Table 2), indicating five degrees of unsaturation. The ^1^H and ^13^C NMR spectra of 1 and 2 were comparable except for a marked difference in the isoprenoid unit. Signals for a C-11–C-18 double bond were absent in **2** and replaced by saturated carbons resonated at *δ*_C_ 30.8 and 13.3, respectively. The HMBC correlations of H_3_-18/C-7, C-11, and C-12 and the COSY correlations of H_3_-18/H-11/H-12 and H-11/H-7 supported the assignment of convernene B as **2**. The relative configuration of the decalin ring in **2** was the same as in **1**, with the observation of the NOESY correlations of NH/H-1, H_3_-20/H-6, NH/H-9b, H-7/H-9b, and H_3_-20/H-9a (Supporting Information). The relative configuration at C-11, however, could not be conclusively determined due to conformational flexibility between C-7 and C-11.

Cavernene C (**3**), obtained as white needles, showed the same molecular formula of C_21_H_35_ON as **2** determined by pseudomolecular [M + Na]^+^ ion peak at *m/z* 340.2614 in HRESIMS. Its carbon skeleton was readily assignable as the same as **2** by HSQC, HMBC, and COSY spectra. In particular, the carbon resonances of the isoprenoid unit in **3** were almost superimposable on those of **2** (Table 2), suggesting the same stereostructure of the isoprenoid unit for both **2** and **3**. On the other hand, differences were observed for the signals of the decalin ring . Specifically, the carbon resonances at *δ*_C_ 49.0 (C-1), 38.4 (C-6), 41.8 (C-9), and 18.8 (C-20) in **2** were replaced by resonances at *δ*_C_ 45.6, 35.2, 33.0, and 27.2 in **3**, respectively, indicating a *cis* fusion of the decalin ring [6,8,15]. The relative configuration at C-11, however, could not be conclusively determined due to the free rotation around the C-7–C-11 bond.

**Figure 3:**
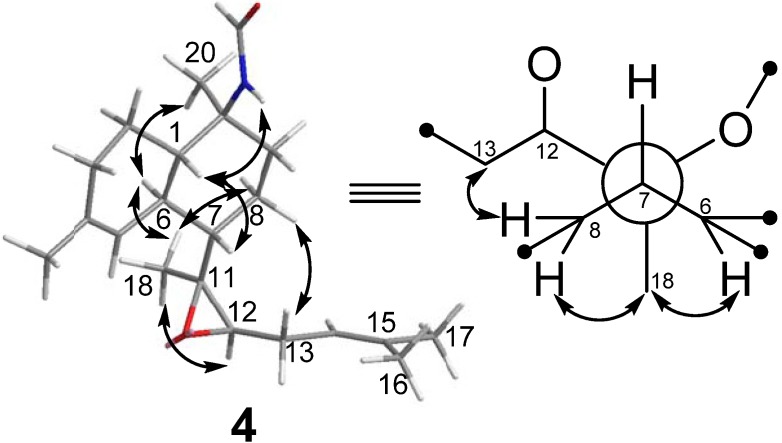
Key NOESY correlations of **4**.

Cavernene D (**4**) displayed a HRESIMS [M + Na]^+^ peak at *m/z* 354.2407 corresponding to a molecular formula of C_21_H_33_O_2_N, implying six degrees of unsaturation. Many similarities of the ^1^H and ^13^C NMR data between **2** and **4** (Table 1 and Table 2) suggested they were structural analogs, with the main differences due to the presence of a trisubstituted epoxide (*δ*_C_ 62.33 and 62.27) in **4** and the absence of two saturated carbons (*δ*_C_ 30.8 and 35.65) in **2**. The C-11/C-12 position of the epoxy group was determined by a COSY correlation of H-12/H-13 and HMBC correlations of H_3_-18/C-7, C-11, and C-12 and H-13/C-11 and C-12. The relative configuration of the decalin ring of **4**, found to agree with **2**, was established by observation of NOESY correlations of NH/H-1, H-1/H-7, and H_3_-20/H-6. The relative configurations of C-7 and C-11 between the conjoined bicyclic ring systems in **4** were assigned as 7S* and 11 S*, respectively, based on NOESY correlations of H_3_-18/H-6, H_3_-18/H-8b, and H-8a/H-13, as shown in the Newman projection (Figure 3). Thus, the epoxy group was determined as in β-orientation.

**Figure 4:**
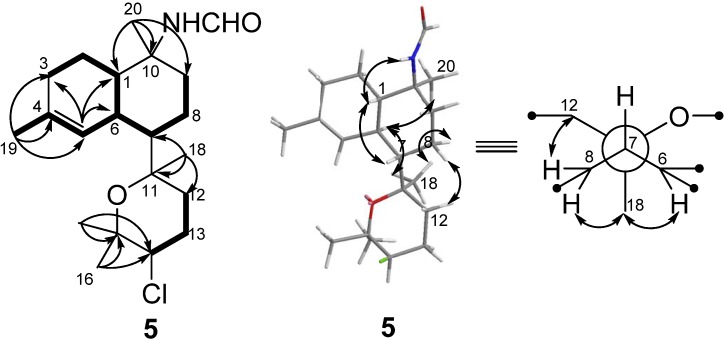
COSY (▬), key HMBC (→), and key NOESY correlations of **5**.

Kalihinene E (**5**) was isolated as colorless needles (MeOH), and given a C_21_H_34_NO_2_Cl molecular formula with five degrees of unsaturation, based on the HRESIMS (*m/z* 368.2354 [M + H]^+^) and NMR spectra. The ESIMS of **5** showed a cluster of isotopic [M + H]^+^ ion peaks at *m/z* 368/370 in a ratio of ~3:1, indicating the presence of a chlorine atom in the molecule. The NMR spectra of 5 revealed the presence of five methyls [*δ*_H_ 1.22 (6H, s), 1.31 (3H, s), 1.39 (3H, s), and 1.65 (3H, s)], an olefinic methine [*δ*_H_ 6.37 (1H, br s), *δ*_C_ 125.7 (CH), and *δ*_C_ 132.4 (qC)], a chlorine-bearing methine [*δ*_H_ 3.93 (1H, m)/*δ*_C_ 64.2], and two oxygenated quaternary carbons (*δ*_C_ 74.2 and 76.9) for the major s-*trans* isomer (Table 1 and Table 2). Analysis of the 2D NMR (HSQC, HMBC, and COSY) data (Figure 4) revealed that **5** possessed the same carbon skeleton as kalihinene X (**11**) and Y (**12**) [15].

**Figure 5:**
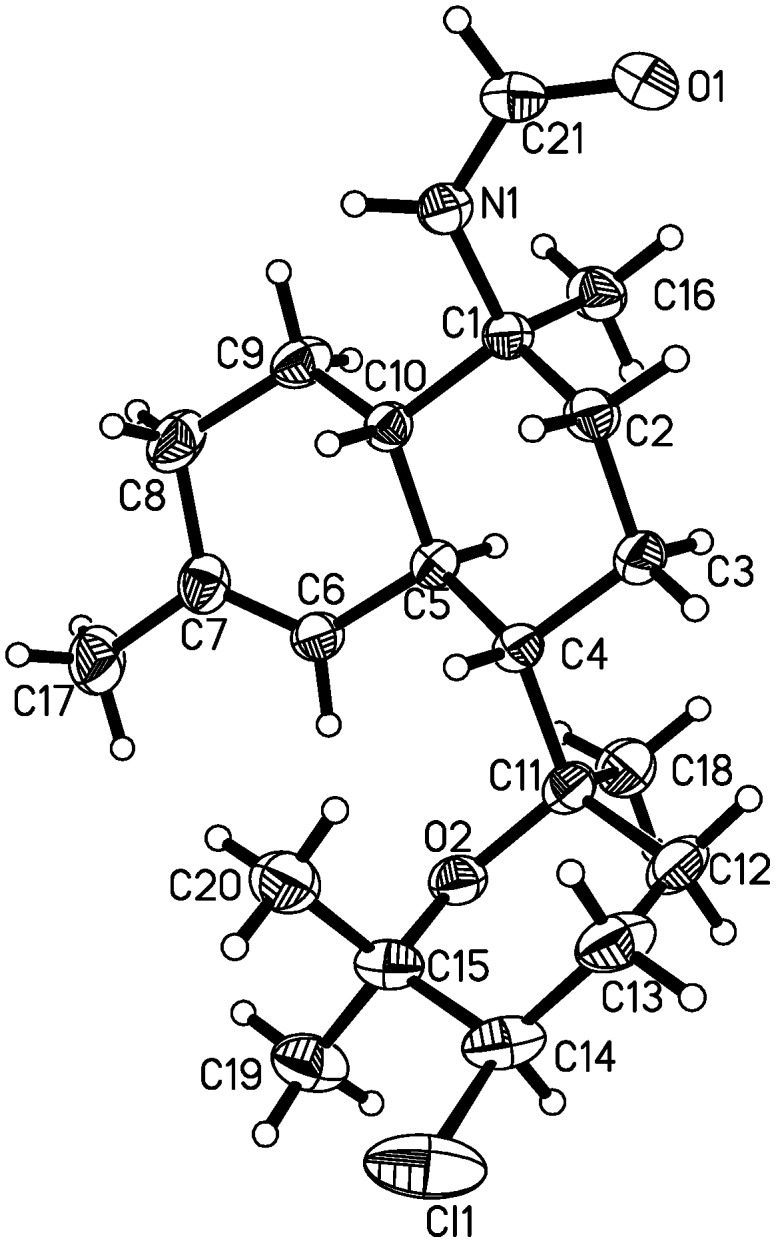
ORTEP drawing of compound **5**.

The relative configuration of the decalin ring in **5** was the same as **12**, inferred from the chemical shifts of C-1 to C-10 and NOESY correlations of H-1/H-7, H_3_-20/H-6, and NH/H-1. A significant difference between **5** and **12** was found in the chemical shift of C-12 (*δ*_C_ 31.0 in **5** instead of *δ*_C_ 38.2 in **12**), which was caused by the *γ-gauche* effect [20,21,22,23,24], indicating an *axial* orientation of Cl–14. The relative configurations of C-7 and C-11 in **5** were determined as S* and R*, respectively, from NOESY correlations of H_3_-18/H-6, H_3_-18/H-8b, and H-8a/H-12, as shown in the Newman projection (Figure 4). Finally, the absolute configuration of **5** was unambiguously determined as 1*S*, 6*S*, 7*S*, 10*S*, 11*R*, and 14*R* by single crystal X-ray diffraction using Cu Kα radiation (Figure 5).

**Figure 6:**
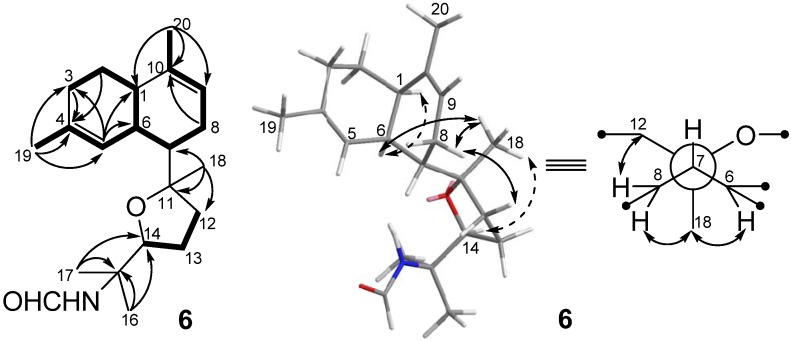
COSY (▬), key HMBC (→), and key NOESY (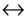) correlations of **6**.

Kalihinene F (**6**) was isolated as a colorless oil and given a C_21_H_33_NO_2_ molecular formula based on HRESIMS measurements (*m/z* 354.2406 [M + Na]^+^) in combination with extensive NMR analysis. The NMR spectra of **6** (Table 1 and Table 2) revealed the presence of five methyls [*δ*_H_ 1.18 (3H, s), 1.20 (3H, s), 1.31 (3H, s), and 1.68 (6H, s)], two trisubstituted double bonds [*δ*_H_ 5.70 (br s), *δ*_C_ 127.1 (CH), and *δ*_C_ 131.1 (qC); *δ*_H_ 5.32 (br s), *δ*_C_ 120.2 (CH), and *δ*_C_ 136.9 (qC)], an oxymethine [*δ*_H_ 3.75 (t, *J* = 7.0 Hz)/*δ*_C_ 84.2], and an oxygenated quaternary carbons (*δ*_C_ 86.4) for the major s-*trans* isomer. The COSY correlations of H_3_-20/H-9/H-8/H-7/H-6/H-1/H-2/H-3 and H-6/H-5/H_3_-19, together with the HMBC correlations of H_3_-19/C-3, C-4, and C-5, H_3_-20/C-1, C-10, and C-9, H-5/C-1, C-3, and C-6, H-2 and H-3/C-4, and H-8/C-10 indicated the presence of a decalin moiety (Figure 6). A tetrahydrofuran ring, attached to the decalin ring at C-7, was established by carbon resonances at *δ*_C_ 84.2 (C-14) and 86.4 (C-11), COSY correlations of H-12/H-13/H-14, HMBC correlations of H_3_-18/C-7, C-11, and C-12 and geminal methyls (H_3_-16 and H_3_-17)/C-14 and C-15, and NOESY correlation between H_3_-18 and H-14. The location of the formamide functionality was assigned to be at C-15 by the observation of doubled singlets for H_3_-16 [*δ*_H_ 1.31 (s) and 1.38 (s) for s-*trans* and s-*cis* isomers, respectively] and H_3_-17 [*δ*_H_ 1.20 (s) and 1.34 (s) for s-*trans* and s-*cis* isomers, respectively] and doubled triplets for H-14 [*δ*_H_ 3.75 (t, 7.0) and 3.80 (t, 7.0) for s-*trans* and s-*cis* isomers, respectively] [6]. The relative configuration of **6** was determined by NOESY correlation of H-1/H-6 and carbon resonances at *δ*_C_ 40.1 (C-1), 36.5 (C-6), and 44.9 (C-7) [6]. NOESY correlations of H_3_-18/H-6, H_3_-18/H-8b, and H-8/H-12b defined the relative configurations of 7S*, 11R*, as shown in the Newman projection (Figure 6).

Kalihypyran C (**7**), a colorless oil, had a molecular formula of C_21_H_31_NO_2_ established by HRESIMS at *m/z* 352.2255 [M + Na]^+^, indicating seven degrees of unsaturation. Its ^1^H and ^13^C NMR spectra (Table 1 and Table 2) showed the presence of three methyls [*δ*_H_ 1.55 (3H, s), 1.62 (3H, s), 1.78 (3H, s)], two trisubstituted double bonds [*δ*_H_ 5.39 (br s), *δ*_C_ 123.9 (CH), and *δ*_C_ 134.5 (qC); *δ*_H_ 5.61 (br s), *δ*_C_ 118.6 (CH), and *δ*_C_ 139.0 (qC)], a disubstituted double bond [*δ*_H_ 4.88 (br s), 5.01 (br s), *δ*_C_ 110.5 (CH_2_), and *δ*_C_ 145.4 (qC)], an oxymethylene [*δ*_H_ 4.16 (s, 2H)/*δ*_C_ 67.63], and an oxymethine *δ*_H_ [3.89 (br s)/δC 77.2] for s-*cis* isomer. Many similarities of the ^1^H and ^13^C NMR data between **7** and **8** suggested they were structural analogs [16], the COSY correlations of H_3_-19/H-5/H-6, H_3_-17/H-16, and H-18/H-12/H-13/H-14 and the HMBC correlations of H_3_-19/C-3, C-4, and C-5, H_3_-20/C-1, C-9, and C-10, H_3_-17/C-14, C-15, and C-16, and H_2_-16/C-14, C-15, and C-17 further confirmed **7** possessed the same carbon skeleton as **8**. The chemical shifts of C-1, C-6, and C-20 for s-*trans* isomers in **7** (*δ*_C_ 45.4, 36.6, and 29.7, respectively) were different from those in **8** (*δ*_C_ 48.9, 39.4, and 18.9, respectively), indicated *cis* fusion of the decalin ring in **7** [16]. The NOESY correlations of H-1/H-6, H-1/H_3_-20, and H-6/H_3_-20 confirmed the relative configurations as 7. However, the relative configuration of H-14 was not determined.

The isolated compounds were assessed for their cytotoxicity against a small panel of human cancer cell lines (human colon cancer cell line HCT-116, human lung epithelial cell line A549, human cervical carcinoma cell line HeLa, human hepatocellular carcinoma cell line QGY-7701, and human mammary cancer cell line MDA-MB-231) using a MTT method, and camptothecin (Shanghai Dibai Chemical Co., Shanghai, China; purity ≥98%) was used as positive control. Compounds 1 and 2 showed moderate cytotoxic activities against HCT-116 with IC_50_ values of 6.31 and 8.99 μM, respectively. Compounds 5 showed cytotoxic activity against HCT-116, HeLa, QGY-7701 and MDA-MB-231 with IC_50_ values of 14.36, 13.36, 17.78 and 12.84 μM, respectively (Table 3). In addition, compounds **1–12** were tested for antifungal activity against fungi *Candida albicans*, *Candida parapsilosis*, *Candida glabrata*, *Cryptococcus neoformans*, *Trichophyton rubrum*, *Microsporum gypseum*, and *Aspergillus fumigatus*. Compound **9** showed weak antifungal activity against *T. rubrum* and *M. gypseum* with MIC values of 8 and 32 μg/mL, respectively. Compound **10** displayed weak antifungal activity against fungi *C. albicans*, *C. neoformans*, *T. rubrum*, and *M. gypseum* with MIC values of 8, 8, 4, and 8 μg/mL, respectively. Ketoconazole (Shanghai Aiyan Chemical Co., Shanghai, China; purity ≥98%) was used as positive control with MIC value ≤0.25 μg/mL. It is worth noting that the isonitrile functionalities in the diterpenes play an important role in their antifungal activity.

**Table 3:** Cytotoxicities of compounds **1**–**12** in five cancer cell lines.

	Cytotoxicity IC_50_ (μM)				
	HCT-116	A549	HeLa	QGY-7701	MDA-MB-231
**1**	6.31	>50	>50	>50	>50
**2**	8.99	>50	>50	>50	>50
**3**	>50	>50	>50	>50	>50
**4**	>50	>50	>50	>50	>50
**5**	14.36	>50	13.36	17.78	12.84
**6**	>50	>50	>50	>50	>50
**7**	>50	>50	>50	>50	>50
**8**	>50	13.09	11.19	13.53	>50
**9**	>50	17.53	14.74	16.39	>50
**10**	>50	6.98	13.30	14.53	6.84
**11**	12.25	8.55	10.59	13.02	7.46
**12**	>50	17.12	10.05	14.41	15.23
**camptothecin**	9.25	2.32	6.98	4.05	0.50

## 3. Experimental Section

### 3.1. General Experimental Procedures

Optical rotation data were determined with a Perkin-Elmer 341 polarimeter (Perkin-Elmer, Inc., Waltham, MA, USA) with a 1 dm cell. UV spectra were collected using a Shimadzu UV 240 spectrophotometer (Shimadzu Corp., Kyoto, Japan). IR spectra were recorded on a Bruker vector 22 spectrometer (Bruker Optics, Inc., Billerica, MA, USA) with KBr pellets. NMR experiments were conducted on Bruker Avance-500 and AMX-400 spectrometers (Bruker Biospin Corp., Billerica, MA, USA). The HRESIMS spectra were acquired with a Waters Q-Tof micro YA019 mass spectrometer (Waters Corp., Milford, MA, USA). X-ray structure analysis was performed on a Bruker SMART APEX-II CCD diffractometer (Bruker Optics, Inc.). Melting points were obtained on an SGW X-4 melting point apparatus (Shanghai Precision & Scientific Instrument Co., Ltd, Shanghai, China). Reversed-phase HPLC was carried out on a YMC-Pack ODS-A column (250 × 10 mm, 5 µm; YMC Co., Ltd., Kyoto, Japan) using a Waters 1525 HPLC instrument with Waters 2998 UV detector and monitored at 210 nm. Silica gel (200–300 mesh, Qingdao Chengyang Ocean Chemical Co., Jinan, China), Sephadex LH-20 (Pharmacia Fine Chemicals, Piscataway, NJ, USA), and YMC ODS-A (50 μm, YMC Co., Ltd, Kyoto, Japan) were used as column packing materials. Fractions were monitored by TLC (HSGF 254, Yantai Huiyou Co., Yantai, China), and spots were visualized by heating silica gel plates sprayed with 12% H_2_SO_4_ in EtOH.

### 3.2. Animal Material

Samples of *A. cavernosa* were collected by hand using scuba around Xisha Islets in the South China Sea in March 2009 and identified by Professor Jin-He Li (Institute of Oceanology, Chinese Academy of Sciences, China). A voucher sample (JHQ-0901) was deposited in the Laboratory of Marine Drugs, Department of Pharmacy, Changzheng Hospital, Second Military Medical University, China.

### 3.3. Extraction and Isolation

The sponge (5.5 kg, wet weight) was extracted with acetone at room temperature five times (5 × 5 L) and the extract was concentrated to a brown oil, which was redissolved in H_2_O (2 L). The aqueous solution was extracted with CH_2_Cl_2_ (4 × 2 L) to afford a CH_2_Cl_2_-soluble extract (89 g). The resulting extract was partitioned between petroleum ether (4 × 1 L) and 90% aqueous MeOH (4 × 1 L) to yield a brownish red oil (49 g), which was subjected to column chromatography (80 × 7.0 cm) on silica gel (1000.5 g) eluting with petroleum ether-acetone gradient (stepwise, 0:1, 50:1, 30:1, 15:1, 8:1, 3:1 to 0:1, 40 L), and finally MeOH, to give eight fractions (Fractions 1–8). Fraction 5 (5.4 g) was fractionated over Sephadex LH-20 (40 × 2000 mm, eluted with CH_2_Cl_2_/MeOH 1:1, 1.5 L), and further fractionated by CC on ODS (RP-18, 30 × 500 mm), eluted with 70%–100% MeOH/H_2_O, to give four subfractions (Fractions 5a–d). Fraction 5a (203.4 mg) was purified by HPLC (YMC-Pack ODS-A, 5 µm, 10 × 250 mm, 2.0 mL/min, UV detection at 210 nm), using MeOH/H_2_O (88:12) as eluent, to yield **7** (2.1 mg, *t_R_* = 40.5 min) and **8** (4.2 mg, *t_R_* = 42.5 min). Fraction 5b (659 mg) was subjected to CC (15 × 300 mm) on silica gel (15 g), using CH_2_Cl_2_/MeOH with increasing polarity (1:0, 100:1, 50:1) as mobile phase, to yield compound **4** (13.3 mg). Fraction 5c (368.0 mg) was purified by HPLC (YMC-Pack ODS-A, 5 µm, 10 × 250 mm, 2.0 mL/min, UV detection at 210 nm), using MeOH/H_2_O (92:8) as eluent, to afford 1 (48.9 mg, *t_R_* = 36.6 min), **3** (4.2 mg, *t_R_* = 40.8 min), and **2** (50.7 mg, *t_R_* = 43.8 min). Fraction 5d (267.5 mg) was chromatographied (15 × 300 mm) on silica gel (15 g, eluted with CH_2_Cl_2_/MeOH 50:1), to obtain **10** (160 mg). Fraction 6 (7.4 g) was applied to Sephadex LH-20 (40 × 2000 mm) eluted with CH_2_Cl_2_/MeOH (1:1) to furnish three subfractions (Fractions 6a and c). Fraction 6b (1.3 g) was subjected to CC on ODS (RP-18, 30 × 500 mm), eluting with 80%–100% MeOH, to give five subfractions (Fractions 6b1–5). Fraction 6b2 (123 mg) was purified by HPLC (YMC-Pack ODS-A, 5 µm, 10 × 250 mm, 2.0 mL/min, UV detection at 210 nm) using MeOH/H_2_O (92:8) as eluent to **9** (1.0 mg, *t_R_* = 37.5 min). Fraction 6b3 (31.5 mg) was fractioned by a Sephadex LH-20 column (15 × 2000 mm) eluting with hexane/CH_2_Cl_2_/MeOH (5:4:1) to afford **6** (4.5 mg). Compound 5 (50.9 mg) was obtained from fraction 6b4 (478.5 mg) by a CC (15 × 300 mm) on silica gel (15 g, eluted with CH_2_Cl_2_/MeOH 30:1). Fraction 7 (8.3 g) was subjected to CC (40 × 2000 mm) on Sephadex LH-20 eluting with MeOH followed by HPLC purification (YMC-Pack ODS-A, 5 µm, 10 × 250 mm, 2.0 mL/min, UV detection at 210 nm) using MeOH/H_2_O (90:10) as eluent to yield **11** (201.5 mg, *t_R_* = 50.4 min) and **12** (197.6 mg, *t_R_* = 56.8 min).

Cavernene A (**1**): colorless oil; [α]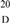 +25.0 (*c* 0.06, MeOH); UV (CH_3_CN) λ_max_ (log ε) < 200 (2.45) nm; IR (KBr) ν_max_ 3323, 3051, 2927, 2856, 1670, 1541, 1457, 1386 cm^−1^; ^1^H and ^13^C NMR data, see Table 1 and Table 2; HRESIMS *m/z* 338.2461 [M + Na]^+^ (calcd for C_21_H_33_NONa, 338.2460).

Cavernene B (**2**): colorless oil; [α]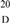 +46.2 (*c* 0.07, MeOH); UV (CH_3_CN) λ_max_ (log ε) < 200 (2.79), 234 (sh, 2.25) nm; IR (KBr) ν_max_ 3295, 3053, 2960, 2926, 2869, 1681, 1537, 1453, 1382 cm^−1^; ^1^H and ^13^C NMR data, see Table 1 and Table 2; HRESIMS *m/z* 340.2617 [M + Na]^+^ (calcd for C_21_H_35_NONa, 340.2616).

Cavernene C (**3**): white needles (MeOH); m.p. 98.0–102.0 °C; [α]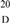 +20.0 (*c* 0.03, MeOH); UV (CH_3_CN) λ_max_ (log ε) < 200 (2.95) nm; IR (KBr) ν_max_ 3304, 3062, 2958, 2926, 2855, 1668, 1538, 1455, 1381 cm^−1^; ^1^H and ^13^C NMR data, see Table 1 and Table 2; HRESIMS *m/z* 340.2614 [M + Na]^+^ (calcd for C_21_H_35_NONa, 340.2616).

Cavernene D (**4**): colorless needles (MeOH); m.p. 112.0–115.0 °C; [α]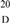 +51.4 (*c* 0.04, MeOH); UV (CH_3_CN) λ_max_ (log ε) < 200 (2.88) nm; IR (KBr) ν_max_ 3296, 3055, 2928, 2857, 1681, 1538, 1453, 1383 cm^−1^; ^1^H and ^13^C NMR data, see Table 1 and Table 2; HRESIMS *m/z* 354.2407 [M + Na]^+^ (calcd for C_21_H_33_NO_2_Na, 354.2409).

Kalihinene E (**5**): colorless needles (MeOH); m.p. 185.0–190.0 °C; [α]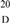 +25.0 (*c* 0.04, MeOH); UV (CH_3_CN) λ_max_ (log ε) < 200 (2.94) nm; IR (KBr) ν_max_ 3295, 3074, 2929, 2868, 1682, 1541, 1451, 1381 cm^−1^; ^1^H and ^13^C NMR data, see Table 1 and Table 2; HRESIMS *m/z* 368.2354 [M + H]^+^ (calcd for C_21_H_35_NO_2_Cl, 368.2356).

Kalihinene F (**6**): colorless oil; [α]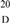 +2.5 (*c* 0.08, MeOH); UV (CH_3_CN) λ_max_ (log ε) < 200 (2.63), 232 (sh, 2.12) nm; IR (KBr) ν_max_ 3216, 3057, 2966, 2929, 1686, 1451, 1379, 1311 cm^−1^; ^1^H and ^13^C NMR data, see Table 1 and Table 2; HRESIMS *m/z* 354.2406 [M + Na]^+^ (calcd for C_21_H_33_NO_2_Na, 354.2409).

Kalihipyran C (**7**): colorless oil; [α]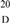 +24.6 (*c* 0.07, MeOH); UV (CH_3_CN) λ_max_ (log ε) < 200 (2.87) nm; IR (KBr) ν_max_ 3296, 3055, 2925, 2855, 1668, 1537, 1454, 1377 cm^−1^; ^1^H and ^13^C NMR data, see Table 1 and Table 2; HRESIMS *m/z* 352.2255 [M + Na]^+^ (calcd for C_21_H_31_NO_2_Na, 352.2252).

X-ray Crystallographic Analysis Data of Kalihinene E (**5**): Colorless needles, C_21_H_34_NO_2_Cl, *MW* = 367.94, monoclinic space group *P*2_1_, *a* = 6.499 (2) Å, *b* = 7.952 (2) Å, *c* = 20.158 (4) Å, V = 1036.1 (4) Å3, *Z* = 2, *D*_calcd_ = 1.179 g/cm^3^, and *F* (000) = 400. A single crystal of dimensions 0.05 × 0.13 × 0.18 mm was used for X-ray measurements and data collected on a Bruker SMART APEX-II CCD diffractometer using Cu Kα radiation and up to *θ* = 67.3 at 293 K. A total of 5789 reflections were collected, of which 3435 independent reflections were measured having an Rint of 0.0220, final R indices of *I* > 2σ (*I*), R1=0.0426, wR2 = 0.1163, R indices for all data R1 = 0.0445, and wR2 = 0.1184. The crystal structure solution was achieved using direct methods, as implemented with the SHELX-97 software program. The refinment method was full-matrix least-square on *F*^2^, goodness-of-fit on *F*^2^ was 1.042, and the largest difference peak and hole were 0.237 and −0.196 e. Å-3. The absolute structure was determined giving a Flack parameter of 0.07 (2). The X-ray diffraction material has been deposited in the Cambridge Crystallographic Data Center (CCCD No. 847695).

### 3.4. Cytotoxicity Assay

Cytotoxic activity was evaluated by a MTT method as described previously [25]. Cells were cultured in RPMI-1640 supplemented with 10% fetal bovine serum in 5% CO_2_ at 37 °C. An aliquot (200 μL) of these cell suspensions at a density of 5 × 10^−4^ cell mL^−1^ was plated in 96-well microtiter plates and incubated for 24 h under the above conditions. 2 μL of the test compound in DMSO at different concentrations was added to each well for 48 h, and then incubated with 1 mg/mL MTT for 4 h. The formazan dye product was measured by the absorbance at 570 nm on a microplate reader. IC_50_ values were calculated by Reed and Muench’s method.

### 3.5. Antifungal Activity Assay

Antifungal activity was determined by the broth macrodilution method following the National Center for Clinical Laboratory Standards (NCCLS) recommendations against the following strains: *Candida albicans*, *Candida parapsilosis*, *Candida glabrata*, *Cryptococcus neoformans*, *Trichophyton rubrum*, *Microsporum gypseum*, and *Aspergillus fumigates* [26,27]. Briefly, bacterial strains were grown aerobically at 30 °C in SDA for 16–20 h in an orbital shaker. A set of tube swith different concentrations of compounds **1–12** prepared in RPMI 1640 were next inoculated with the microorganisms and incubated 24 h for *C. albicans*, *C. parapsilosis*, *C. glabrata*, and *A. fumigatus*, 72 h for *C. neoformans*, and 4–7 days for *T. rubrum* and *M. gypseum*. Broth tubes that appeared turbid were indicative of bacterial growth, while tubes that remained clear indicated no growth. The MIC, defined as the lowest concentration of inhibitory compound at which no growth was observed, was evaluated in triplicate for each compound (within the range 1.25–640 μg/mL). Cultures prepared under the same conditions but without compounds and cultures with the same proportions of DMSO (<1%) were used as controls. The growth of broth tubes without turbidity was further examined by counting the viable cells on the SDA plates.

## 4. Conclusions

A chemical investigation of the marine sponge of *Acanthella cavernosa* led to the isolation of seven new fomamido-diterpenes, cavernenes A–D (**1–4**), kalihinenes E and F (**5**,**6**), and kalihipyran C (**7**), and five known compounds, kalihipyran A (**8**), 15-formamido-kalihinene (**9**), 10-formamido-kalihinene (**10**), and kalihinenes X (**11**) and Y (**12**). The absolute configuration of **5** was determined by single X-ray diffraction. The isolated compounds showed modest cytotoxicity against a small panel of human cancer cell lines, HCT-116, A549, HeLa, QGY-7701 and MDA-MB-231. Compounds **9** and **10** displayed antifungal activity against *Trichophyton rubrum* and *Microsporum gypseum*, *Candida albicans*, and *Cryptococcus neoformans*.

## Supplementary Files

Supplementary File 1: PDF-Document (PDF, 9743 KB)

## References

[1] Chang C.W.J., Patra A., Baker J.A., Scheuer P.J. (1987). Kalihinols, multifunctional ditepenoid anntibiotics from marine sponges *Acanthella* spp. J. Am. Chem. Soc..

[2] Trimurtulu G., Faulkner D.J. (1994). Six new ditepene isonitriles from the sponge *Acanthella cavernosa*. J. Nat. Prod..

[3] Clark R.J., Stapleton B.L., Garson M.J. (2000). New isocyano and isothiocyanato terpene metabolites from the topical marine sponge *Acanthella cavernosa*. Tetrahedron.

[4] Angerhofer C.K., Pezzuto J.M. (1992). Antimalarial activity of sesquiterpenes from the marine sponge *Acanthella klethra*. J. Nat. Prod..

[5] Jumaryatno P., Stapleton B.L., Hooper J.N.A., Brecknell D.J., Blanchfield J.T., Garson M.J. (2007). A comparison of sesquiterpene scaffolds across different populations of the tropical marine sponge *Acanthella cavernosa*. J. Nat. Prod..

[6] Rodriguez J., Nieto R.M., Huter L.M., Diaz M.C., Crews P. (1994). Variation among known kalihinol and new kalihinene diterpenes from the sponge *Acanthella cavernosa*. Tetrahedron.

[7] Sun J.Z., Chen K.S., Yao L.G., Liu H.L., Guo Y.W. (2009). A new kalihinol diterpene from the hainan sponge *Acanthella* sp. Arch. Pharm. Res..

[8] Fusetani N., Yasumura E., Kawai H., Natori T., Binnen L., Clardy J. (1990). Kalihinene and isokalihinol B, cytotoxic diterpene isonitriles from the marine sponge *Acanthella klethra*. Tetrahedron Lett..

[9] Omar S., Albert C., Fanni T., Crews P. (1988). Polyfunctional diterpene isonitriles from marine sponge *Acanthella cavernosa*. J. Org. Chem..

[10] Alvi K.A., Tenenbaum L., Crews P. (1991). Anthelmintic polyfunctional nitrogen-containing terpenoids from marine sponges. J. Nat. Prod..

[11] Miyaoka H., Shimomura M., Kimura H., Yamada Y. (1998). Antimalarial activity of kalihinol A and new relative diterpenoids from the Okinawan sponge, *Acanthella* sp. Tetrahedron.

[12] Bugni T.S., Singh M.P., Chen L., Arias D.A., Harper M.K., Greenstein M., Maiese W.M., Concepcion G.P., Mangalindan G.C., Ireland C.M. (2004). Kalihinols from two *Acanthella cavernosa* sponges: Inhibitors of bacterial folate biosynthesis. Tetrahedron.

[13] Chang C.W.J., Patra A., Roll D.M., Scheuer P.J. (1984). Kalihinol-A, a highly functionalized diisocyano diterpenoid antibiotic from a sponge. J. Am. Chem. Soc..

[14] Patra A., Chang C.W.J., Scheuer P.A., Van Dayne D.G., Matsumoto G.K., Clardy J. (1984). An unprecedented triisocyano diterpenoid antibiotic from a sponge. J. Am. Chem. Soc..

[15] Okino T., Yoshimura E., Hirota H., Fusetani N. (1995). Antifouling kalihinenes from the marine sponge *Acanthella cavernosa*. Tedrahedron Lett..

[16] Okino T., Yoshimura E., Hirota H., Fusetani N. (1996). New antifouling kalihipyrans from the marine sponge *Acanthella cavernosa*. J. Nat. Prod..

[17] Hirota H., Tomono Y., Fusetani N. (1996). Terpenoids with antifouing activity against *Barnacle* larvae from the marine sponge *Acanthella cavernosa*. Tetrahedron.

[18] Xu Y., Li N., Jiao W.H., Wang R.P., Peng Y., Qi S.H., Song S.J., Chen W.S., Lin H.W. (2012). Antifouling and cytotoxic constituents from the South China Sea sponge *Acanthella cavernosa*. Tetrahedron.

[19] Garson M.J., Simpson J.S. (2004). Marine isocyanides and related natural products—Structure, biosynthesis and ecology. Nat. Prod. Rep..

[20] Dorman D.E., Roberts J.D. (1970). Nuclear magnetic resonance spectroscopy. Carbon-13 spectra of some pentose and hexose aldopyranoses. J. Am. Chem. Soc..

[21] Dalling D.K., Grant D.M. (1967). Carbon-13 magnetic resonance. IX. The methylcyclohexanes. J. Am. Chem. Soc..

[22] Ribeiro D.S., Olivato P.R., Rittner R. (2000). Axial/equatorial populations in α-hetero-substituted cyclohexanone Oximes and *O*-methyl oximes. Magn. Reson. Chem..

[23] Gultekin D.D., Tasxkesenligil Y., Dastan A., Balci M. (2008). Bromination of norbornene derivatives: Synthesis of brominated norbornanes and norbornenes. Tetrahedron.

[24] Tonelli A.E., Schilling F.C. (1984). ^13^C NMR chemical shifts and the microstructure of propylene-vinyl chloride copolymers with low propylene content. Macromolecules.

[25] Zhang H.J., Yi Y.H., Yang G.J., Hu M.Y., Cao G.D., Yang F., Lin H.W. (2010). Proline-containing cyclopeptides from the marine sponge *Phakellia fusca*. J. Nat. Prod..

[26] Clinical and Laboratory Standards Institute (2008). Reference Method for Broth Dilution Antifungal Susceptibility Testing of Filamentous Fungi: Approved Standard; NCCLS M38-A2.

[27] Clinical and Laboratory Standards Institute (2009). Reference Method for Broth Dilution Antifungal Susceptibility Testing of Yeasts: Approved Standard; NCCLS M27-A3.

