# Supplementary material for: Formamido-Diterpenes from the South China Sea Sponge Acanthella cavernosa
Source: Mar Drugs. 2012 Jul 2;10(7):1445–58. doi: 10.3390/md10071445 (PMC3407923; doi:10.3390/md10071445)

Supporting Information

Figure S1. ^{1}H NMR spectrum of cavernene A (**1**) in $CDCl_3$.
Figure S2. ^{13}C NMR spectrum of cavernene A (**1**) in $CDCl_3$.
Figure S3. HSQC spectrum of cavernene A (**1**) in $CDCl_3$.
Figure S4. HMBC spectrum of cavernene A (**1**) in $CDCl_3$.
Figure S5. ^{1}H-^{1}H COSY spectrum of cavernene A (**1**) in $CDCl_3$.
Figure S6. NOESY spectrum of cavernene A (**1**) in $CDCl_3$.
Figure S7. IR spectrum of cavernene A (**1**).
Figure S8. HRESIMS of cavernene A (**1**).
Figure S9. UV spectrum of cavernene A (**1**).
Figure S10. ^{1}H NMR spectrum of cavernene B (**2**) in $CDCl_3$.
Figure S11. ^{13}C NMR spectrum of cavernene B (**2**) in $CDCl_3$.
Figure S12. HSQC spectrum of cavernene B (**2**) in $CDCl_3$.
Figure S13. HMBC spectrum of cavernene B (**2**) in $CDCl_3$.
Figure S14. ^{1}H-^{1}H COSY spectrum of cavernene B (**2**) in $CDCl_3$.
Figure S15. NOESY spectrum of cavernene B (**2**) in $CDCl_3$.
Figure S16. IR spectrum of cavernene B (**2**).
Figure S17. HRESIMS of cavernene B (**2**).
Figure S18. UV spectrum of cavernene B (**2**).
Figure S19. ^{1}H NMR spectrum of cavernene C (**3**) in $CDCl_3$.
Figure S20. ^{13}C NMR spectrum of cavernene C (**3**) in $CDCl_3$.
Figure S21. HSQC spectrum of cavernene C (**3**) in $CDCl_3$.
Figure S22. HMBC spectrum of cavernene C (**3**) in $CDCl_3$.
Figure S23. ^{1}H-^{1}H COSY spectrum of cavernene C (**3**) in $CDCl_3$.
Figure S24. NOESY spectrum of cavernene C (**3**) in $CDCl_3$.
Figure S25. IR spectrum of cavernene C (**3**).
Figure S26. HRESIMS of cavernene C (**3**).
Figure S27. UV spectrum of cavernene C (**3**).
Figure S28. ^{1}H NMR spectrum of cavernene D (**4**) in $CDCl_3$.
Figure S29. ^{13}C NMR spectrum of cavernene D (**4**) in $CDCl_3$.
Figure S30. HSQC spectrum of cavernene D (**4**) in $CDCl_3$.
Figure S31. HMBC spectrum of cavernene D (**4**) in $CDCl_3$.
Figure S32. ^{1}H-^{1}H COSY spectrum of cavernene D (**4**) in $CDCl_3$.
Figure S33. NOESY spectrum of cavernene D (**4**) in $CDCl_3$.
Figure S34. IR spectrum of cavernene D (**4**).
Figure S35. HRESIMS of cavernene D (**4**).
Figure S36. UV spectrum of cavernene D (**4**).
Figure S37. ^{1}H NMR spectrum of kalihinene E (**5**) in $CDCl_3$.
Figure S38. ^{13}C NMR spectrum of kalihinene E (**5**) in $CDCl_3$.
Figure S39. HSQC spectrum of kalihinene E (**5**) in $CDCl_3$.
Figure S40. HMBC spectrum of kalihinene E (**5**) in $CDCl_3$.
Figure S41. ^{1}H-^{1}H COSY spectrum of kalihinene E (**5**) in $CDCl_3$.

Figure S42. NOESY spectrum of kalihinene E (**5**) in $CDCl_3$.
Figure S43. IR spectrum of kalihinene E (**5**).
Figure S44. HRESIMS of kalihinene E (**5**).
Figure S45. UV spectrum of kalihinene E (**5**).
Figure S46. ^1H NMR spectrum of kalihinene F (**6**) in $CDCl_3$.
Figure S47. ^{13}C NMR spectrum of kalihinene F (**6**) in $CDCl_3$.
Figure S48. HSQC spectrum of kalihinene F (**6**) in $CDCl_3$.
Figure S49. HMBC spectrum of kalihinene F (**6**) in $CDCl_3$.
Figure S50. ^1H-^1H COSY spectrum of kalihinene F (**6**) in $CDCl_3$.
Figure S51. NOESY spectrum of kalihinene F (**6**) in $CDCl_3$.
Figure S52. IR spectrum of kalihinene F (**6**).
Figure S53. HRESIMS of kalihinene F (**6**).
Figure S54. UV spectrum of kalihinene F (**6**).
Figure S55. ^1H NMR spectrum of kalihipyran C (**7**) in $CDCl_3$.
Figure S56. ^{13}C NMR spectrum of kalihipyran C (**7**) in $CDCl_3$.
Figure S57. HSQC spectrum of kalihipyran C (**7**) in $CDCl_3$.
Figure S58. HMBC spectrum of kalihipyran C (**7**) in $CDCl_3$.
Figure S59. ^1H-^1H COSY spectrum of kalihipyran C (**7**) in $CDCl_3$.
Figure S60. NOESY spectrum of kalihipyran C (**7**) in $CDCl_3$.
Figure S61. IR spectrum of kalihipyran C (**7**).
Figure S62. HRESIMS of kalihipyran C (**7**).
Figure S63. UV spectrum kalihipyran C (**7**).

Figure S1. ^{1}H NMR spectrum of cavernene A (**1**) in $CDCl_3$.

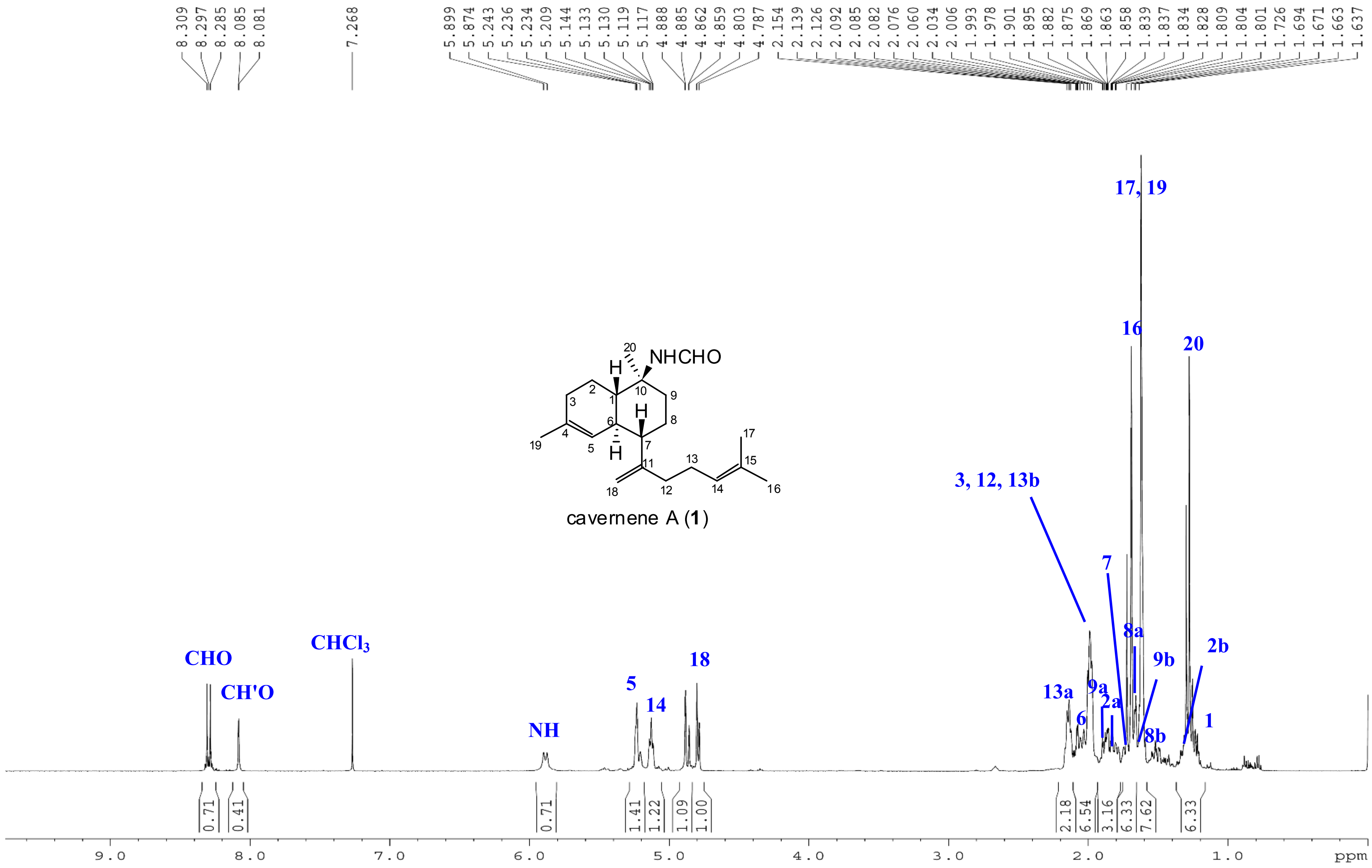

Figure S2. ^{13}C NMR spectrum of cavernene A (**1**) in $CDCl_3$.

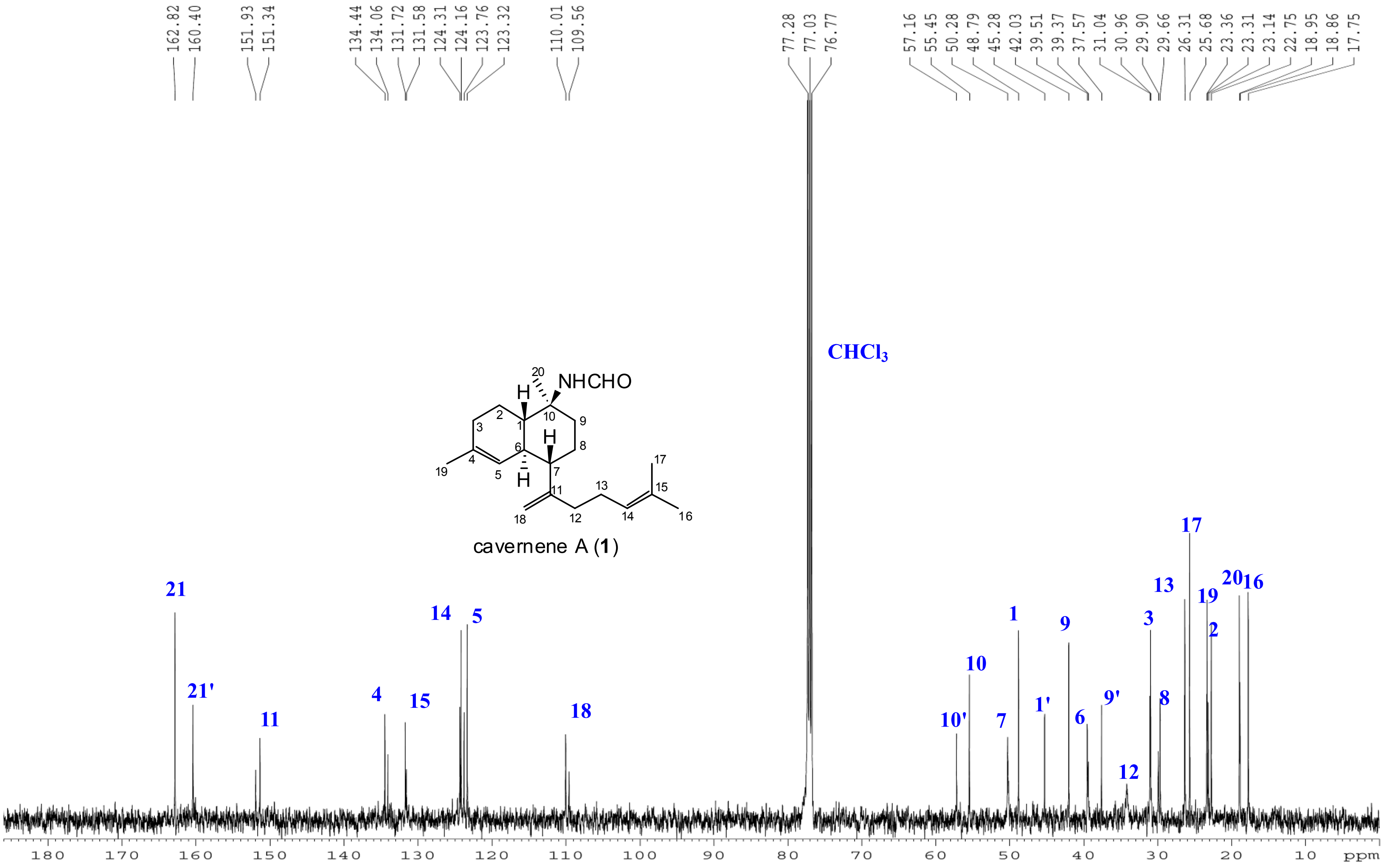

Figure S3. HSQC spectrum of cavernene A (**1**) in $CDCl_3$.

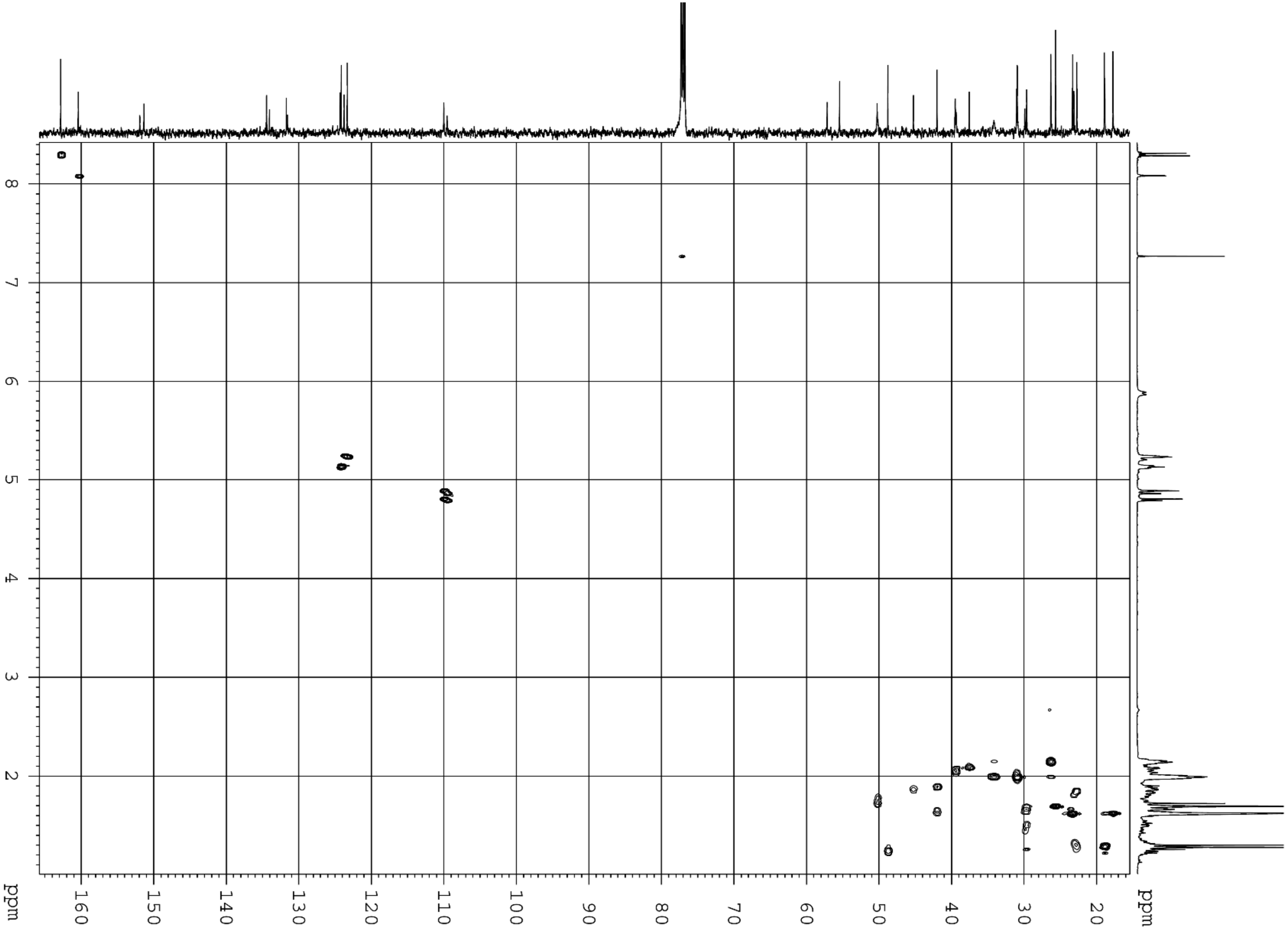

Figure S4. HMBC spectrum of cavernene A (**1**) in $CDCl_3$.

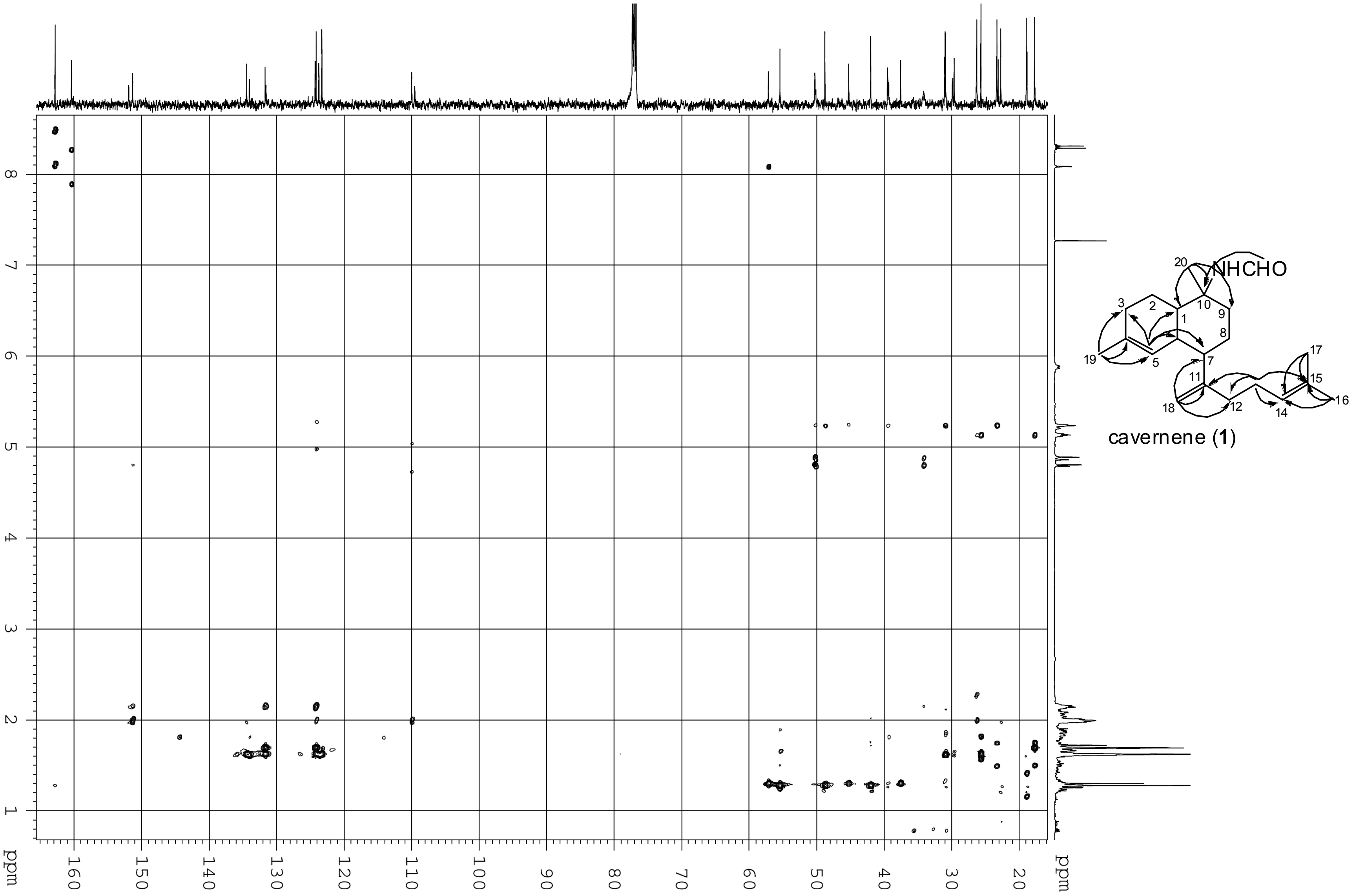

Figure S5. ^{1}H-^{1}H COSY spectrum of cavernene A (**1**) in $CDCl_3$.

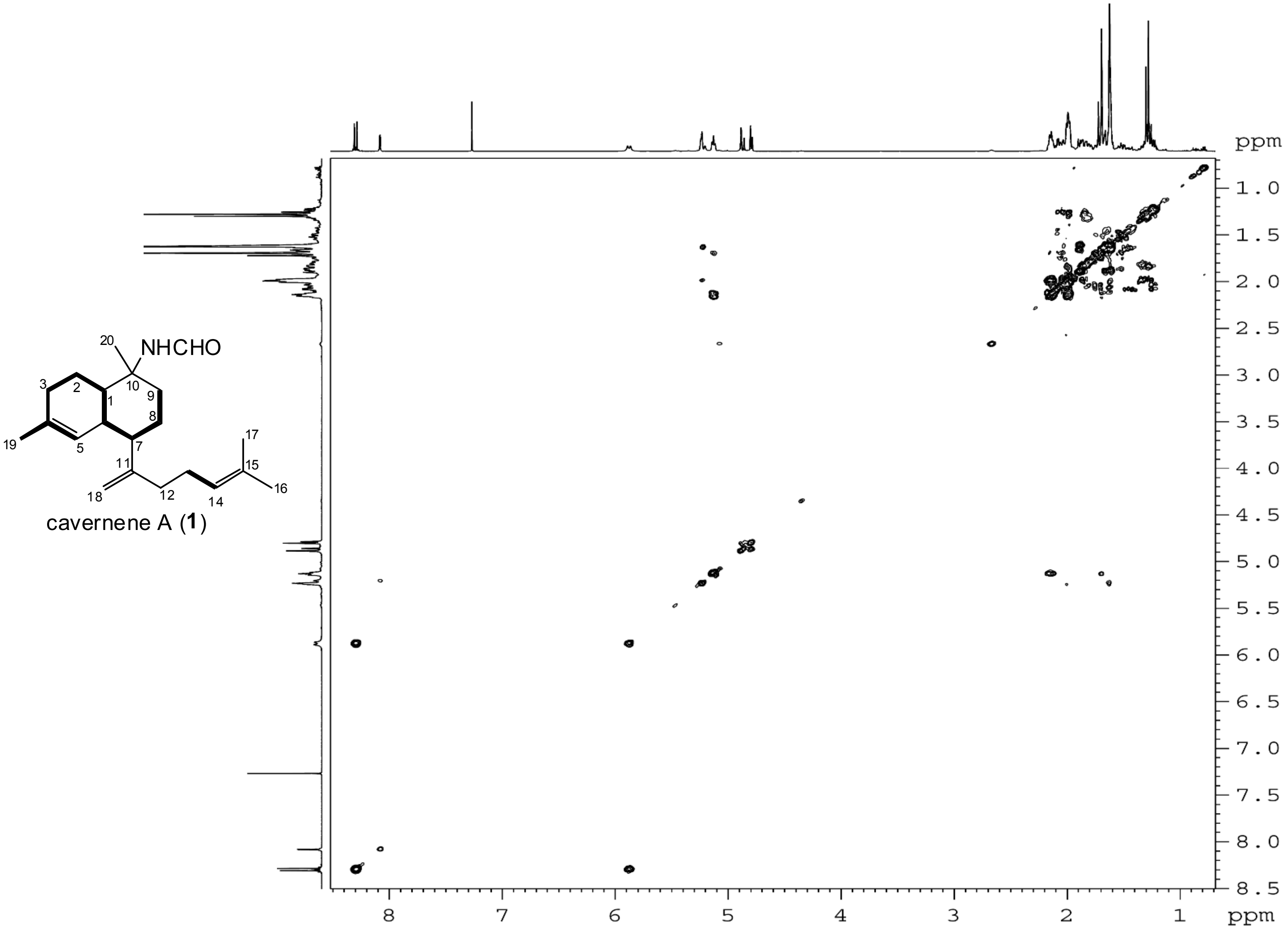

cavernene A (**1**)

Figure S6. NOESY spectrum of cavernene A (**1**) in $CDCl_3$.

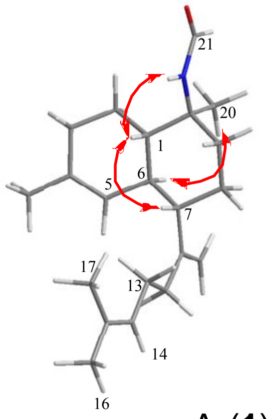

cavernene A (**1**)

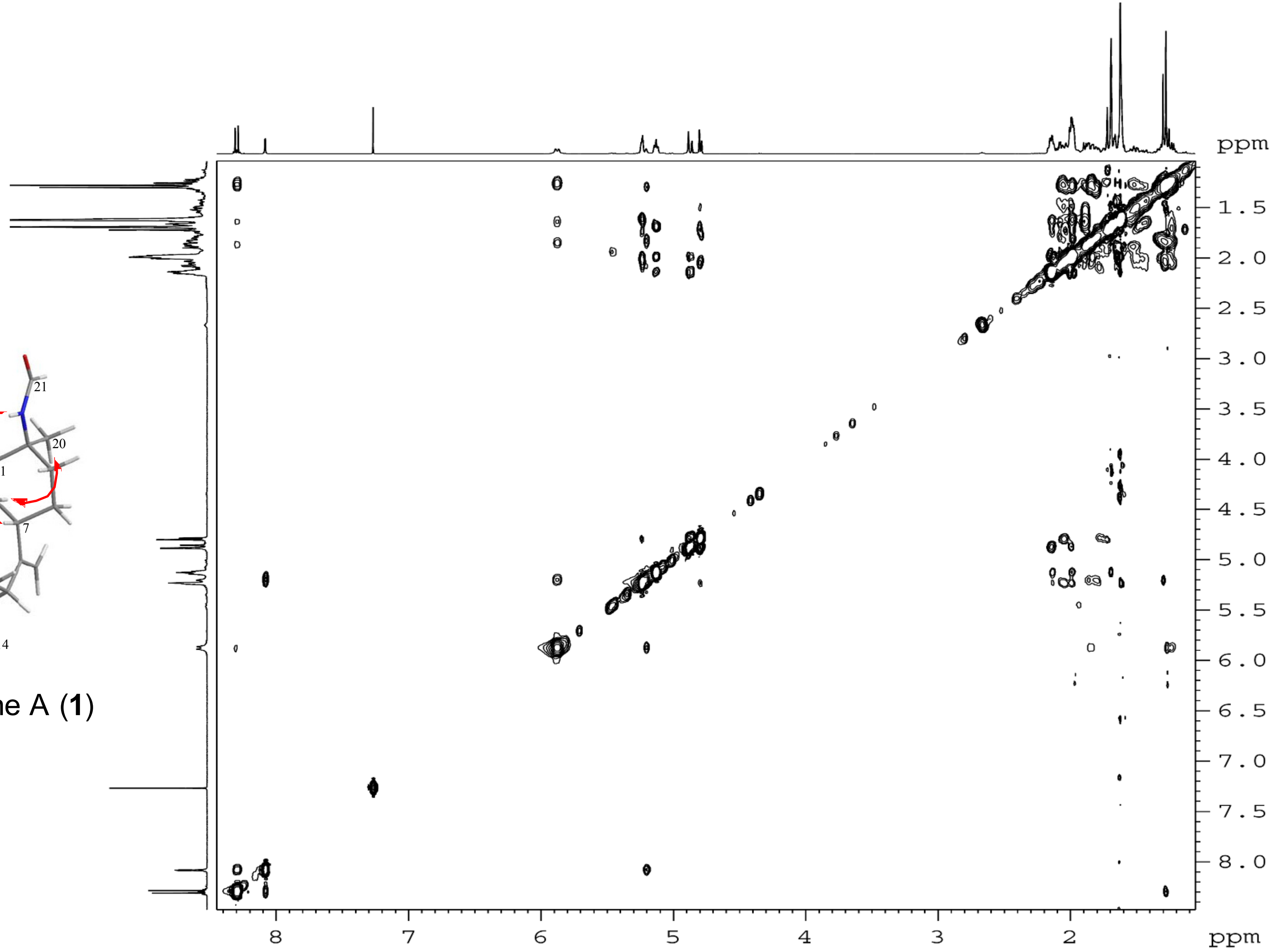

Figure S7. IR spectrum of cavernene A (**1**).

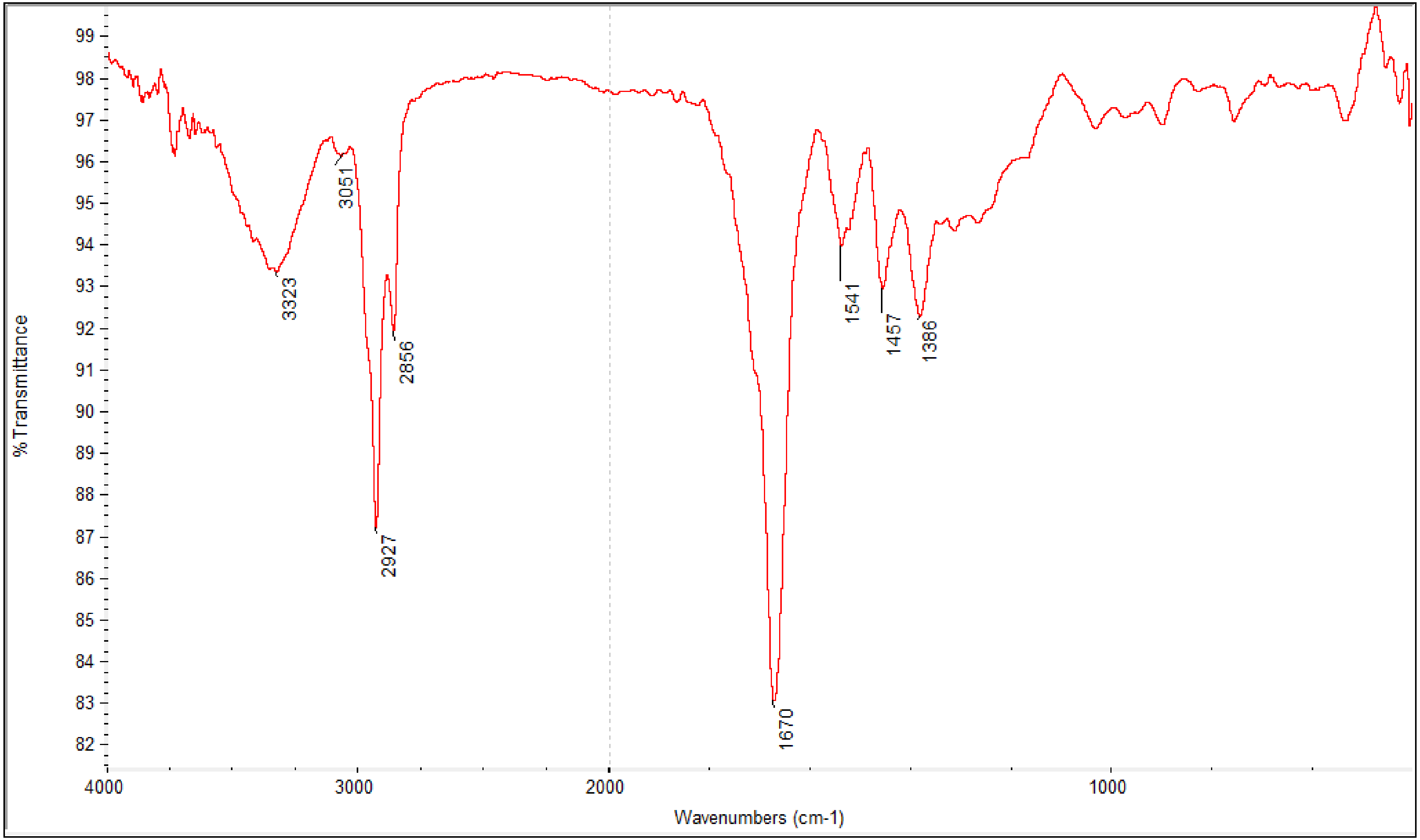

Figure S8. HRESIMS of cavernene A (**1**).

Elemental Composition Report

Page 1

Tolerance = 10.0 PPM / DBE: min = -1.5, max = 50.0
Selected filters: None

Monoisotopic Mass, Even Electron Ions
4 formula(e) evaluated with 1 results within limits (up to 50 closest results for each mass)
Elements Used:
C: 5-25 H: 10-40 N: 1-1 O: 1-2 Na: 1-1

SIPI
Q-Tof micro
YA019
20-May-2011,12:59:41
0.00000000
TOF MS ES+
1.70e4

WQ11-310H 21 (0.740) AM (Cen,6, 80.00, Ar,5000.0,325.16,0.70); Sm (SG, 2x3.00); Cm (15:36)

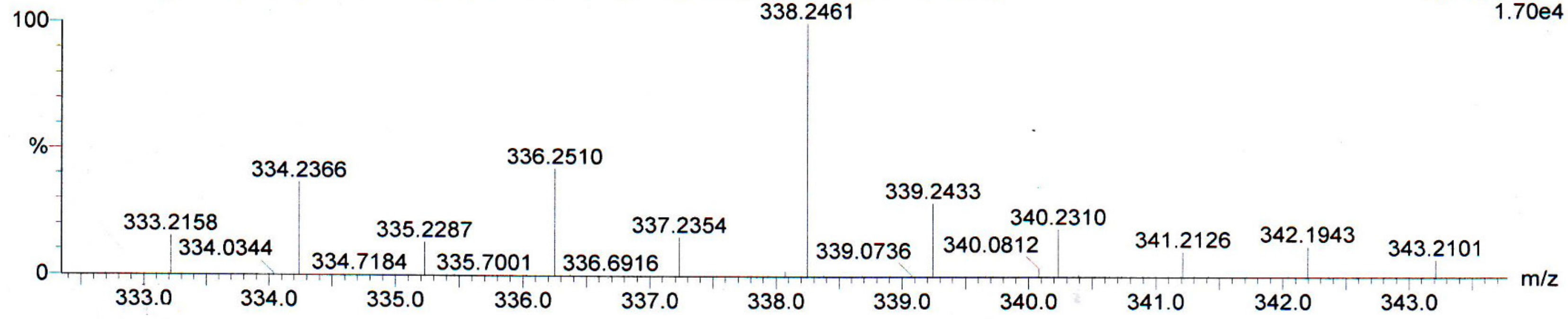

Minimum:	45.00				-1.5		
Maximum:	100.00		5.0	10.0	50.0		
Mass	RA	Calc. Mass	mDa	PPM	DBE	i-FIT	Formula
338.2461	100.00	338.2460	0.1	0.3	5.5	1181.0	C21 H33 N O Na

Figure S9. UV spectrum of cavernene A (**1**).

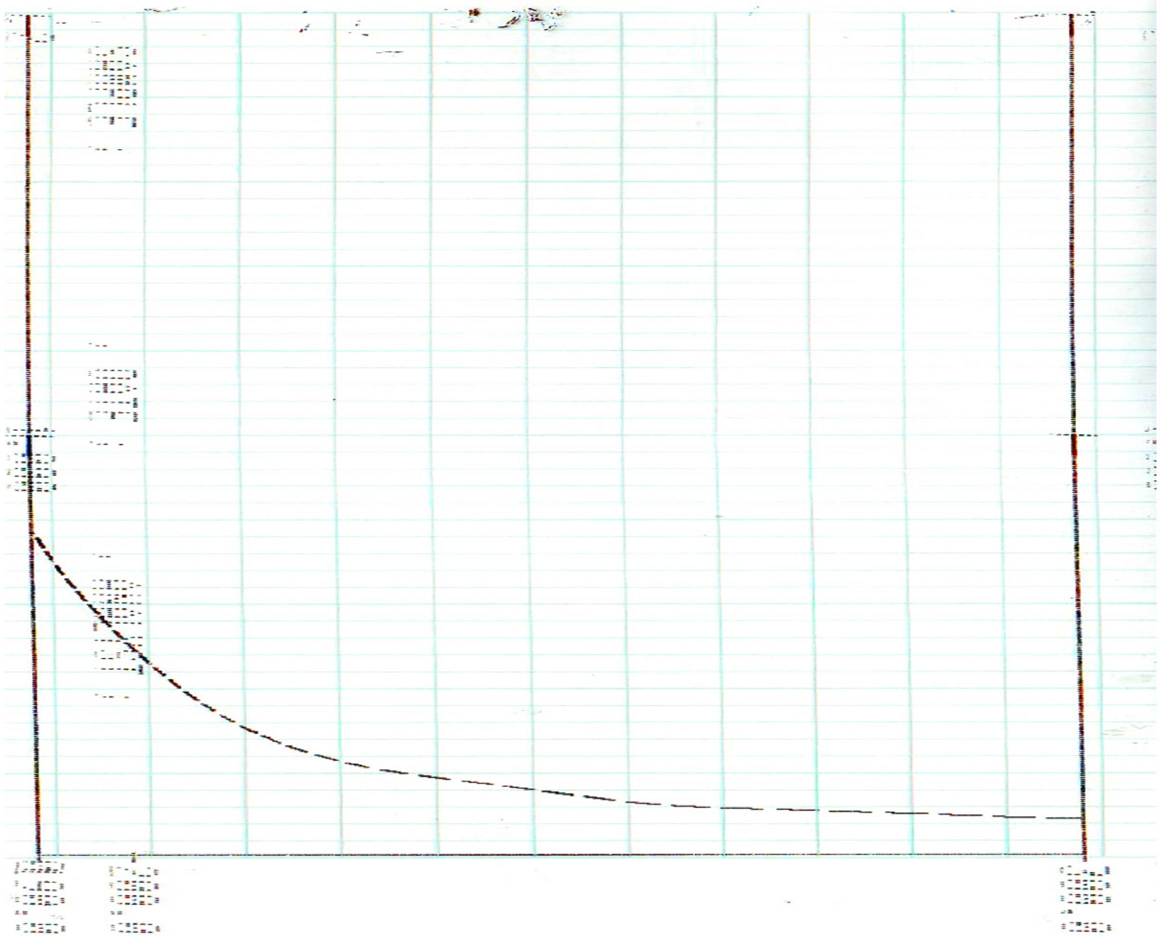

Figure S10. ^{1}H NMR spectrum of cavernene B (**2**) in $CDCl_3$.

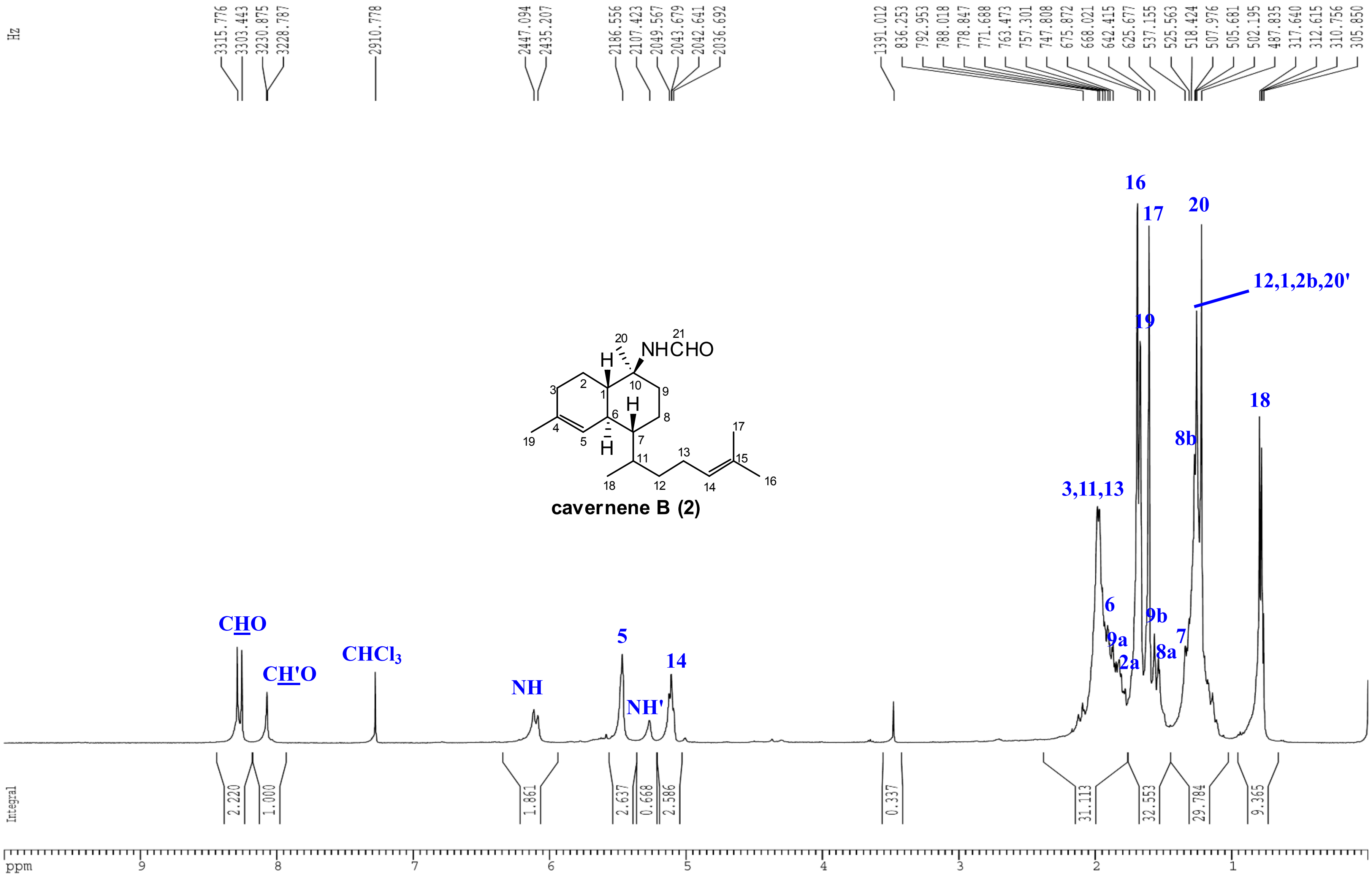

Figure S11. ^{13}C NMR spectrum of cavernene B (**2**) in $CDCl_3$.

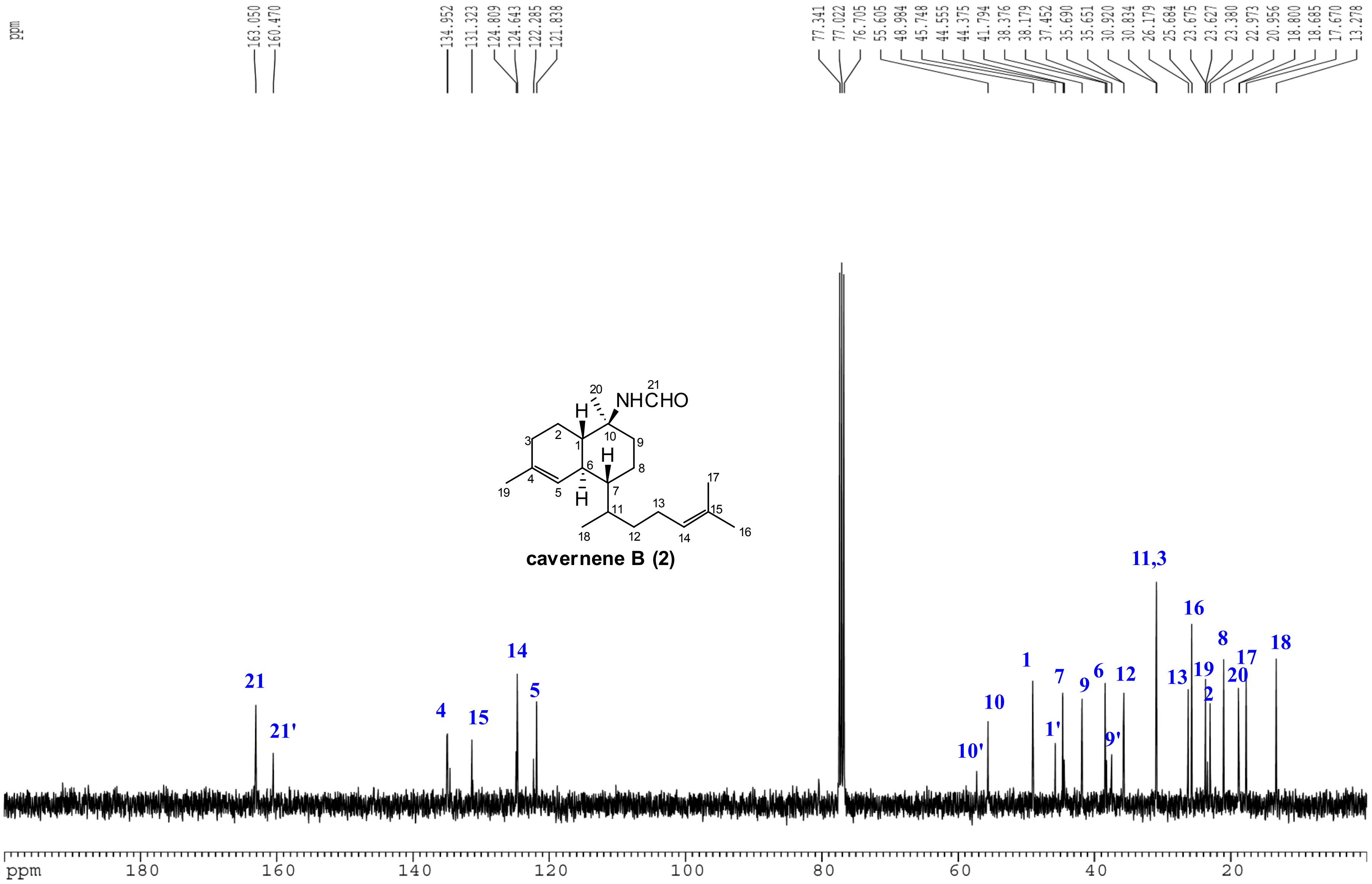

Figure S12. HSQC spectrum of cavernene B (**2**) in $CDCl_3$.

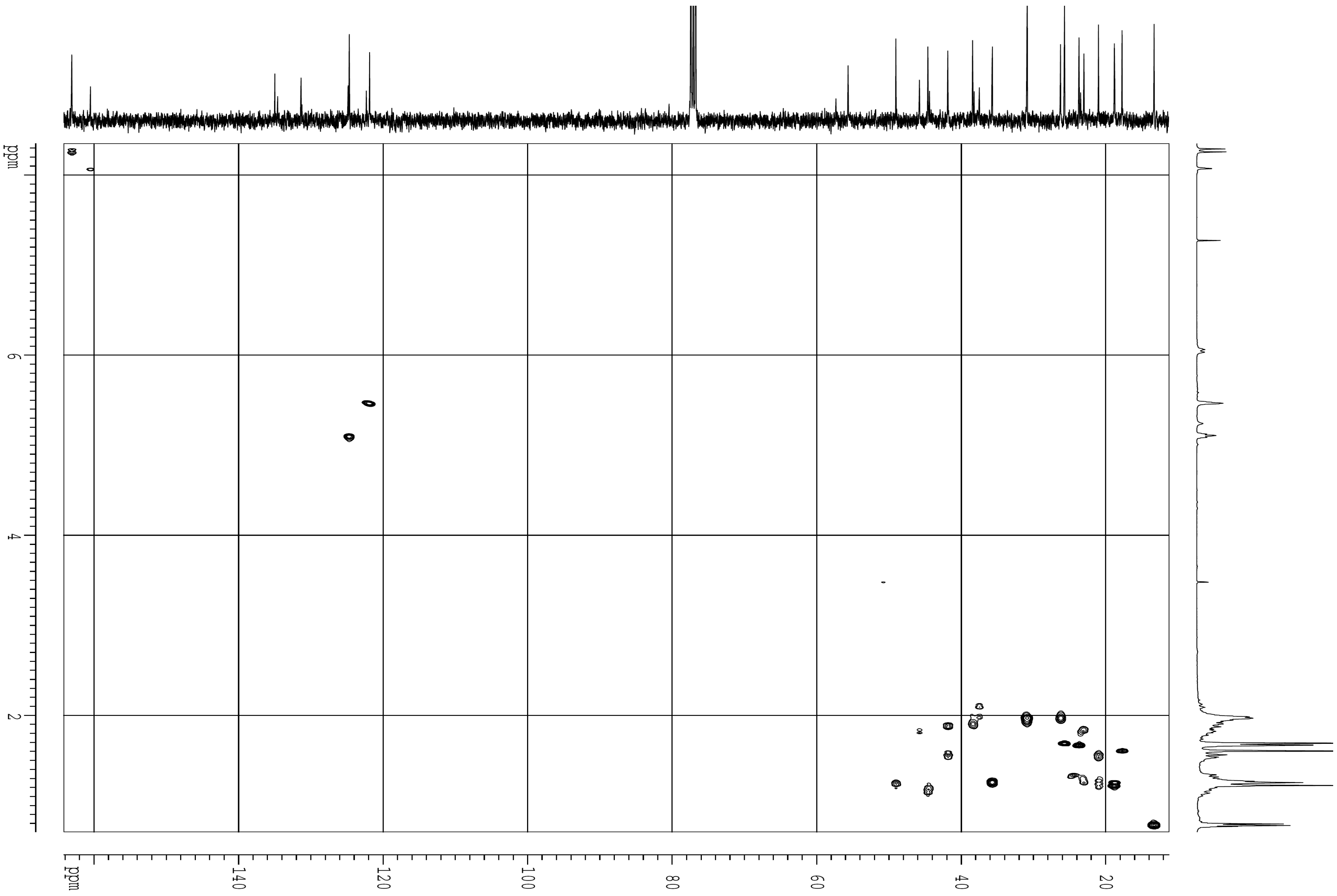

Figure S13. HMBC spectrum of cavernene B (**2**) in $CDCl_3$.

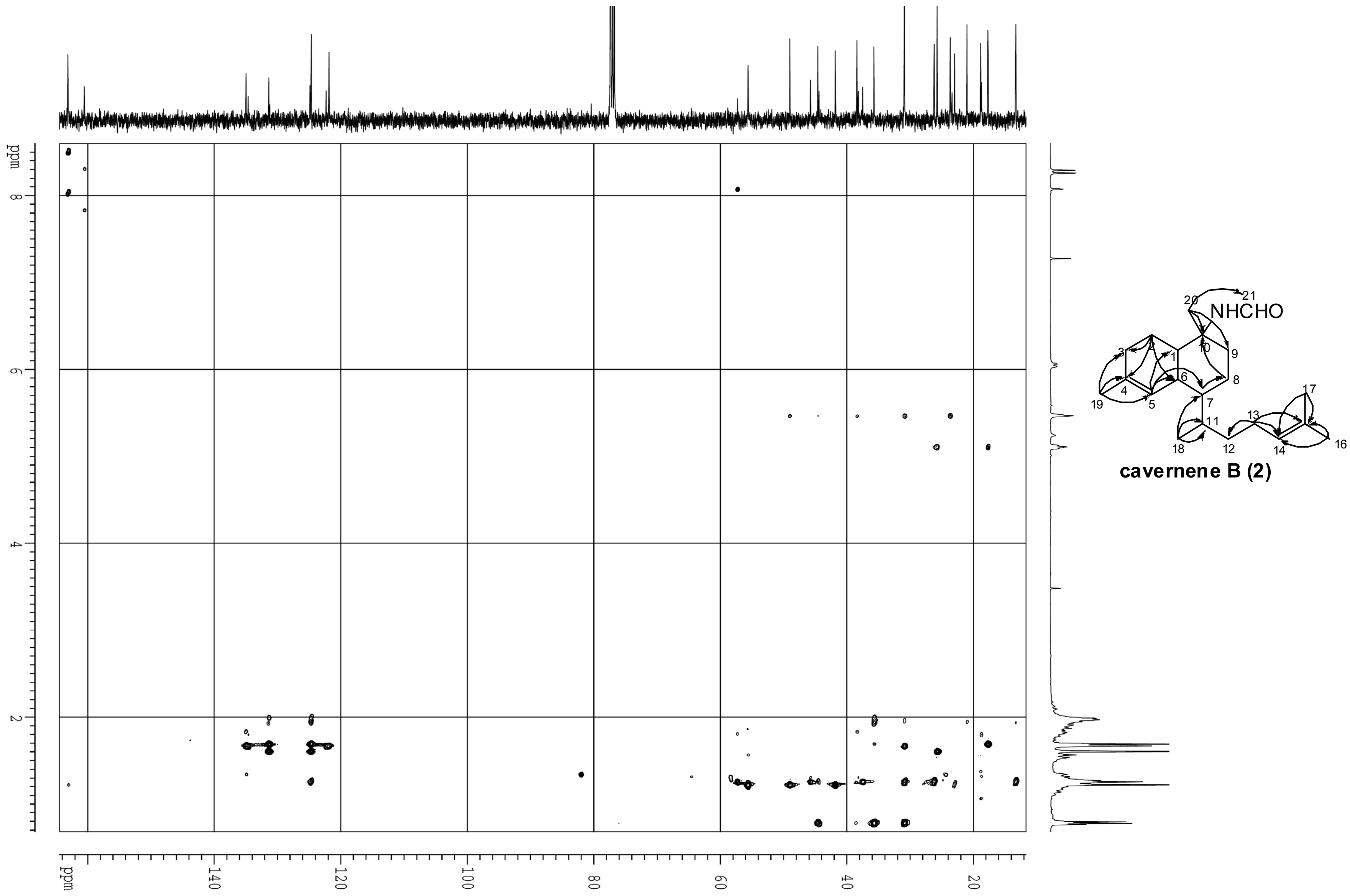

Figure S14. ^{1}H-^{1}H COSY spectrum of cavernene B (**2**) in $CDCl_3$.

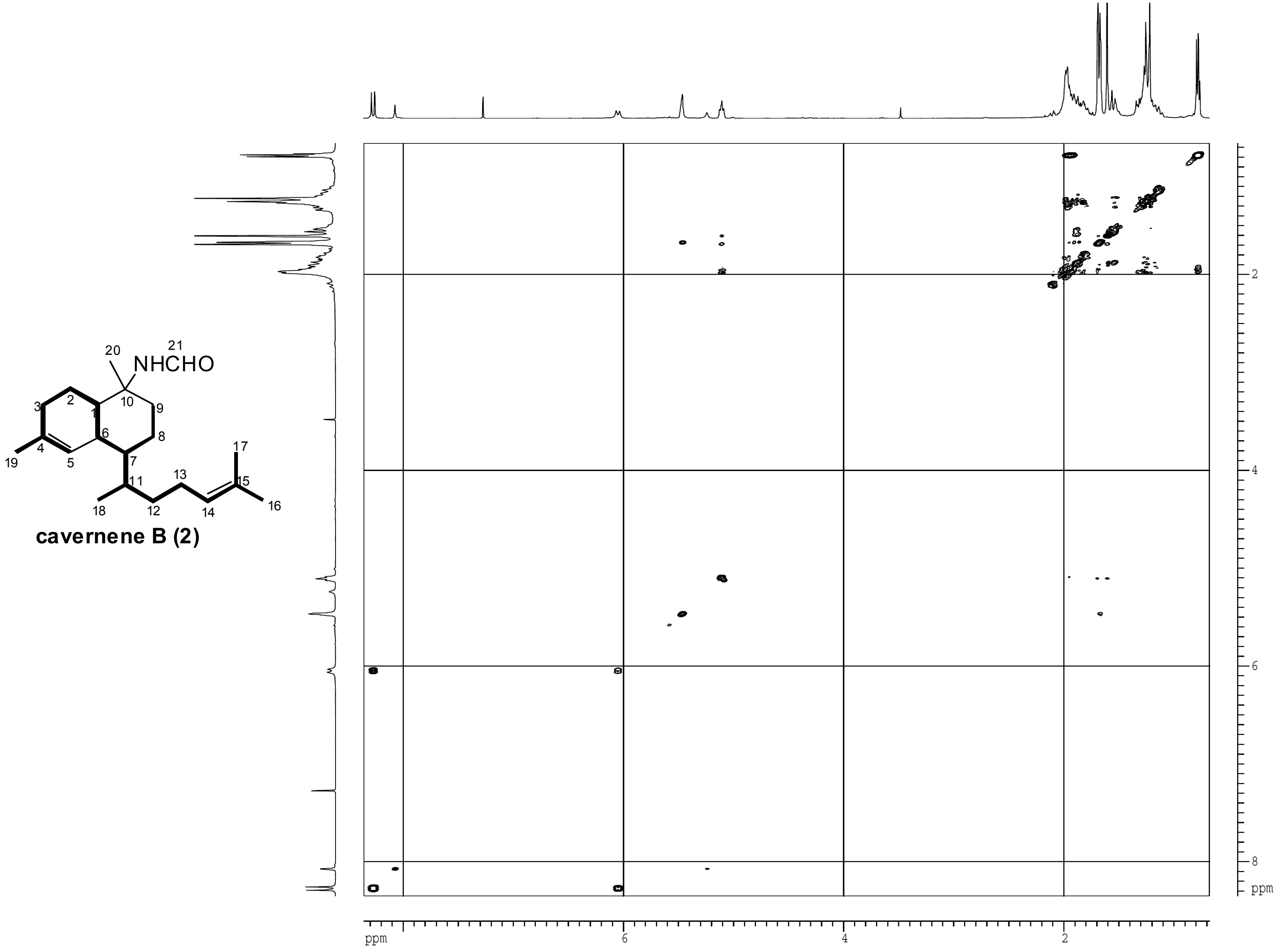

Figure S15. NOESY spectrum of cavernene B (**2**) in $CDCl_3$.

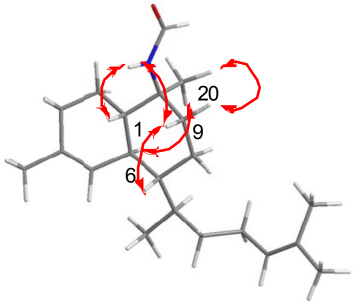

cavernene B (2)

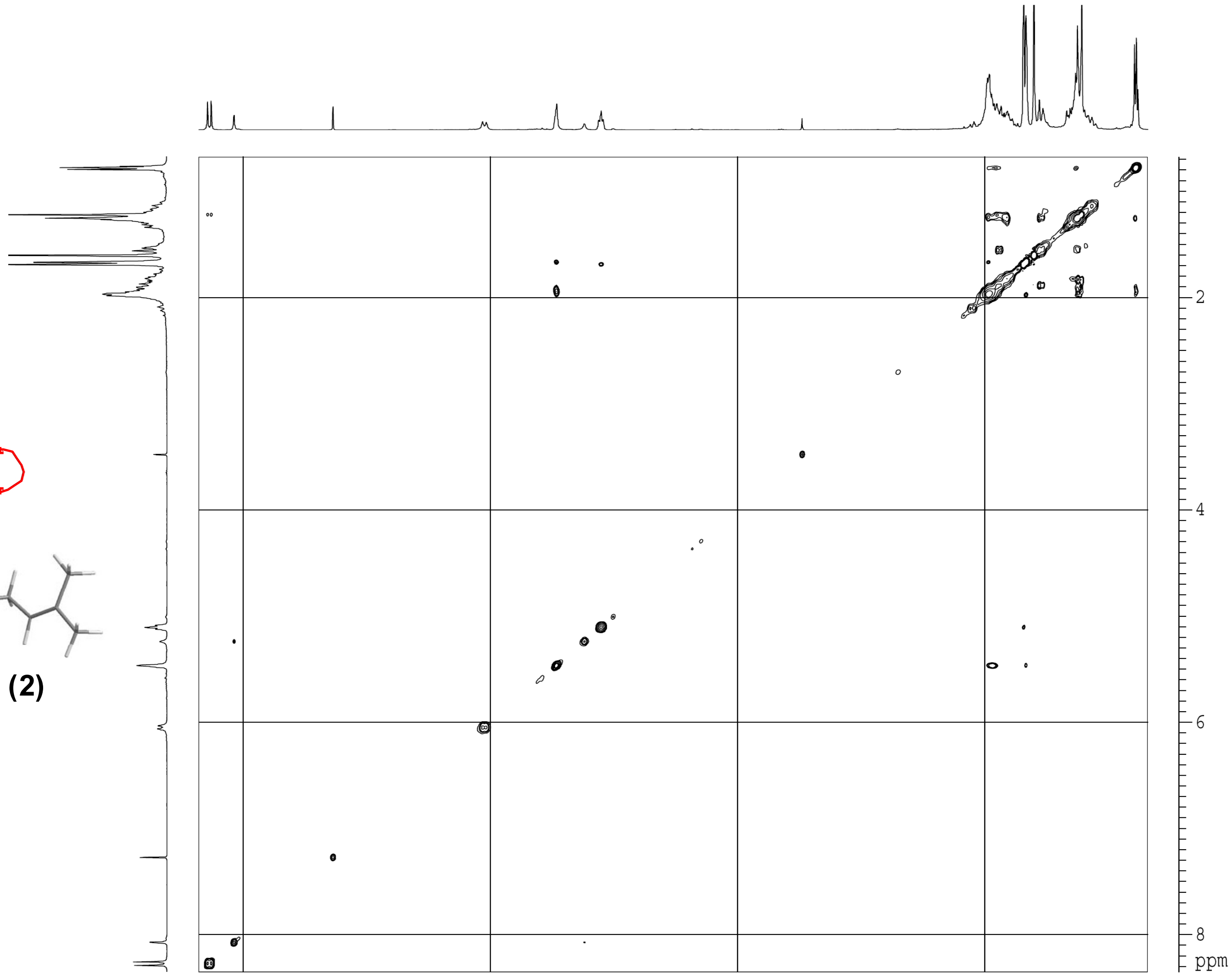

Figure S16. IR spectrum of cavernene B (**2**).

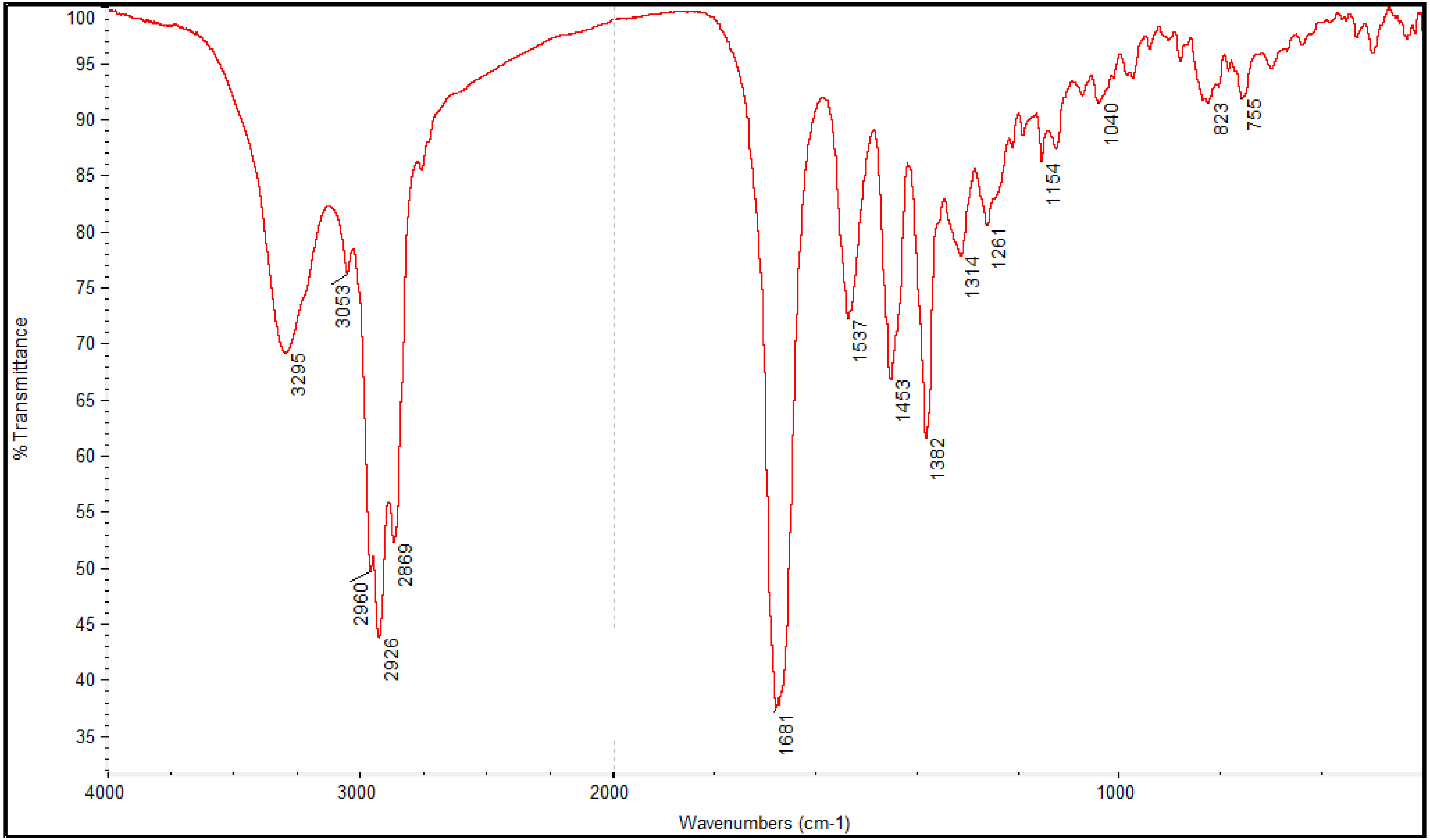

Figure S17. HRESIMS of cavernene B (**2**).

Elemental Composition Report

Tolerance = 10.0 PPM / DBE: min = -1.5, max = 50.0
Selected filters: None

Monoisotopic Mass, Even Electron Ions
9 formula(e) evaluated with 1 results within limits (up to 50 closest results for each mass)
Elements Used:
C: 10-23 H: 5-40 N: 1-1 O: 1-3 Na: 1-1

SIPI Q-Tof micro YA019 19-Mar-2011,14:41:28 0.00000000
WQ11-148H 33 (1.141) AM (Cen,6, 80.00, Ar,5000.0,342.15,0.70); Sm (SG, 2x3.00); Cm (21:33) TOF MS ES+ 9.27e3

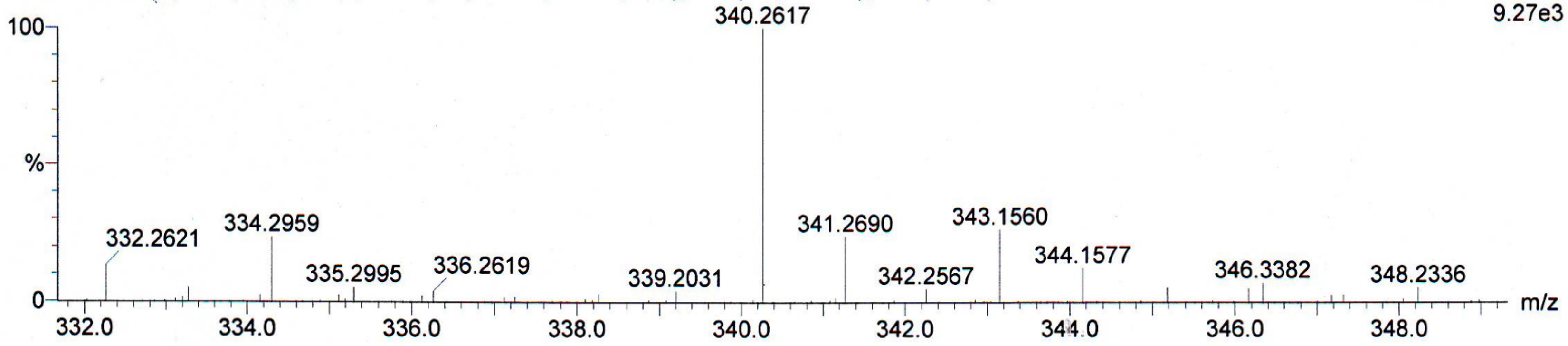

Minimum:	30.00				-1.5		
Maximum:	100.00		5.0	10.0	50.0		
Mass	RA	Calc. Mass	mDa	PPM	DBE	i-FIT	Formula
340.2617	100.00	340.2616	0.1	0.3	4.5	27.4	C21 H35 N O Na

Figure S18. UV spectrum of cavernene B (**2**).

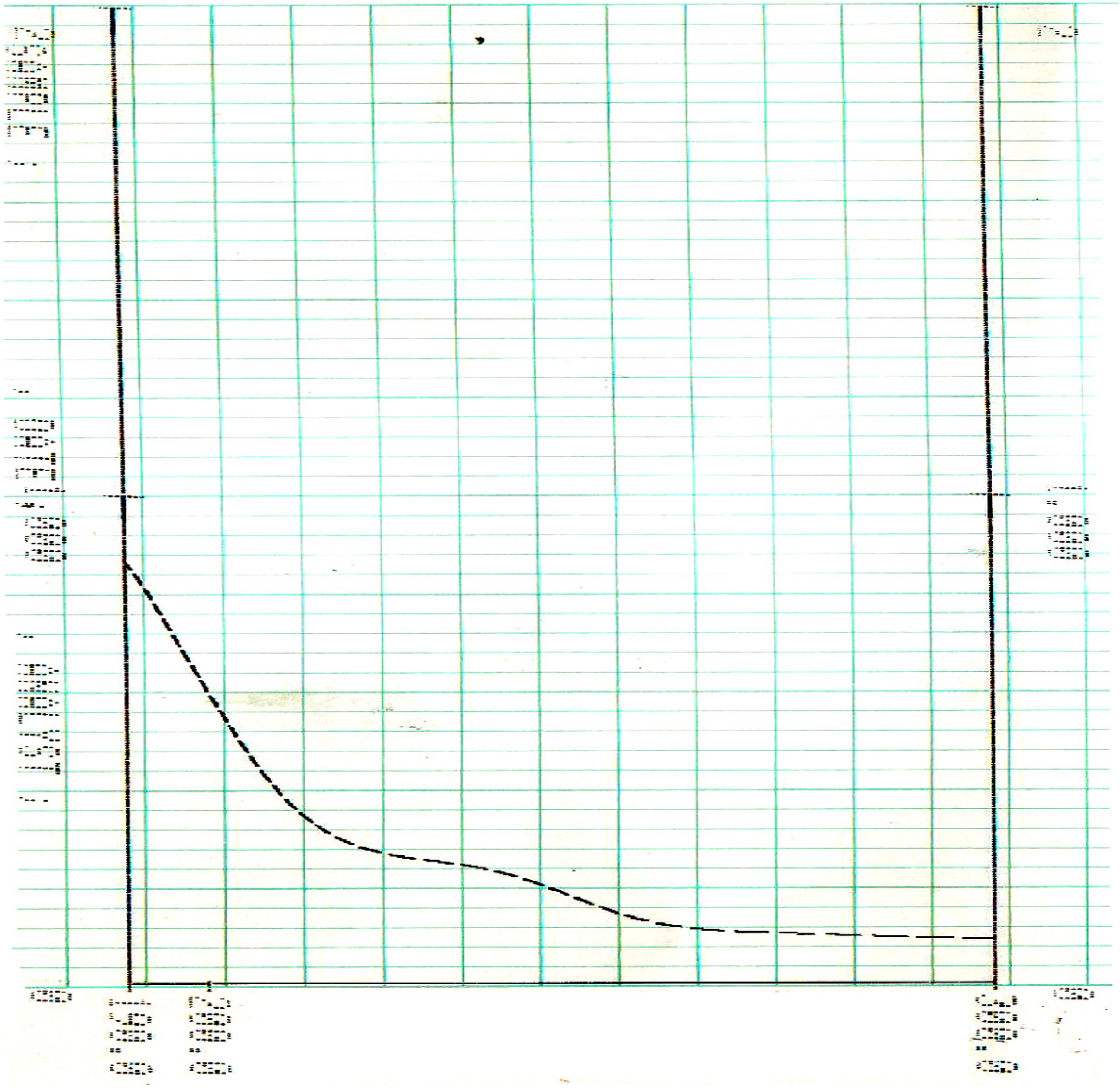

Figure S19. ^{1}H NMR spectrum of cavernene C (**3**) in $CDCl_3$.

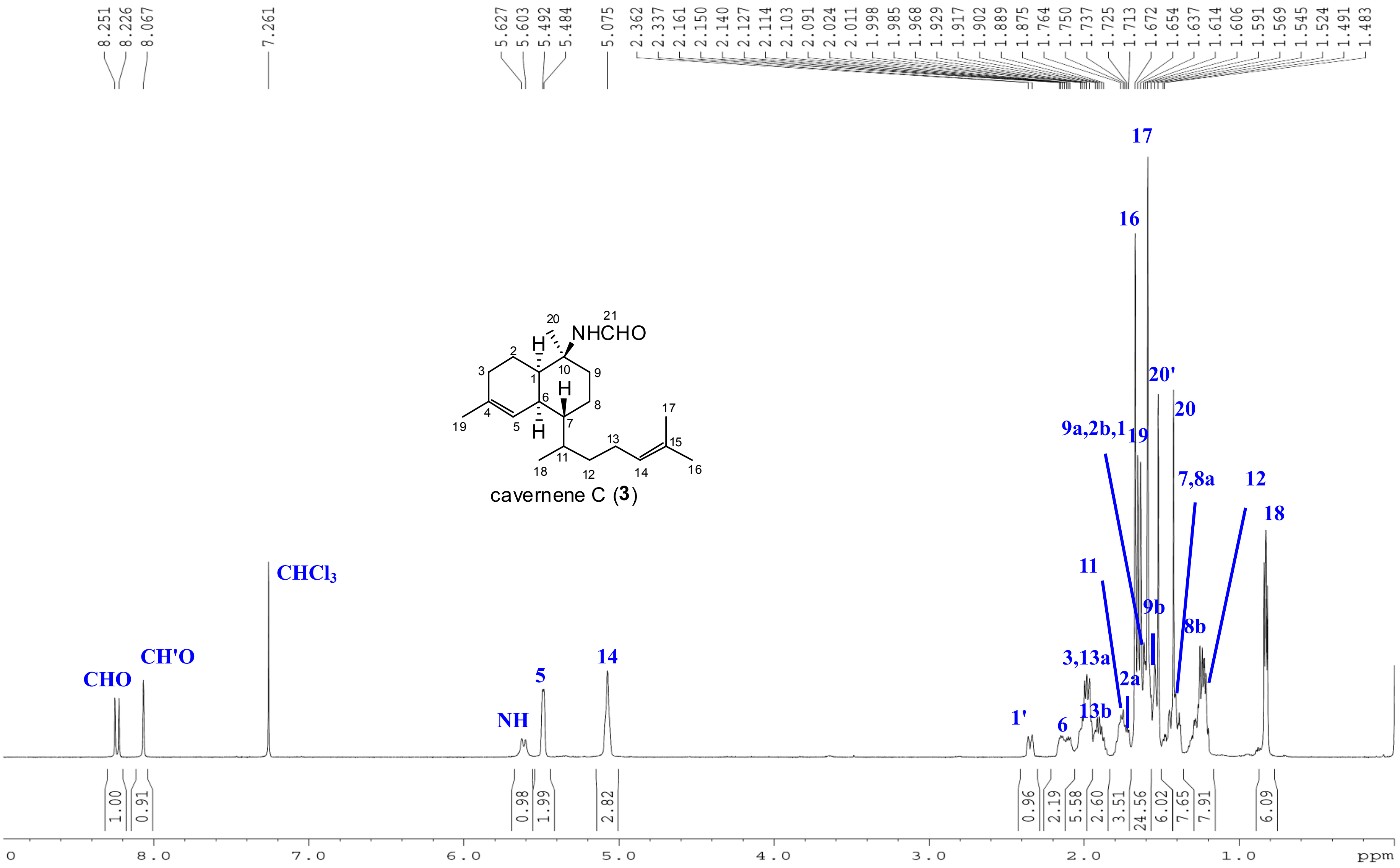

Figure S20. ^{13}C NMR spectrum of cavernene C (**3**) in $CDCl_3$.

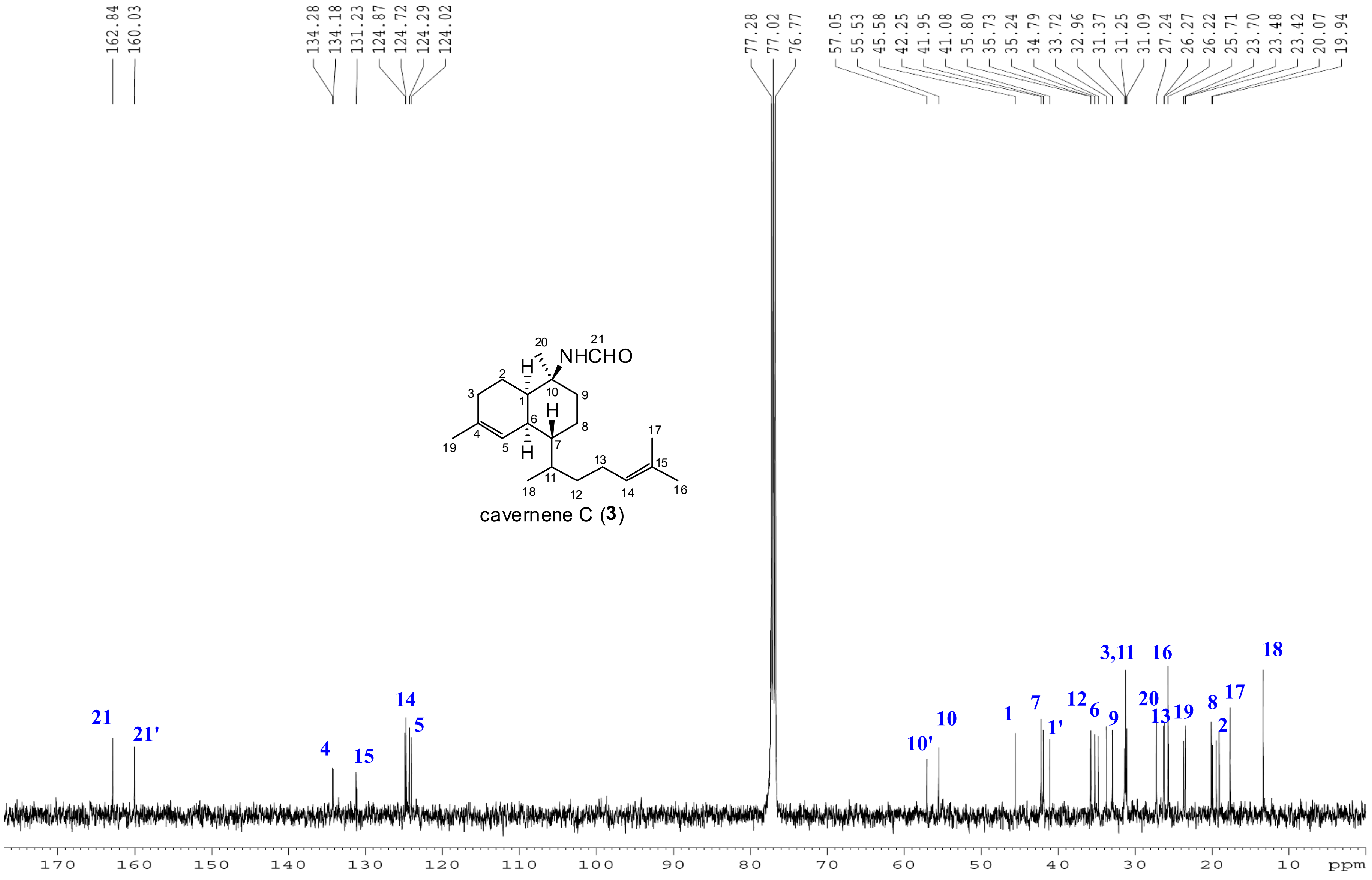

Figure S21. HSQC spectrum of cavernene C (**3**) in $CDCl_3$.

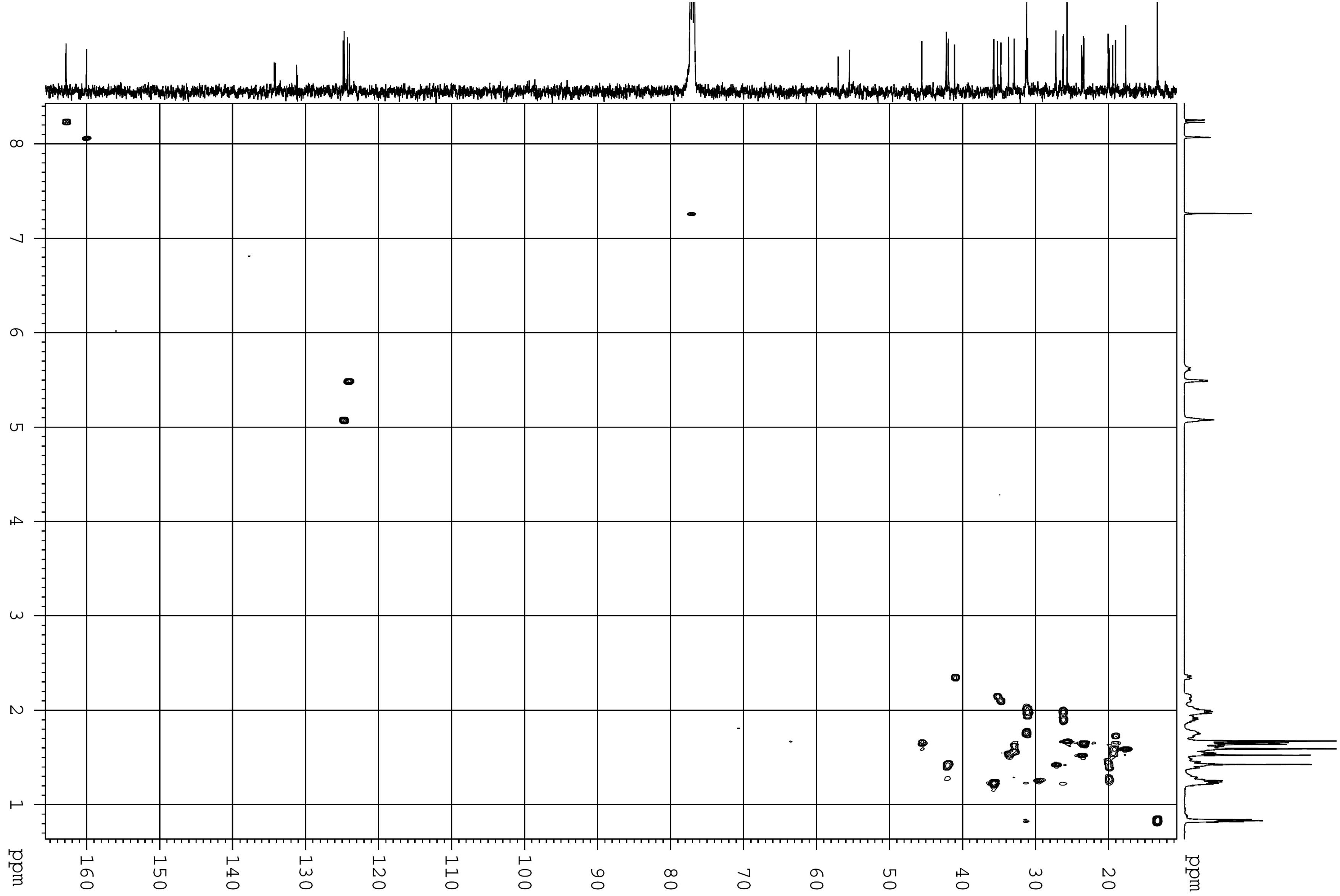

Figure S22. HMBC spectrum of cavernene C (**3**) in $CDCl_3$.

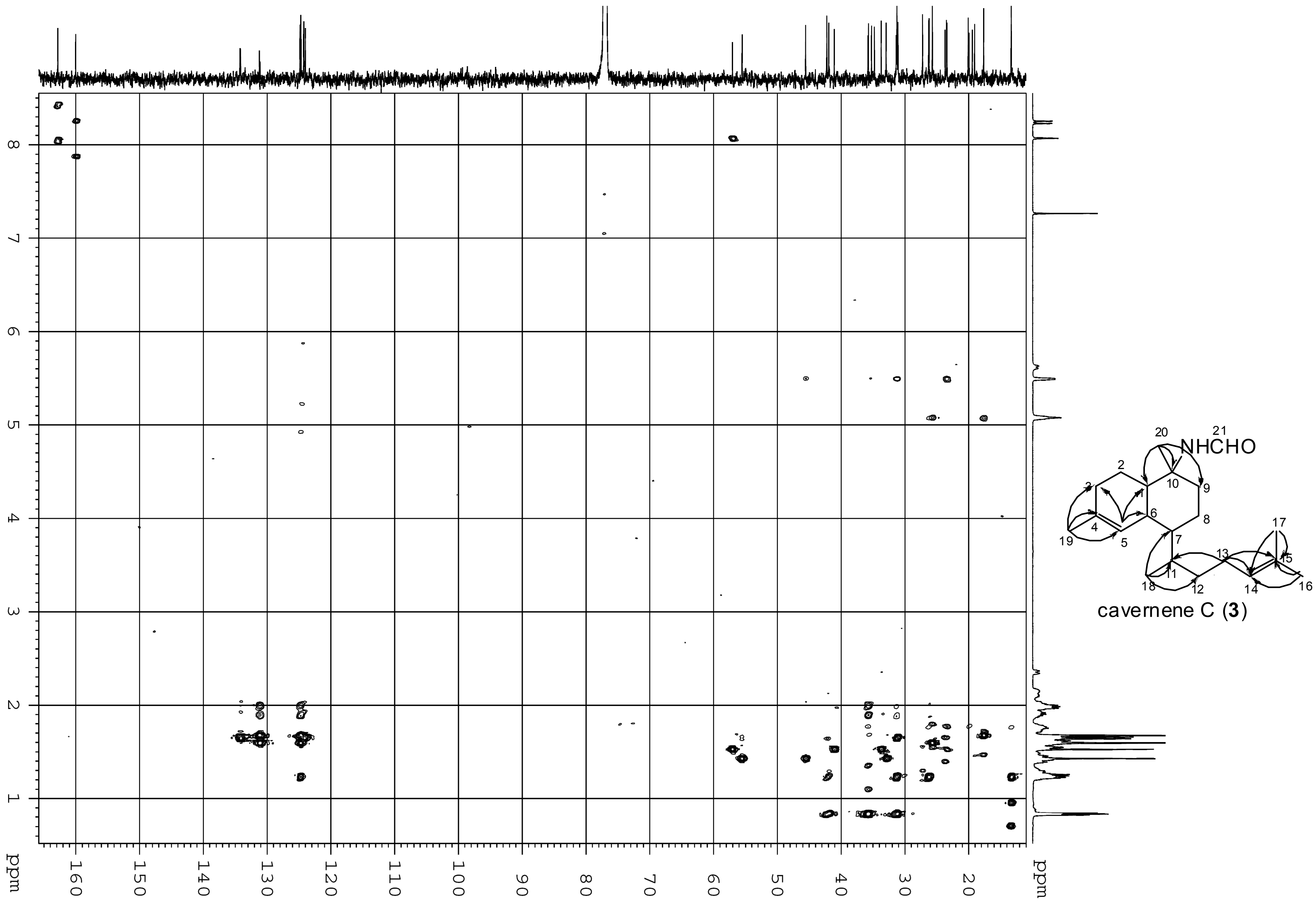

Figure S23. ^{1}H-^{1}H COSY spectrum of cavernene C (**3**) in $CDCl_3$.

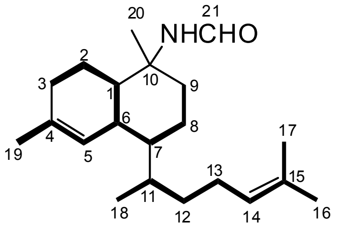

cavernene C (**3**)

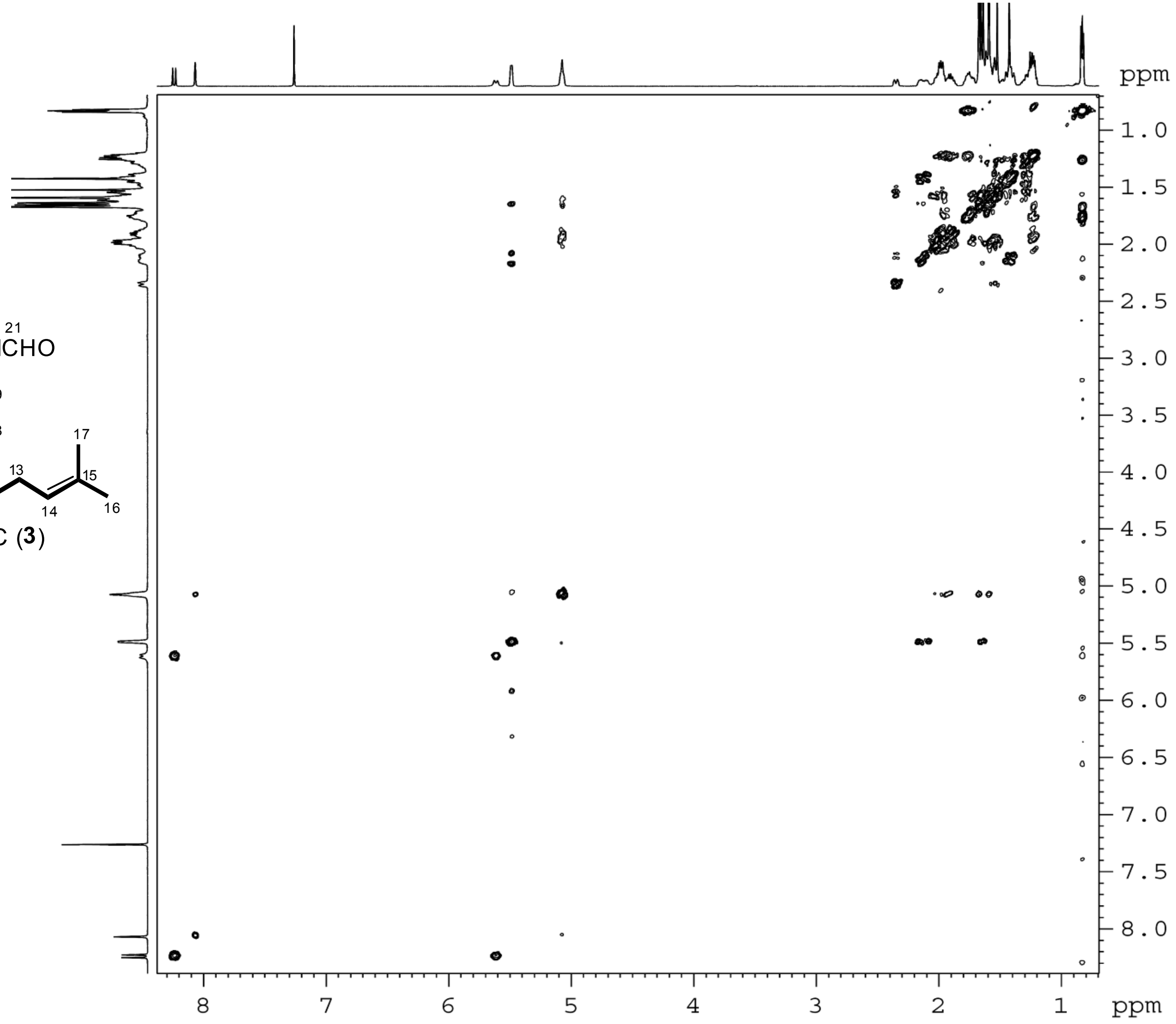

Figure S24. NOESY spectrum of cavernene C (**3**) in $CDCl_3$.

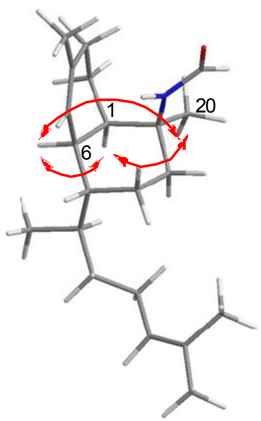

cavernene C (**3**)

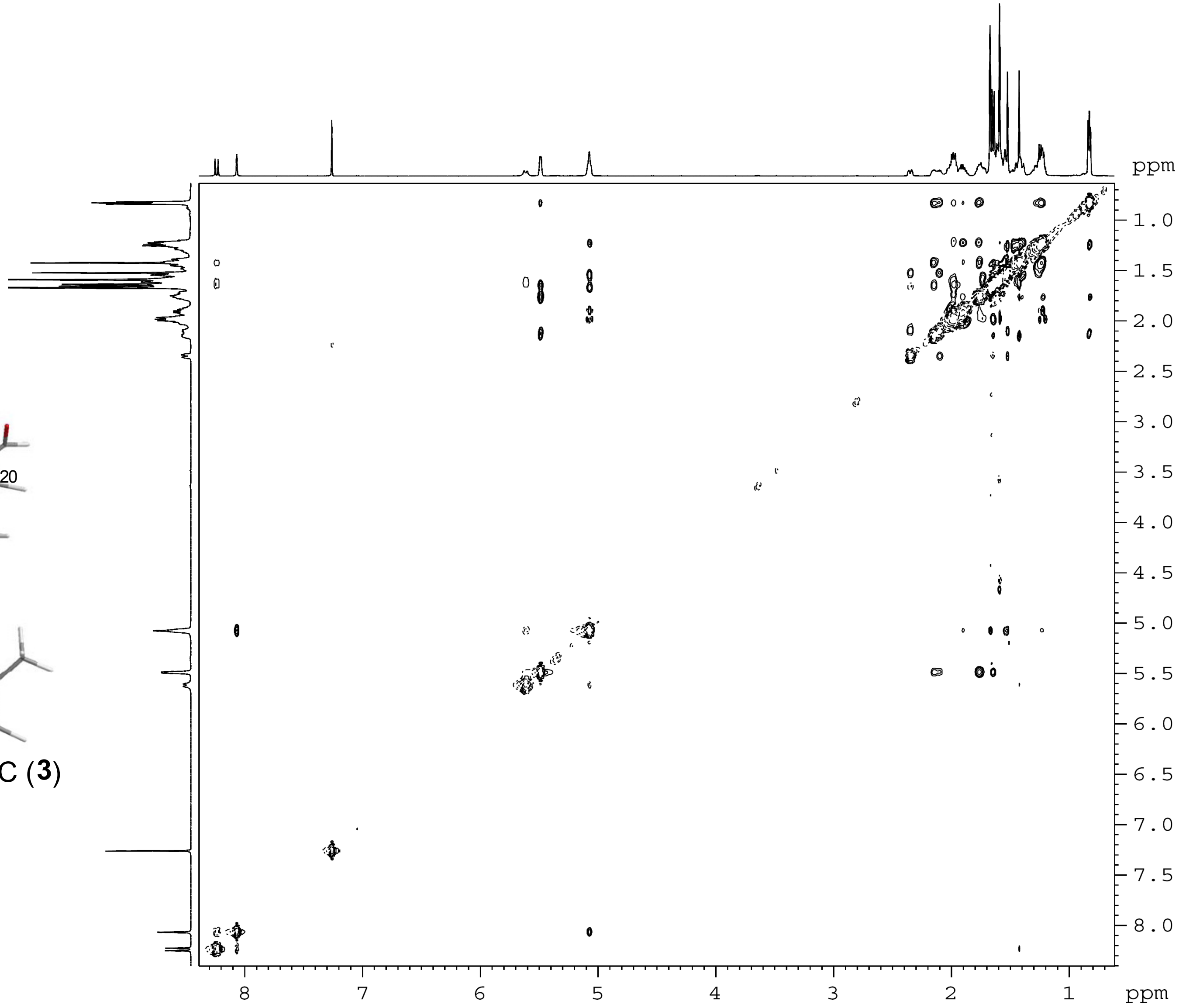

Figure S25. IR spectrum of cavernene C (**3**).

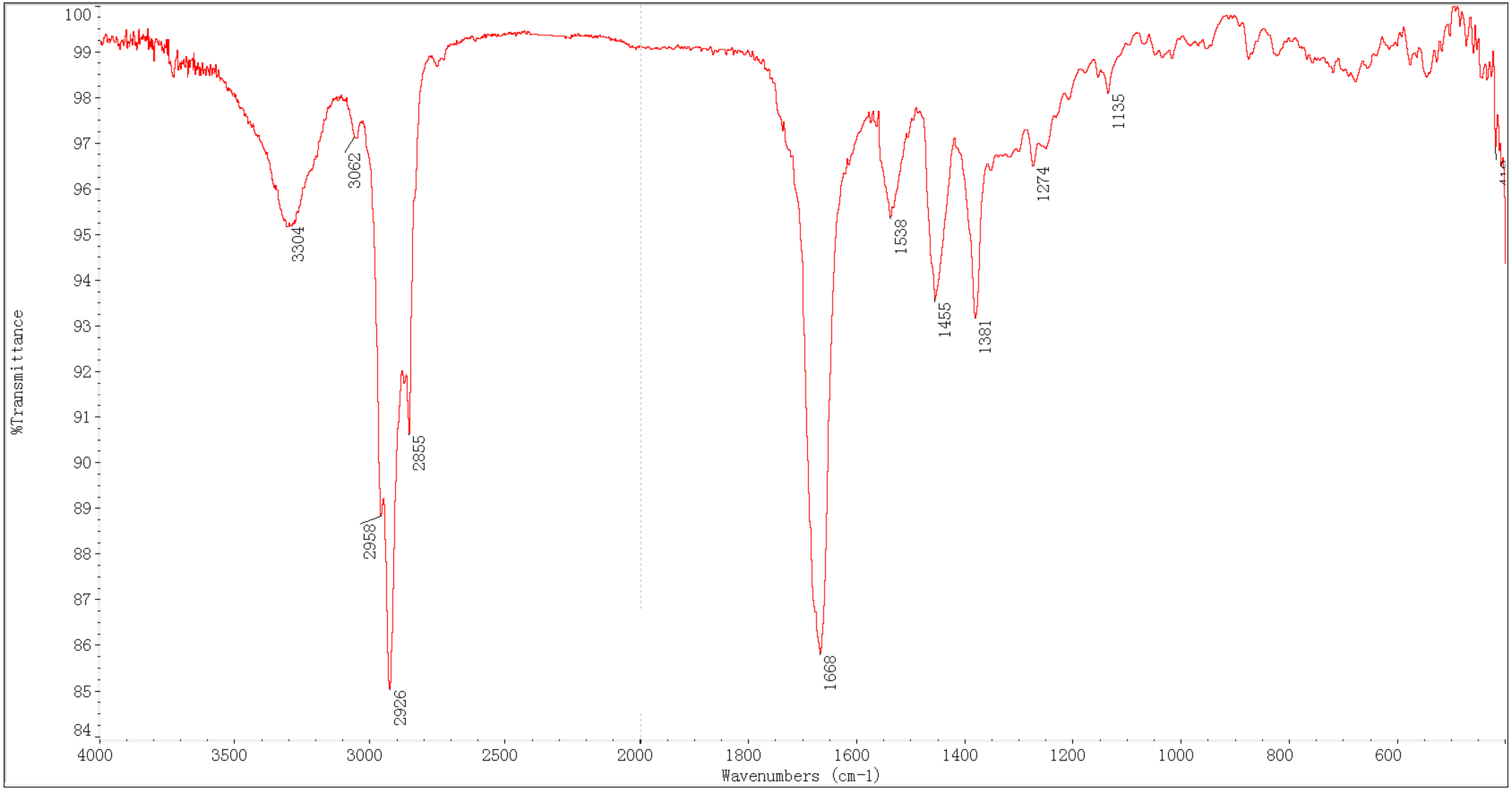

Figure S26. HRESIMS of cavernene C (**3**).

Elemental Composition Report

Tolerance = 50.0 PPM / DBE: min = -1.5, max = 50.0
Selected filters: None

Monoisotopic Mass, Even Electron Ions
9 formula(e) evaluated with 1 results within limits (up to 50 closest results for each mass)
Elements Used:
C: 10-25 H: 5-40 N: 1-1 O: 1-3 Na: 1-1

SIPI Q-Tof micro YA019 09-Apr-2011,14:55:03

WQ11-233H 32 (1.126) AM (Med,6, Ar,5000.0,354.24,0.70); Sm (SG, 2x3.00); Cm (20:32) 0.00000000 TOF MS ES+ 5.67e4

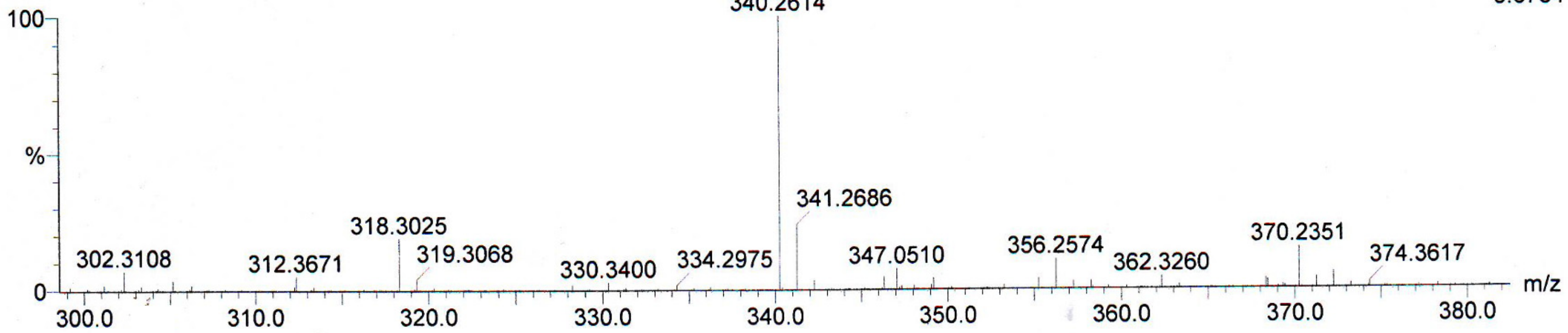

Minimum:	30.00				-1.5		
Maximum:	100.00		5.0	50.0	50.0		

Mass	RA	Calc. Mass	mDa	PPM	DBE	i-FIT	Formula
340.2614	100.00	340.2616	-0.2	-0.6	4.5	25.4	C21 H35 N O Na

Figure S27. UV spectrum of cavernene C (**3**).

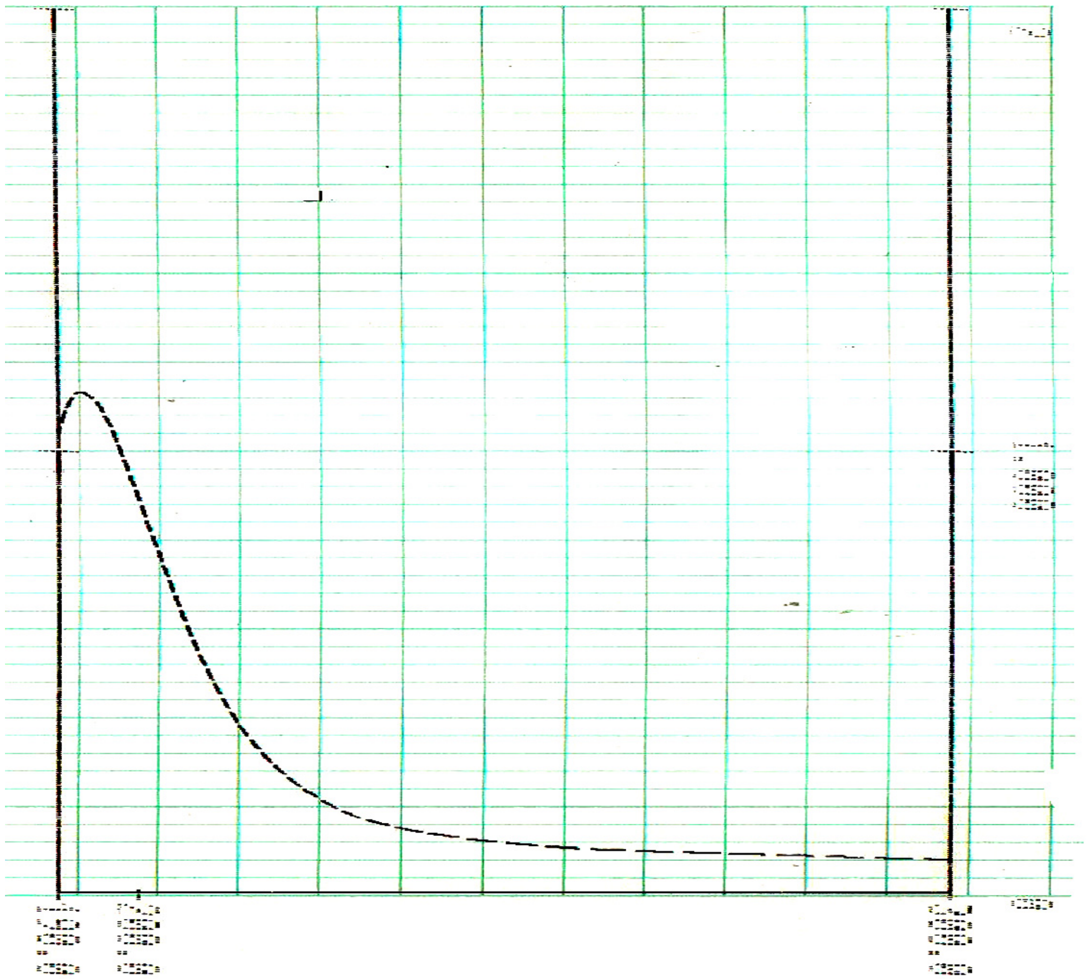

Figure S28. ^{1}H NMR spectrum of cavernene D (**4**) in $CDCl_3$.

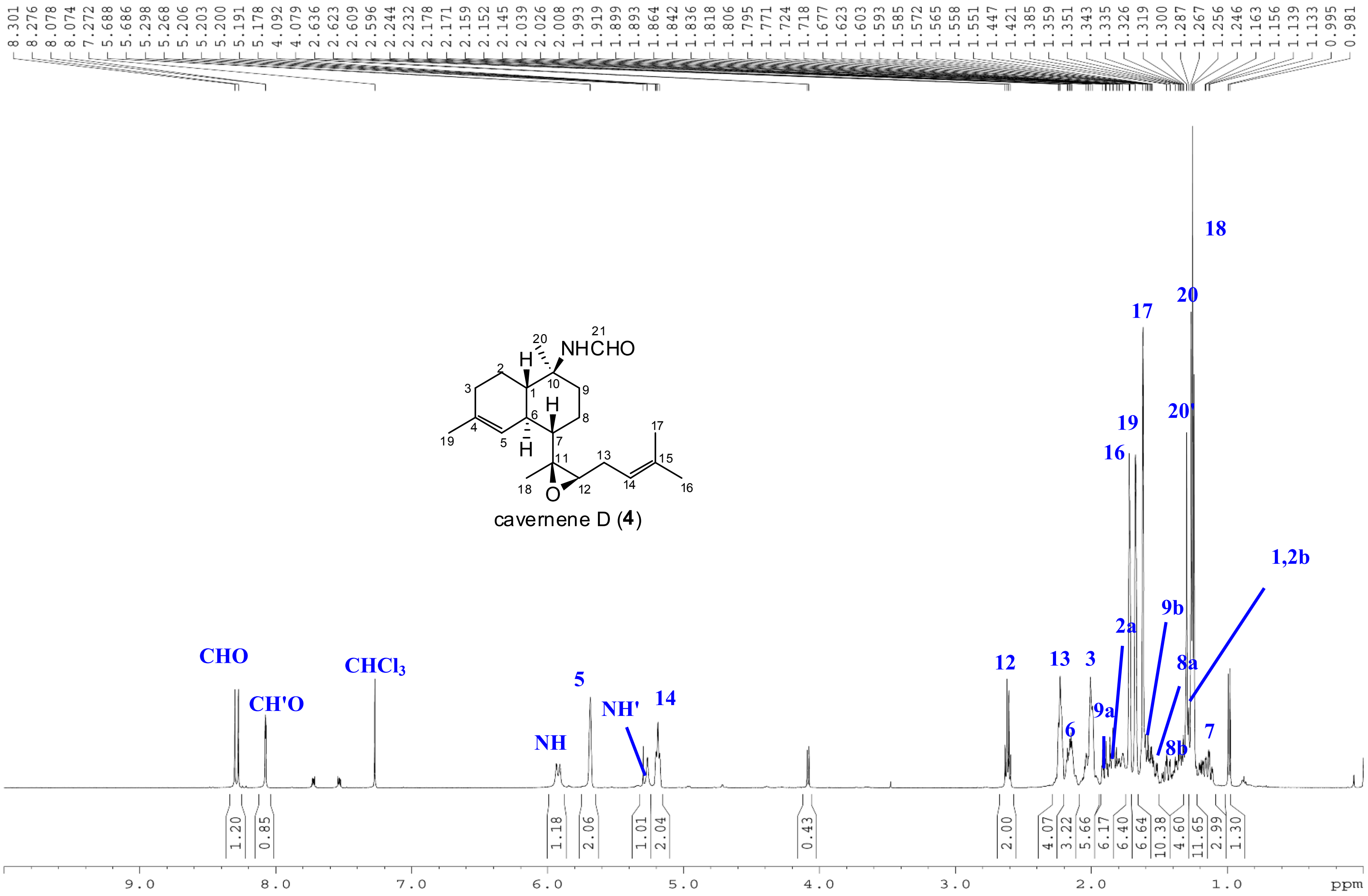

Figure S29. ^{13}C NMR spectrum of cavernene D (**4**) in $CDCl_3$.

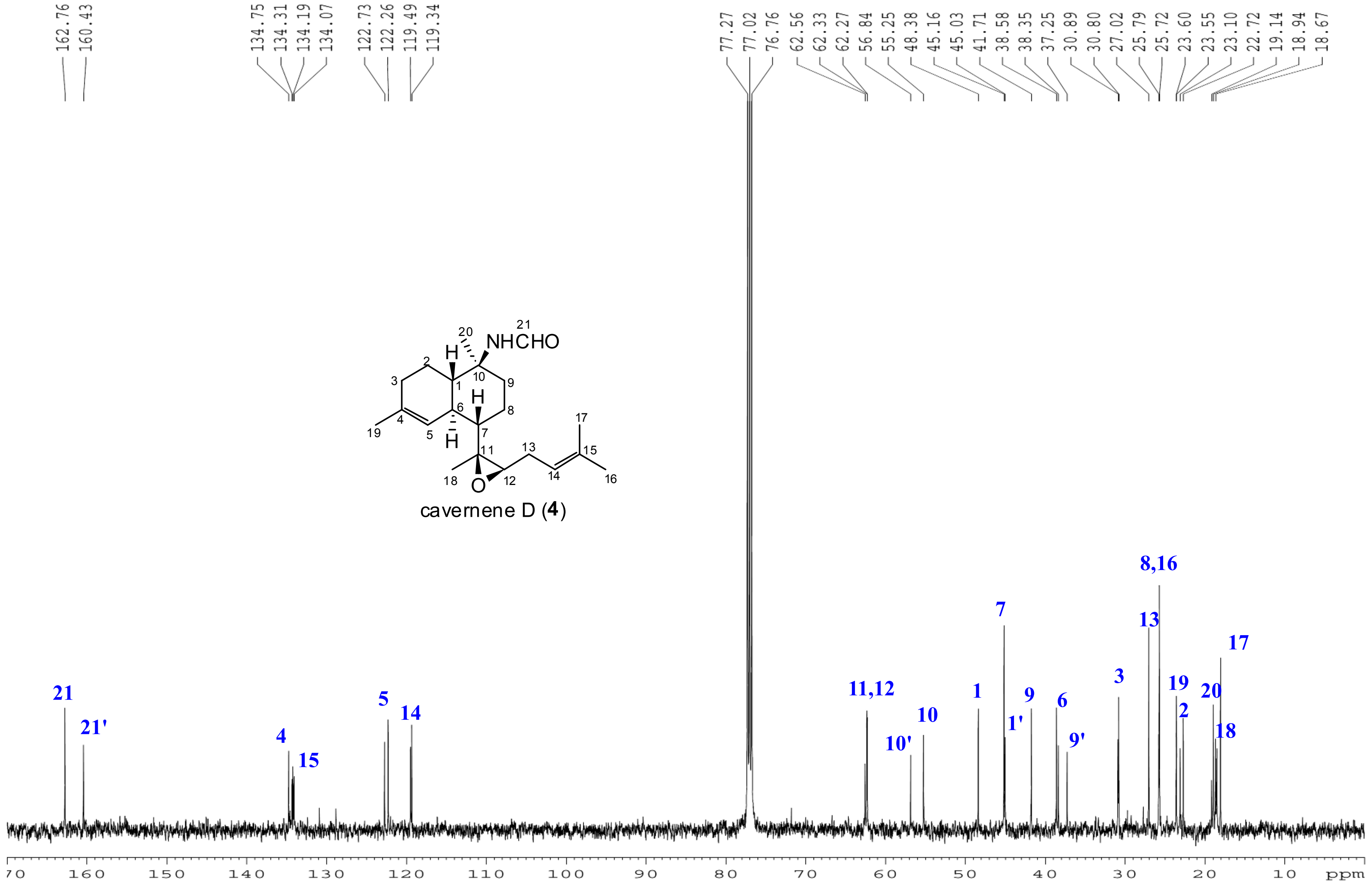

Figure S30. HSQC spectrum of cavernene D (**4**) in $CDCl_3$.

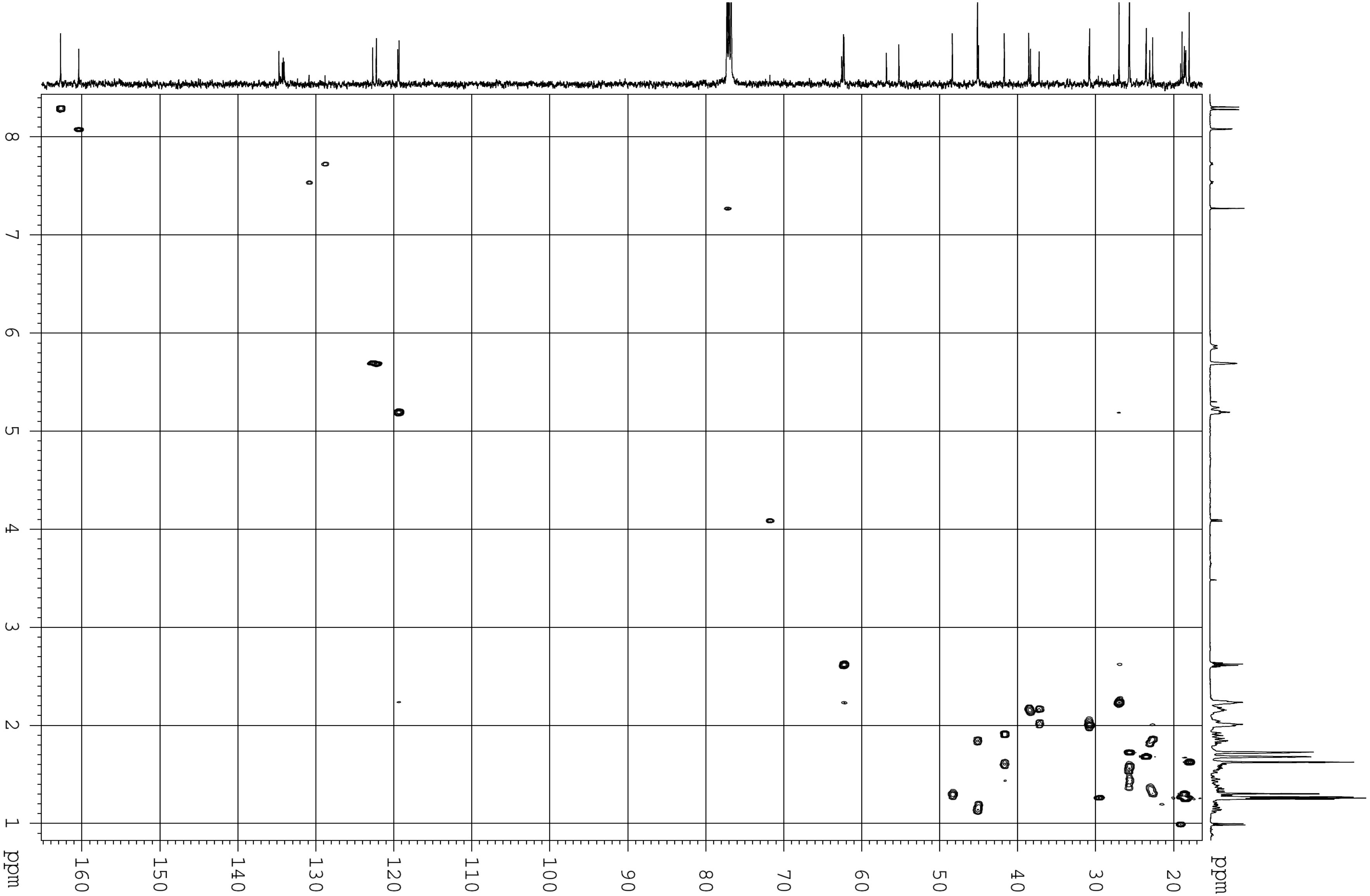

Figure S31. HMBC spectrum of cavernene D (**4**) in $CDCl_3$.

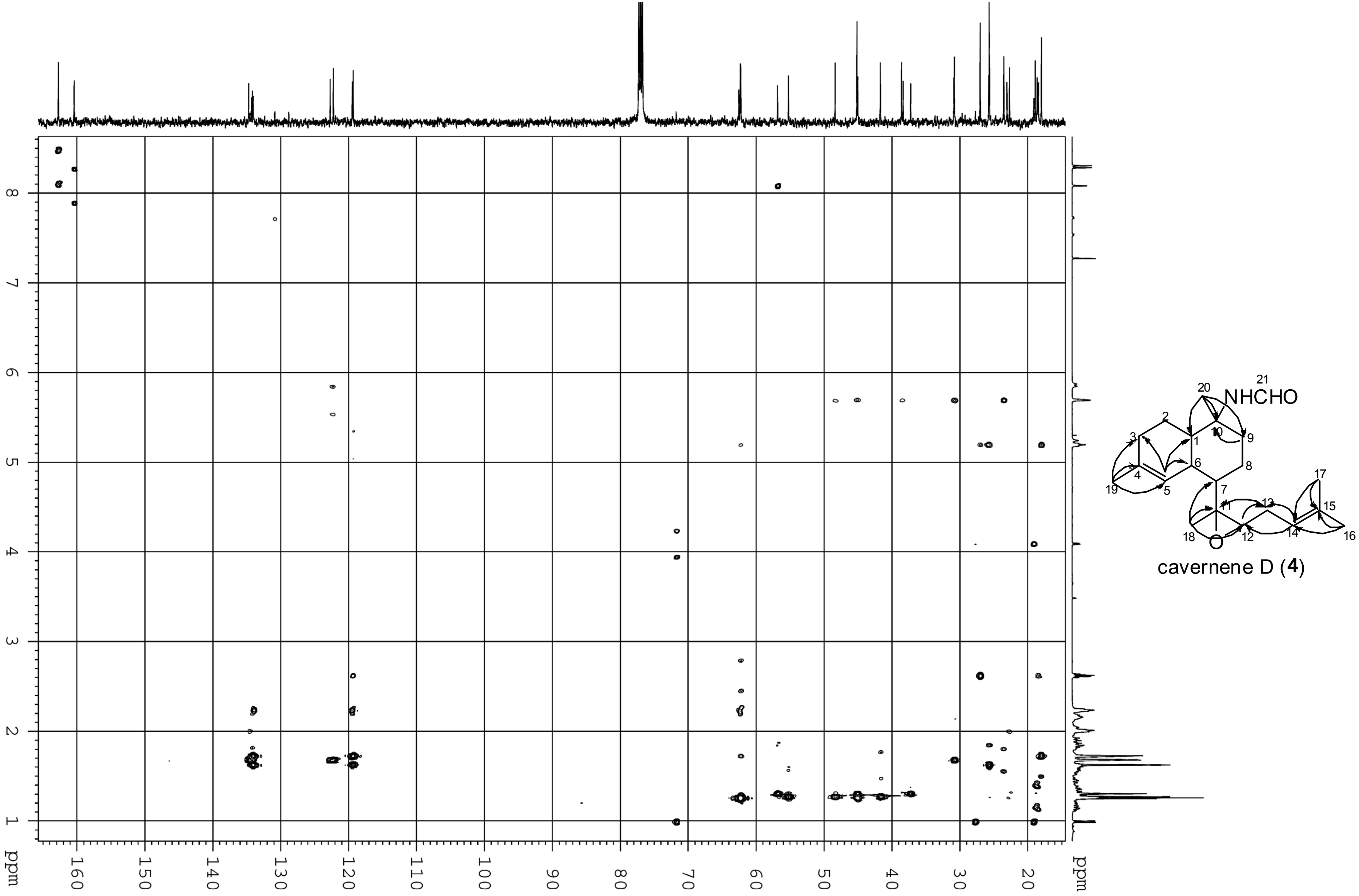

Figure S32. ¹H-¹H COSY spectrum of cavernene D (**4**) in $CDCl_3$.

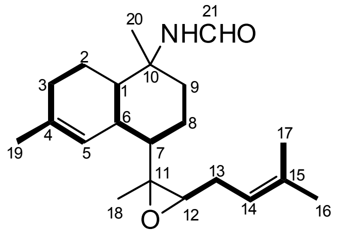

cavernene D (**4**)

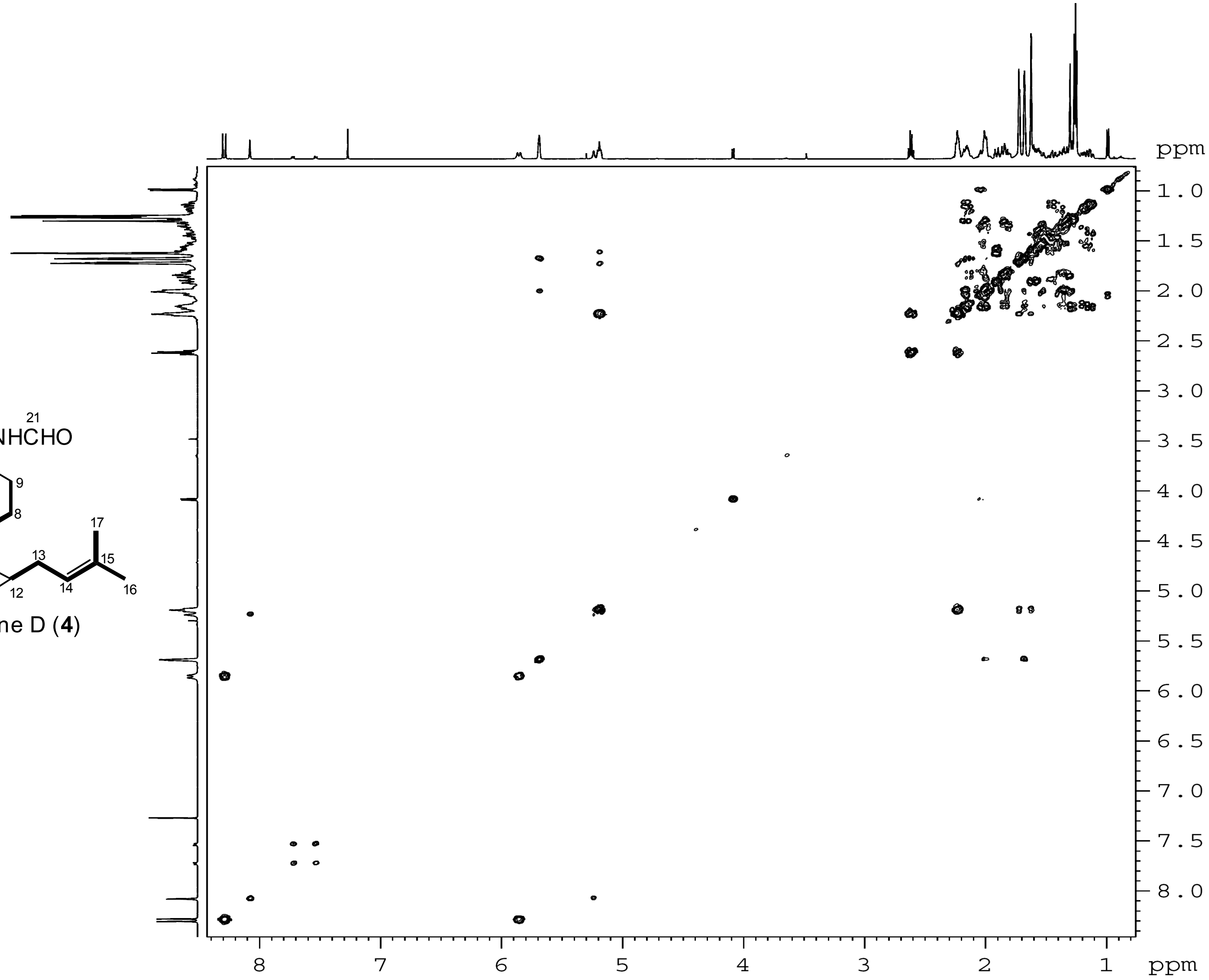

Figure S33. NOESY spectrum of cavernene D (**4**) in $CDCl_3$.

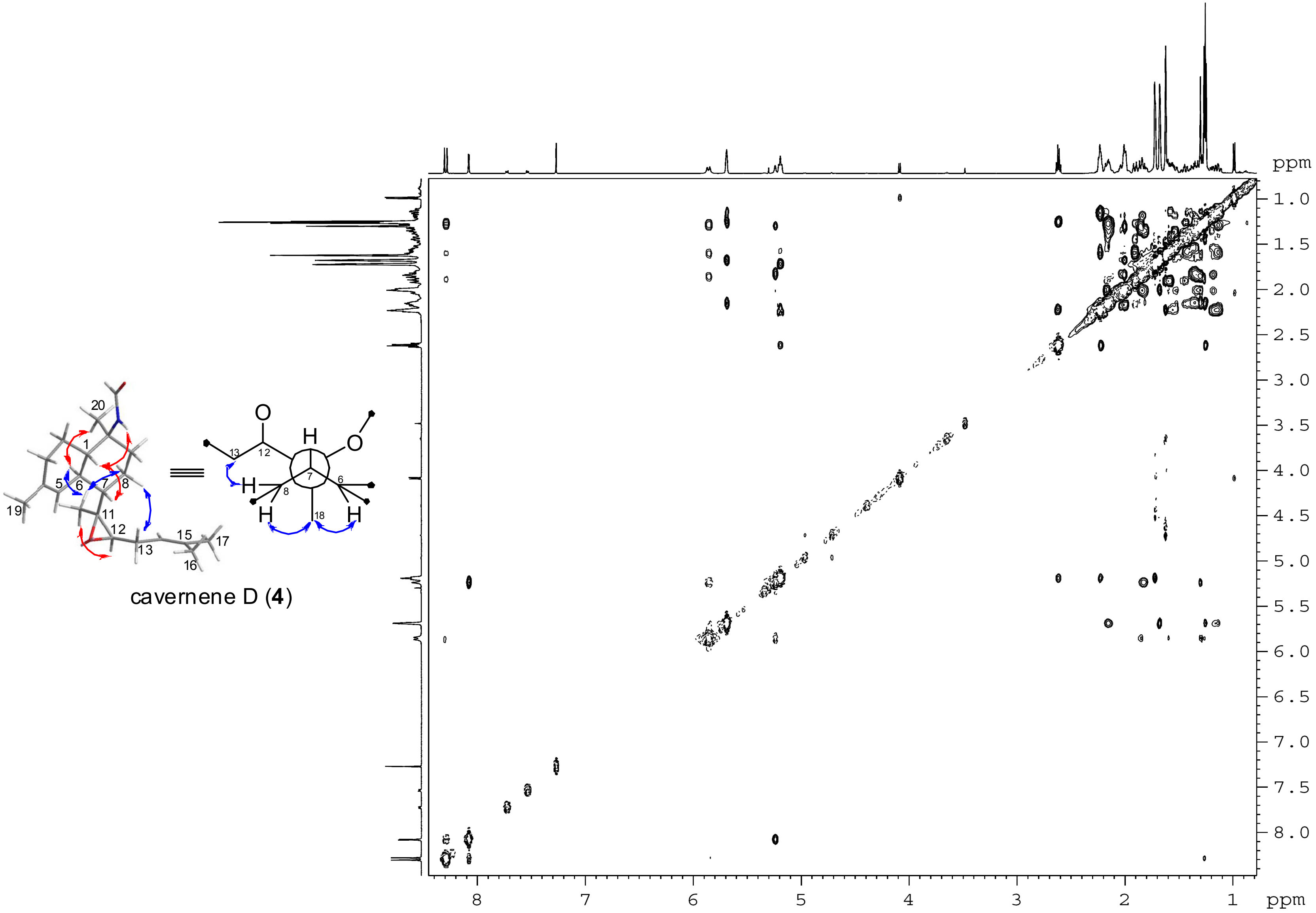

Figure S34. IR spectrum of cavernene D (**4**).

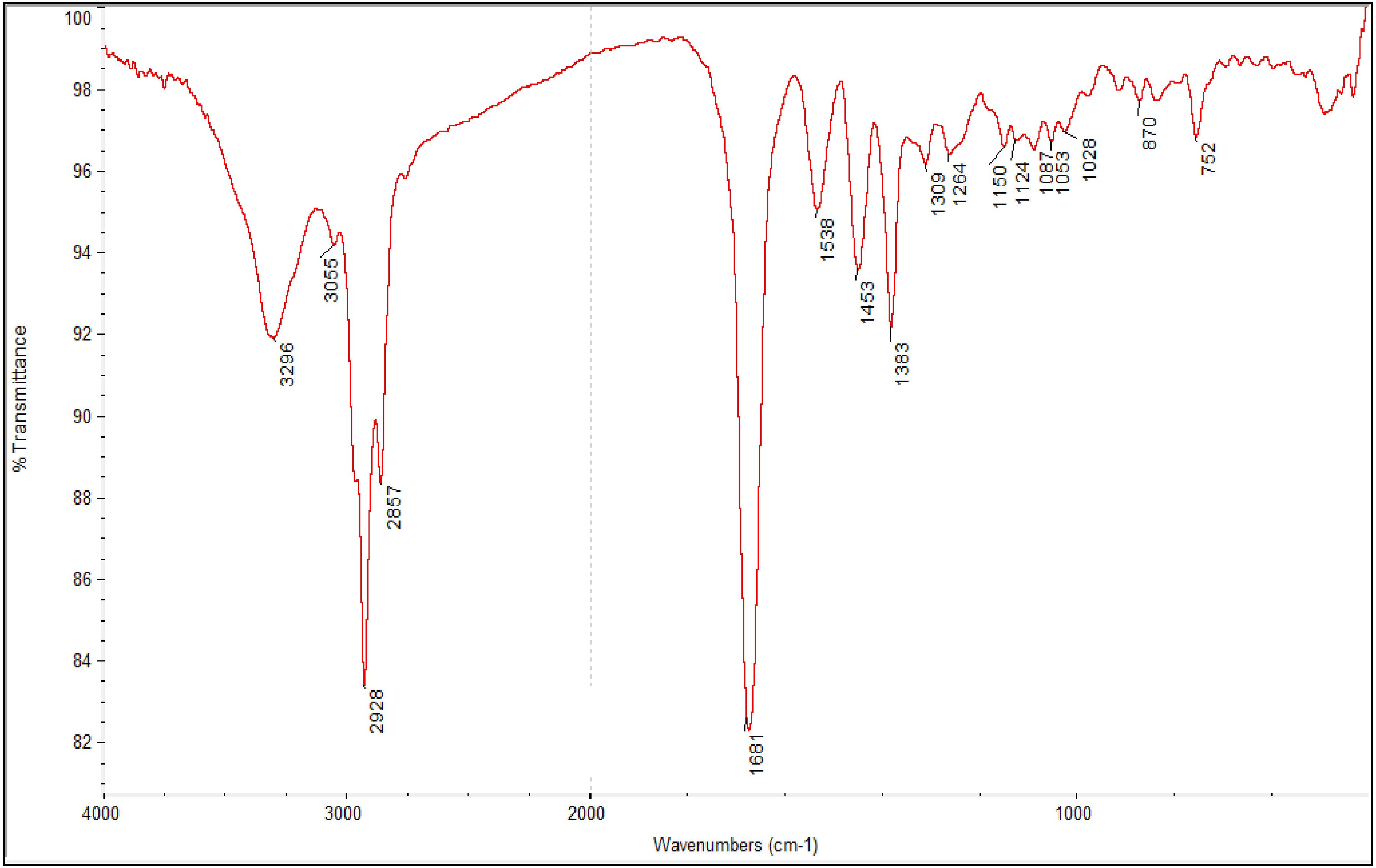

Figure S35. HRESIMS of cavernene D (**4**).

Elemental Composition Report

Tolerance = 10.0 PPM / DBE: min = -1.5, max = 50.0
Selected filters: None

Monoisotopic Mass, Even Electron Ions
7 formula(e) evaluated with 1 results within limits (up to 50 closest results for each mass)
Elements Used:
C: 10-23 H: 5-36 N: 1-1 O: 1-3 Na: 1-1

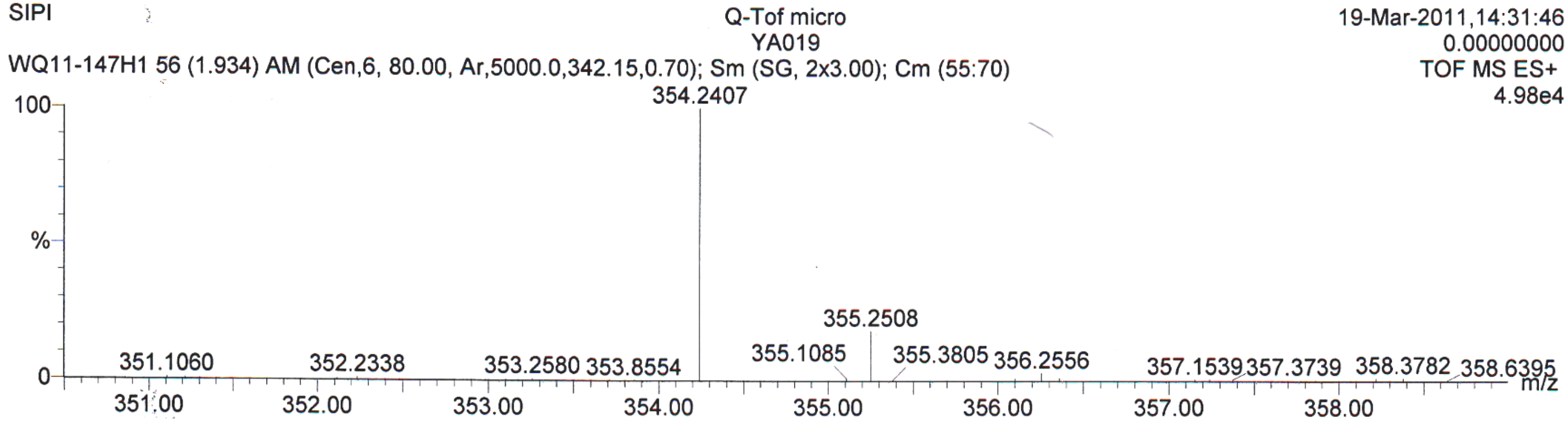

Minimum:	30.00				-1.5		
Maximum:	100.00		5.0	10.0	50.0		
Mass	RA	Calc. Mass	mDa	PPM	DBE	i-FIT	Formula
354.2407	100.00	354.2409	-0.2	-0.6	5.5	424.7	C21 H33 N O2 Na

Figure S36. UV spectrum of cavernene D (**4**).

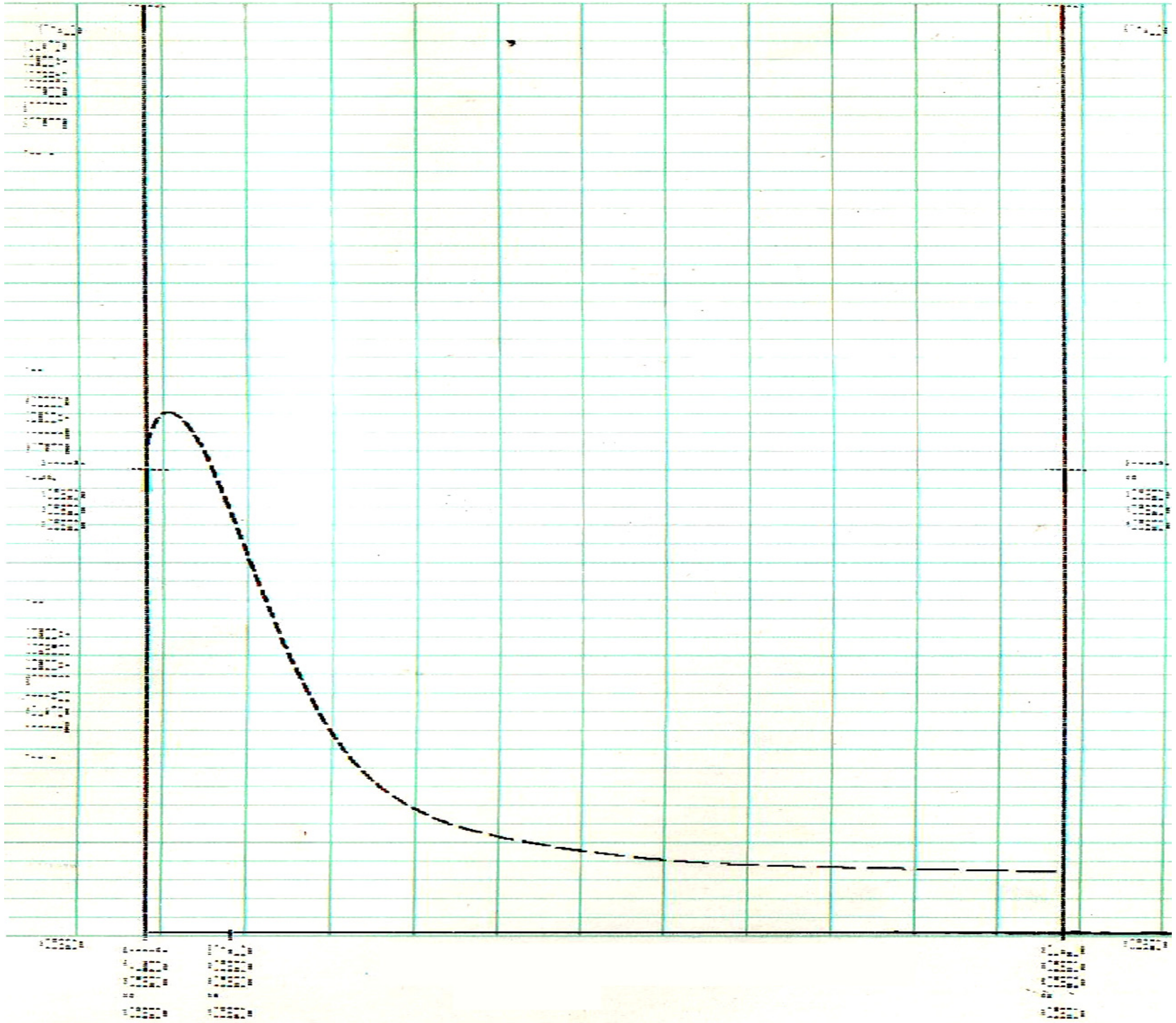

Figure S37. ^{1}H NMR spectrum of kalihinene E (**5**) in $CDCl_3$.

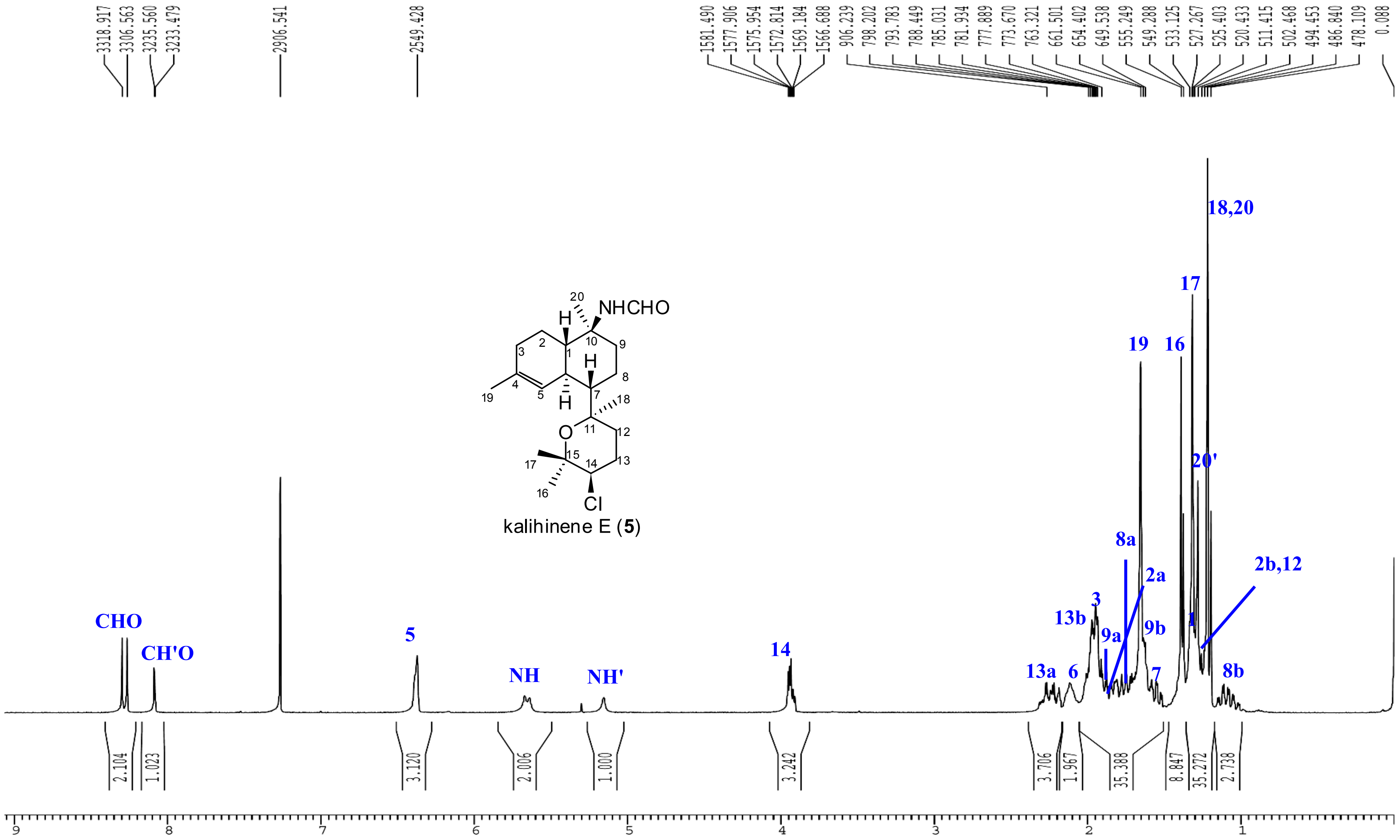

Figure S38. ^{13}C NMR spectrum of kalihinene E (**5**) in $CDCl_3$.

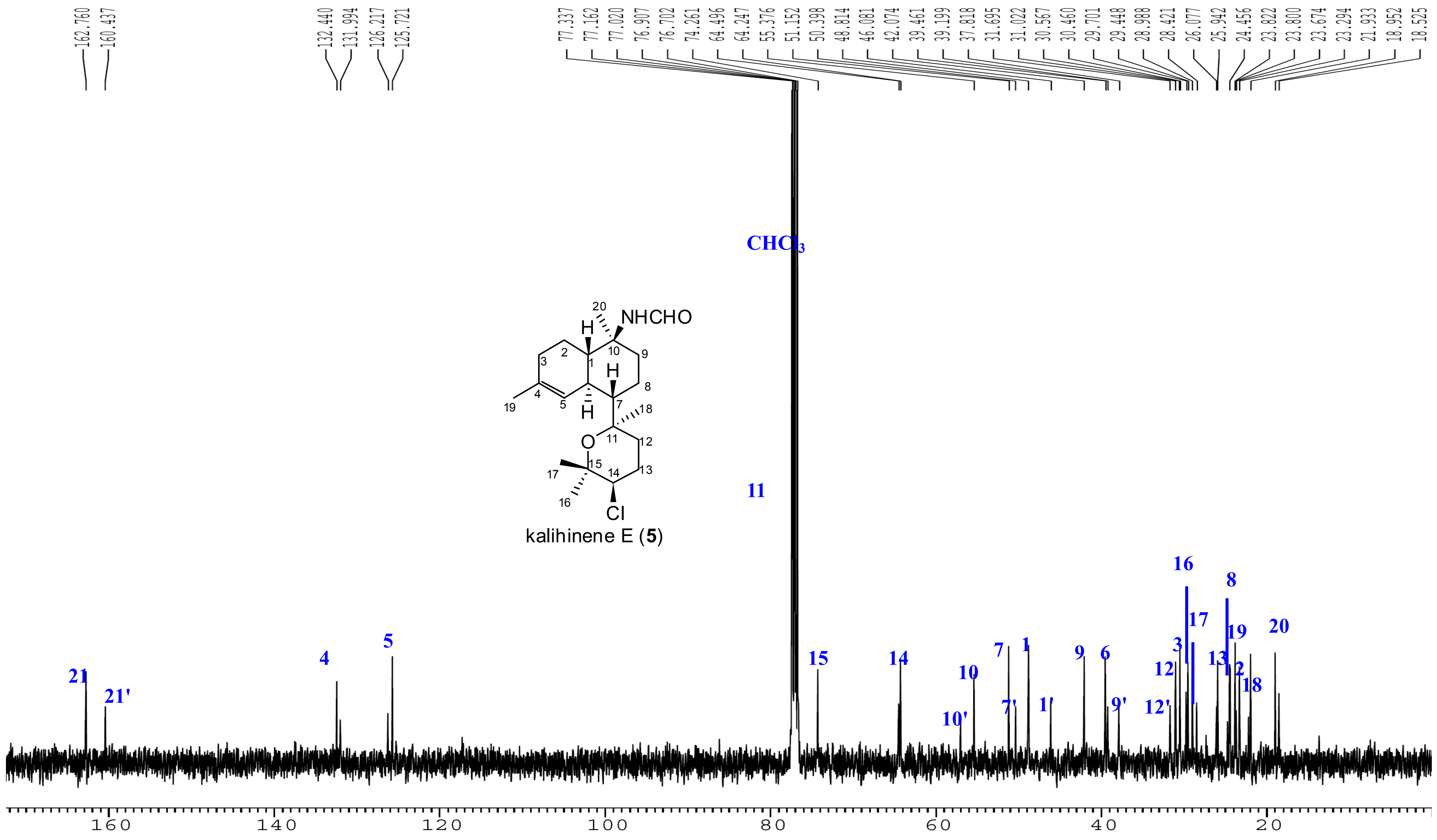

Figure S39. HSQC spectrum of kalihinene E (**5**) in $CDCl_3$.

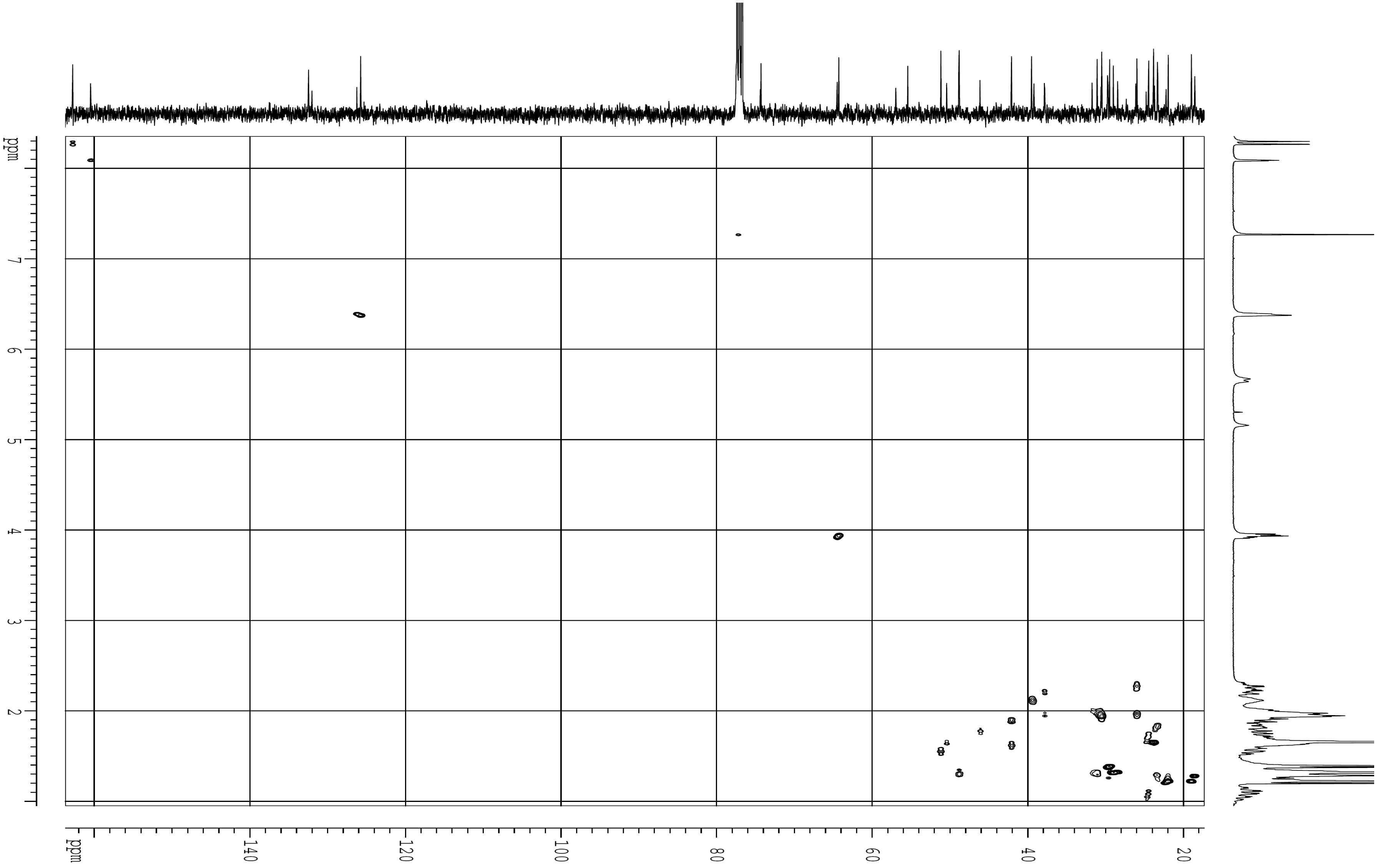

Figure S40. HMBC spectrum of kalihinene E (**5**) in $CDCl_3$.

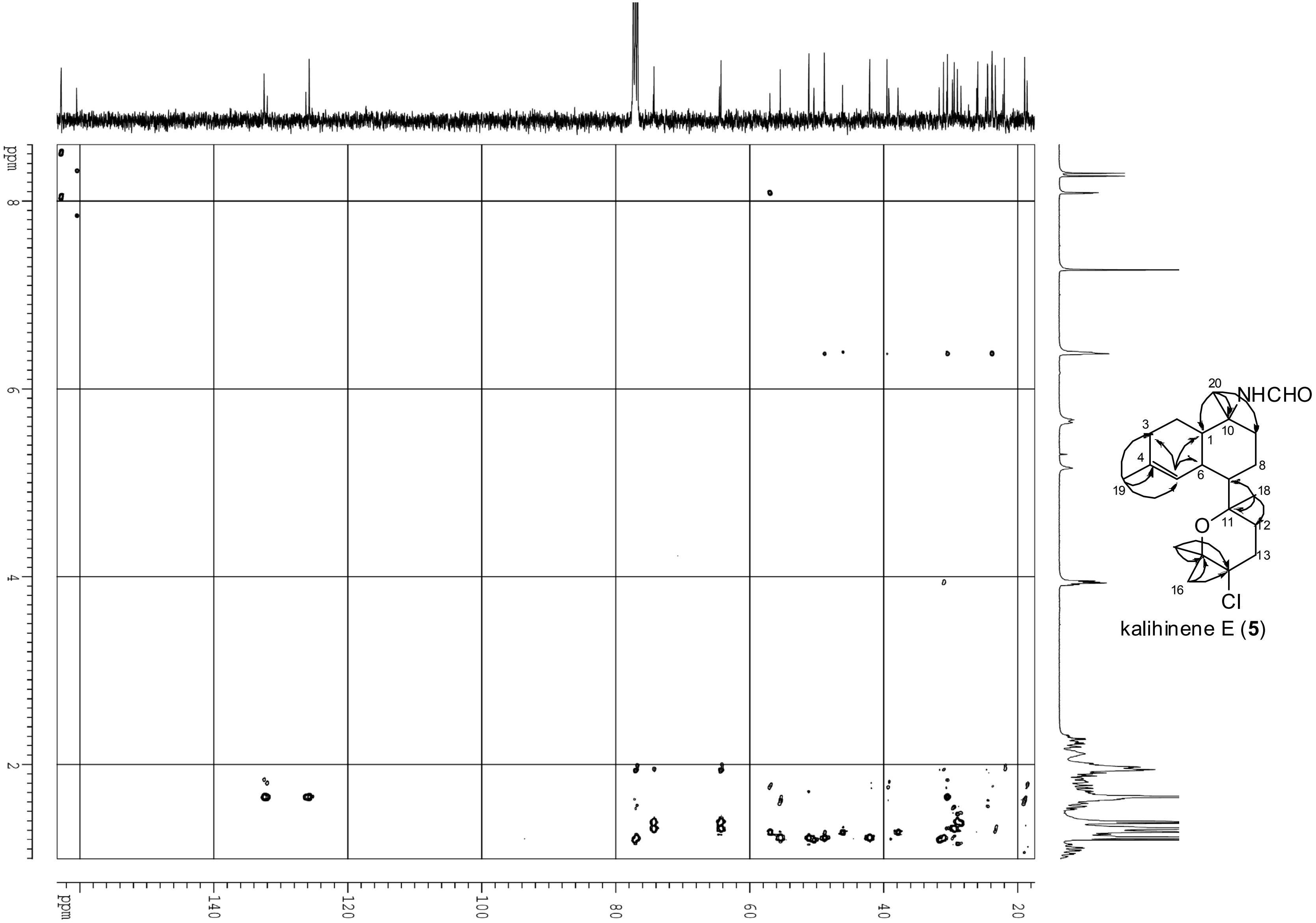

Figure S41. ^1H-^1H COSY spectrum of kalihinene E (**5**) in $CDCl_3$.

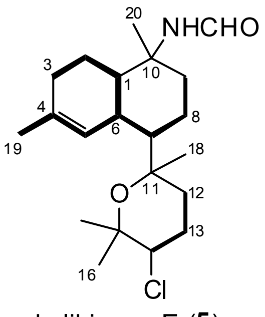

kalihinene E (**5**)

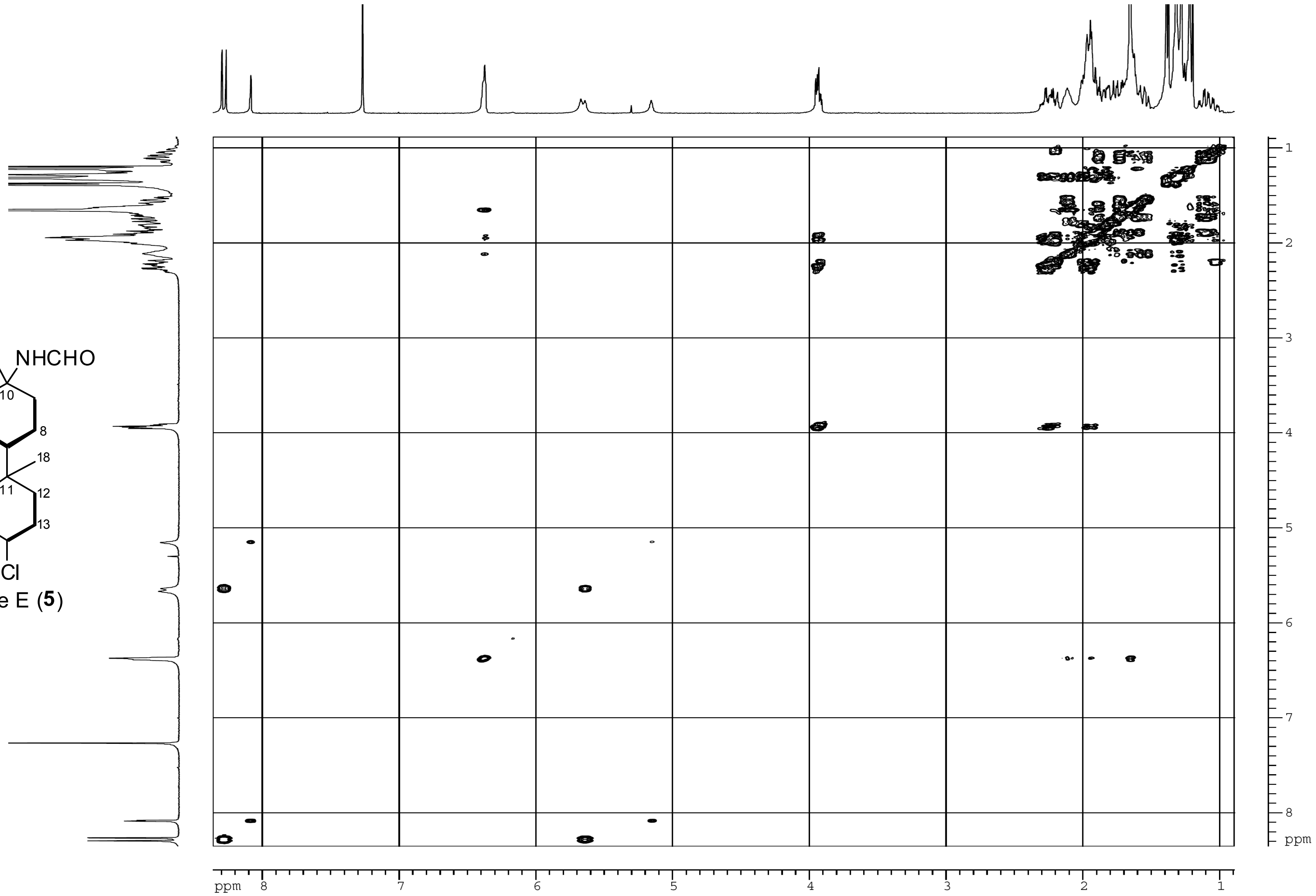

Figure S42. NOESY spectrum of kalihinene E (**5**) in $CDCl_3$.

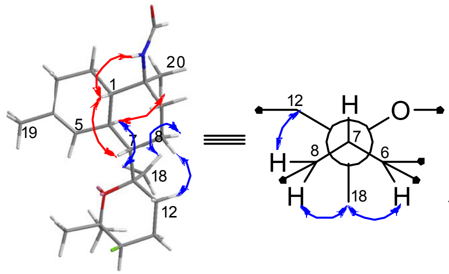

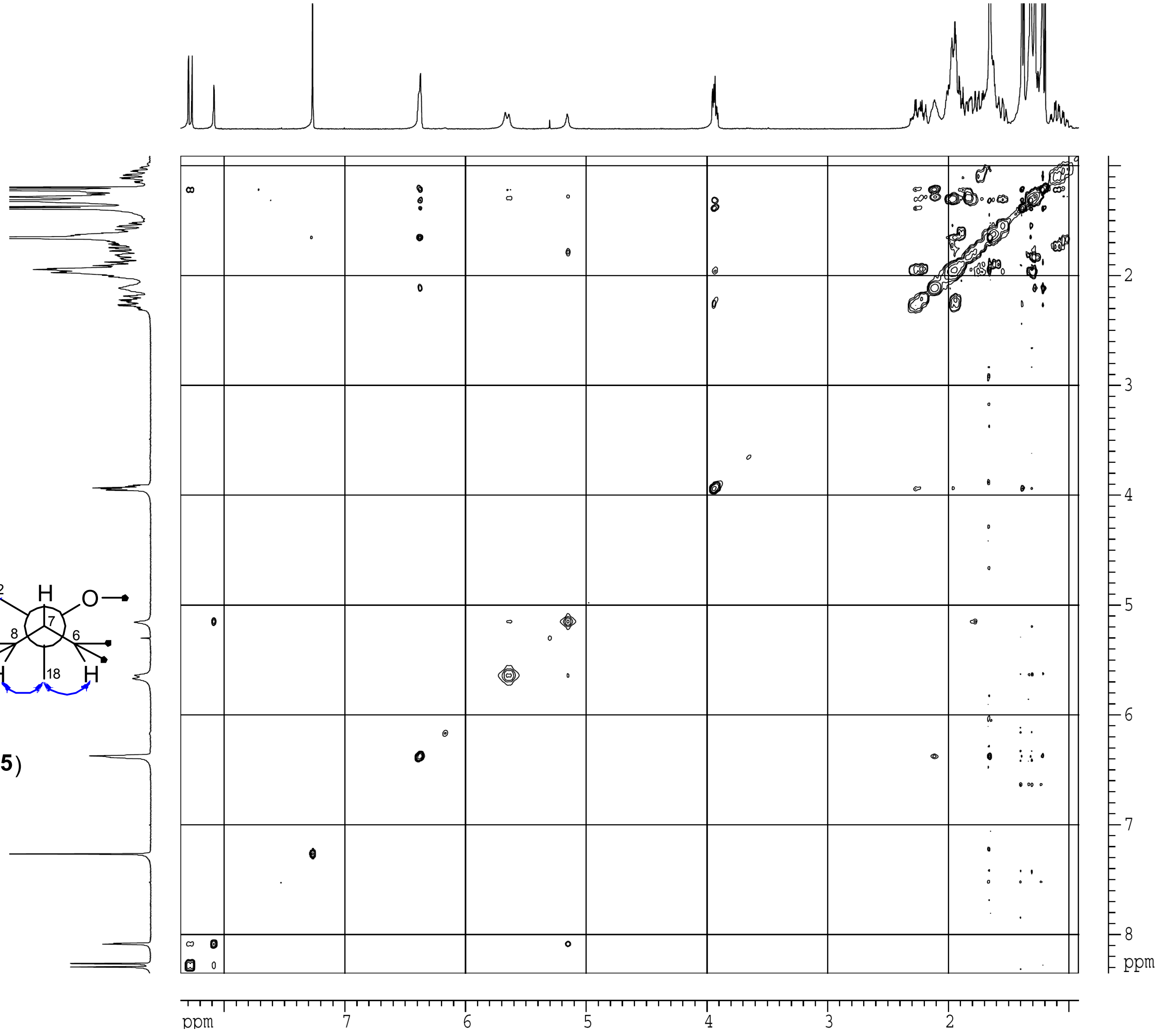

Figure S43. IR spectrum of kalihinene E (**5**).

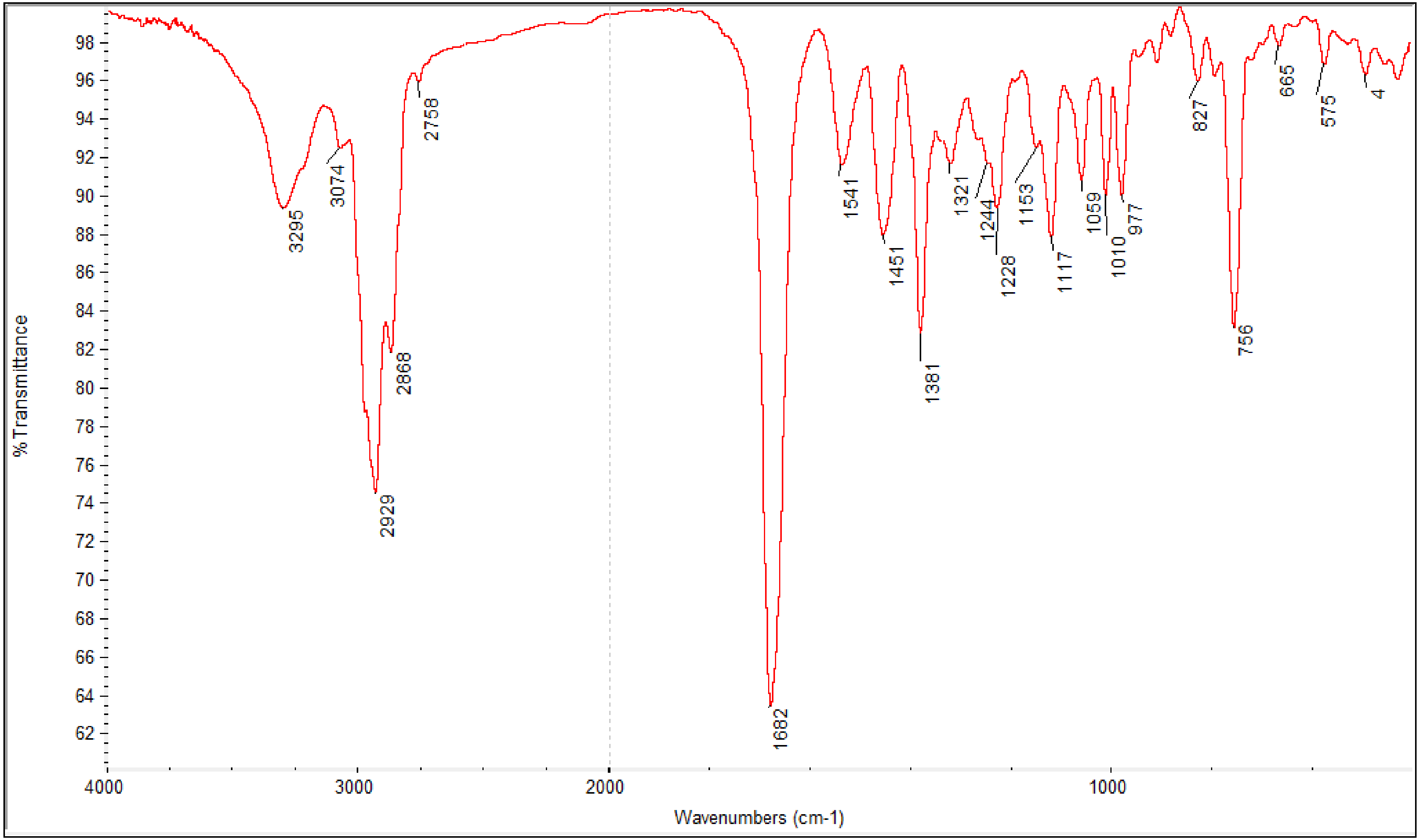

Figure S44. HRESIMS of kalihinene E (**5**).

Elemental Composition Report

Multiple Mass Analysis: 2 mass(es) processed
Tolerance = 10.0 PPM / DBE: min = -1.5, max = 50.0
Selected filters: None

Monoisotopic Mass, Even Electron Ions
53 formula(e) evaluated with 2 results within limits (up to 50 closest results for each mass)
Elements Used:
C: 10-23 H: 5-40 N: 1-1 O: 1-3 35Cl: 0-1 37Cl: 0-1

SIPI Q-Tof micro YA019 19-Mar-2011,14:51:28
0.00000000
WQ11-149H 50 (1.728) AM (Cen,6, 80.00, Ar,5000.0,354.24,0.70); Sm (SG, 2x3.00); Cm (48:65) TOF MS ES+
9.07e3

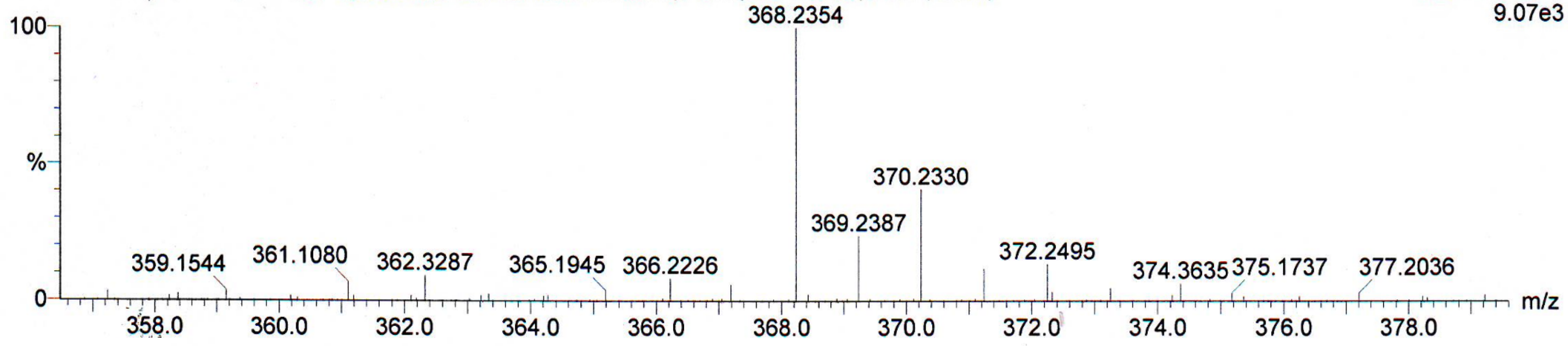

Minimum:	25.00				-1.5		
Maximum:	100.00		5.0	10.0	50.0		

Mass	RA	Calc. Mass	mDa	PPM	DBE	i-FIT	Formula
368.2354	100.00	368.2356	-0.2	-0.5	4.5	28.9	C21 H35 N O2 35Cl
370.2330	40.56	370.2327	0.3	0.8	4.5	502.6	C21 H35 N O2 37Cl

Figure S45. UV spectrum of kalihinene E (**5**).

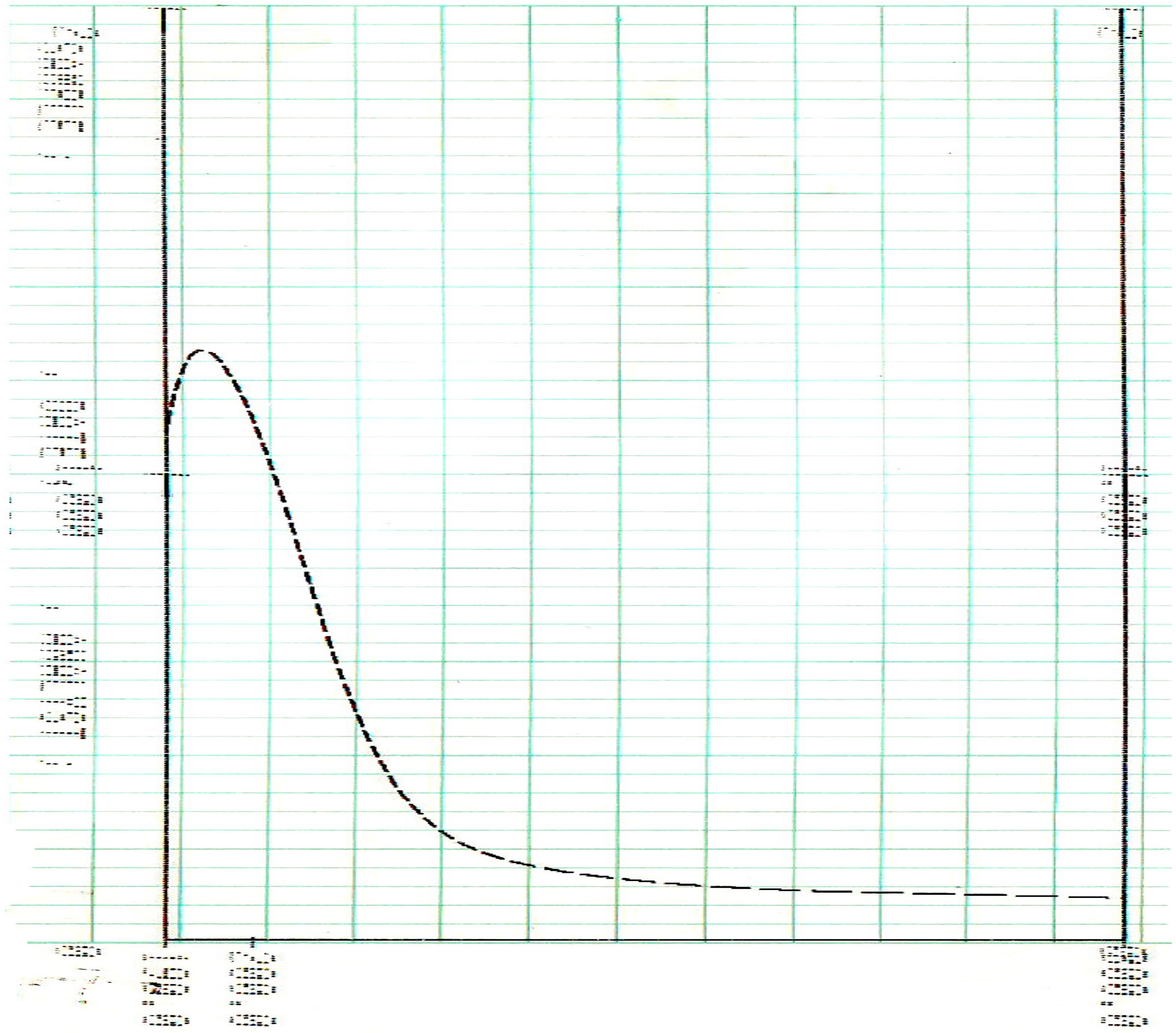

Figure S46. ^{1}H NMR spectrum of kalihinene F (**6**) in $CDCl_3$.

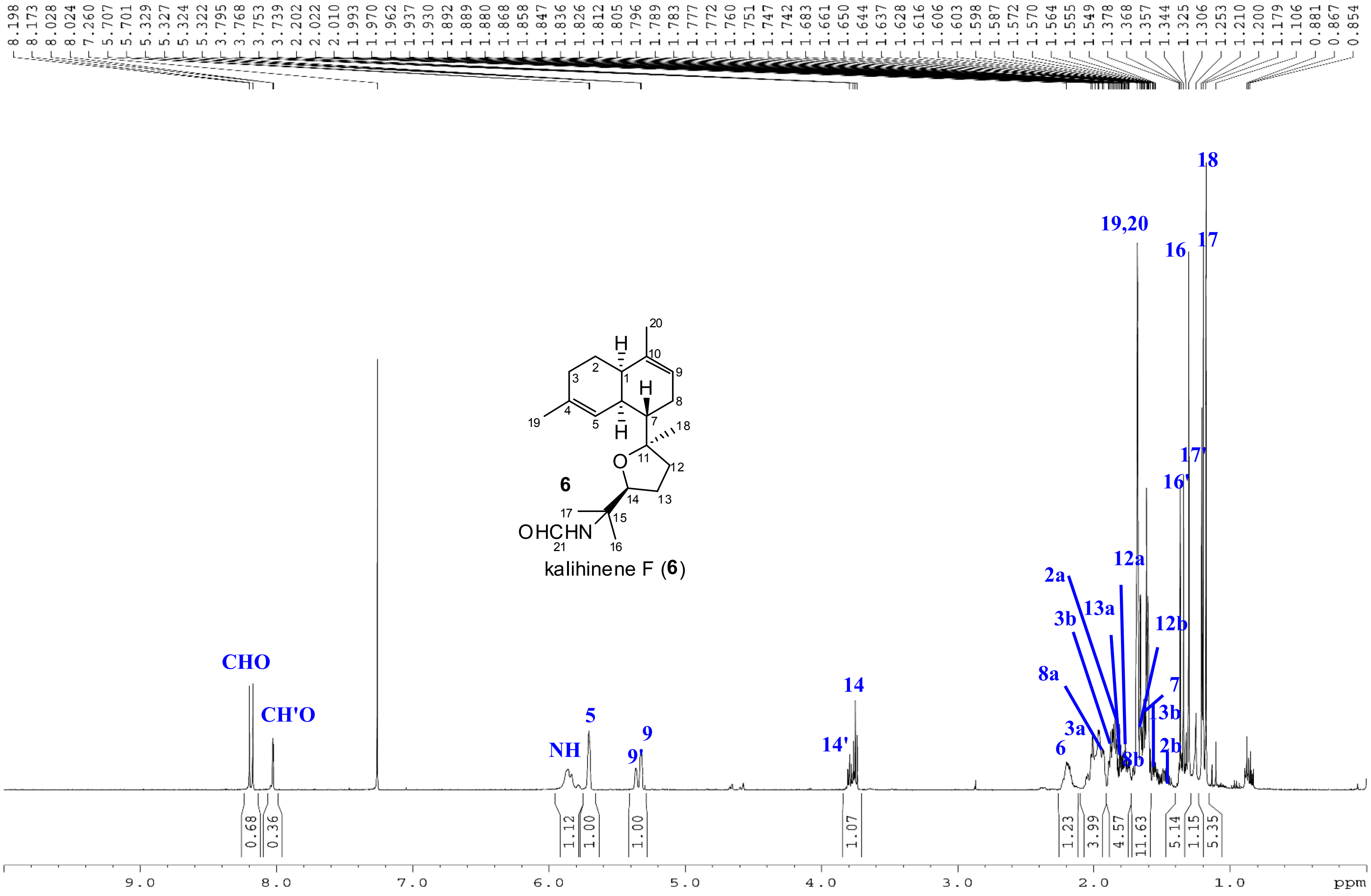

Figure S47. ^{13}C NMR spectrum of kalihinene F (**6**) in $CDCl_3$.

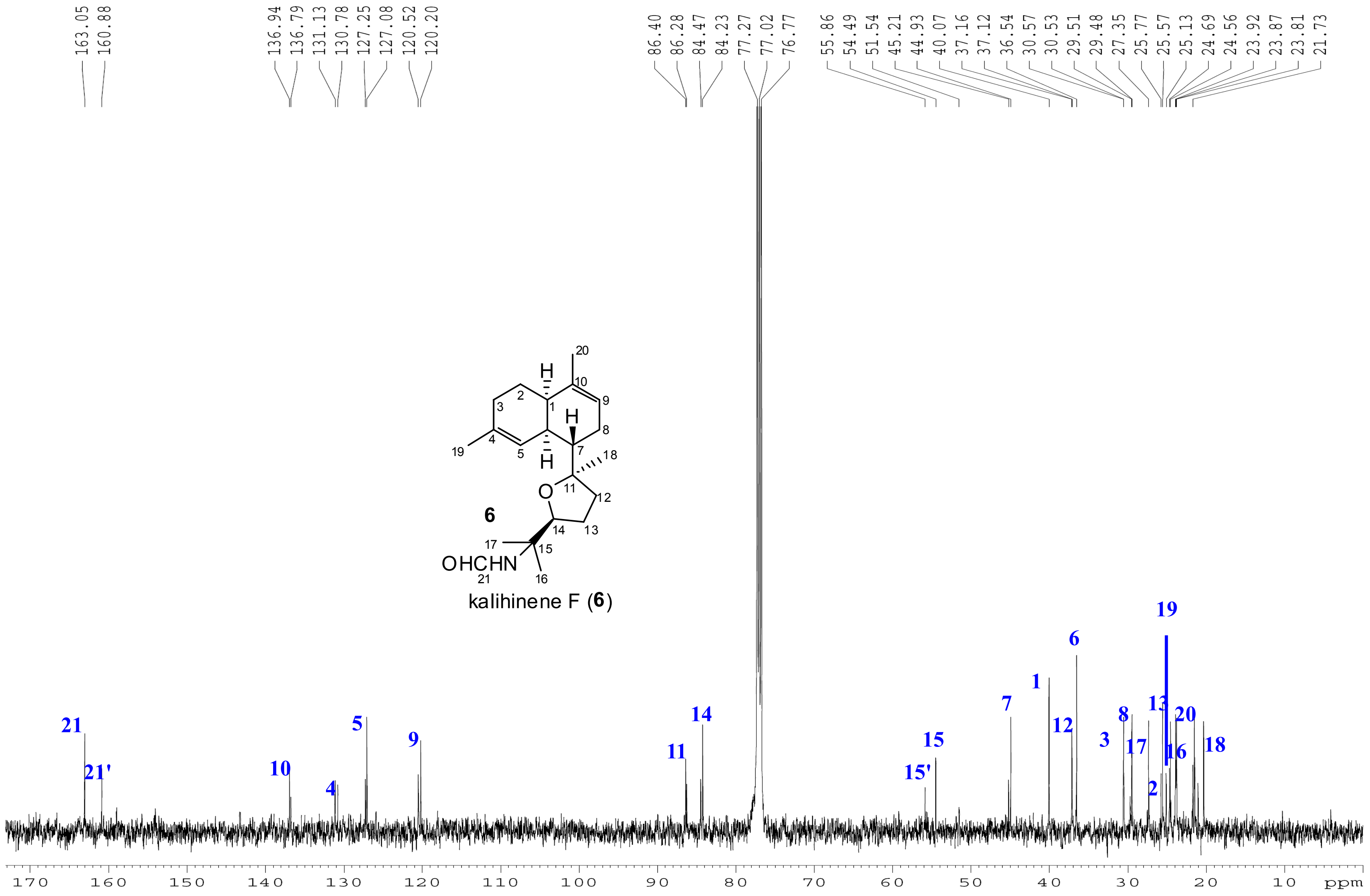

Figure S48. HSQC spectrum of kalihinene F(**6**) in $CDCl_3$.

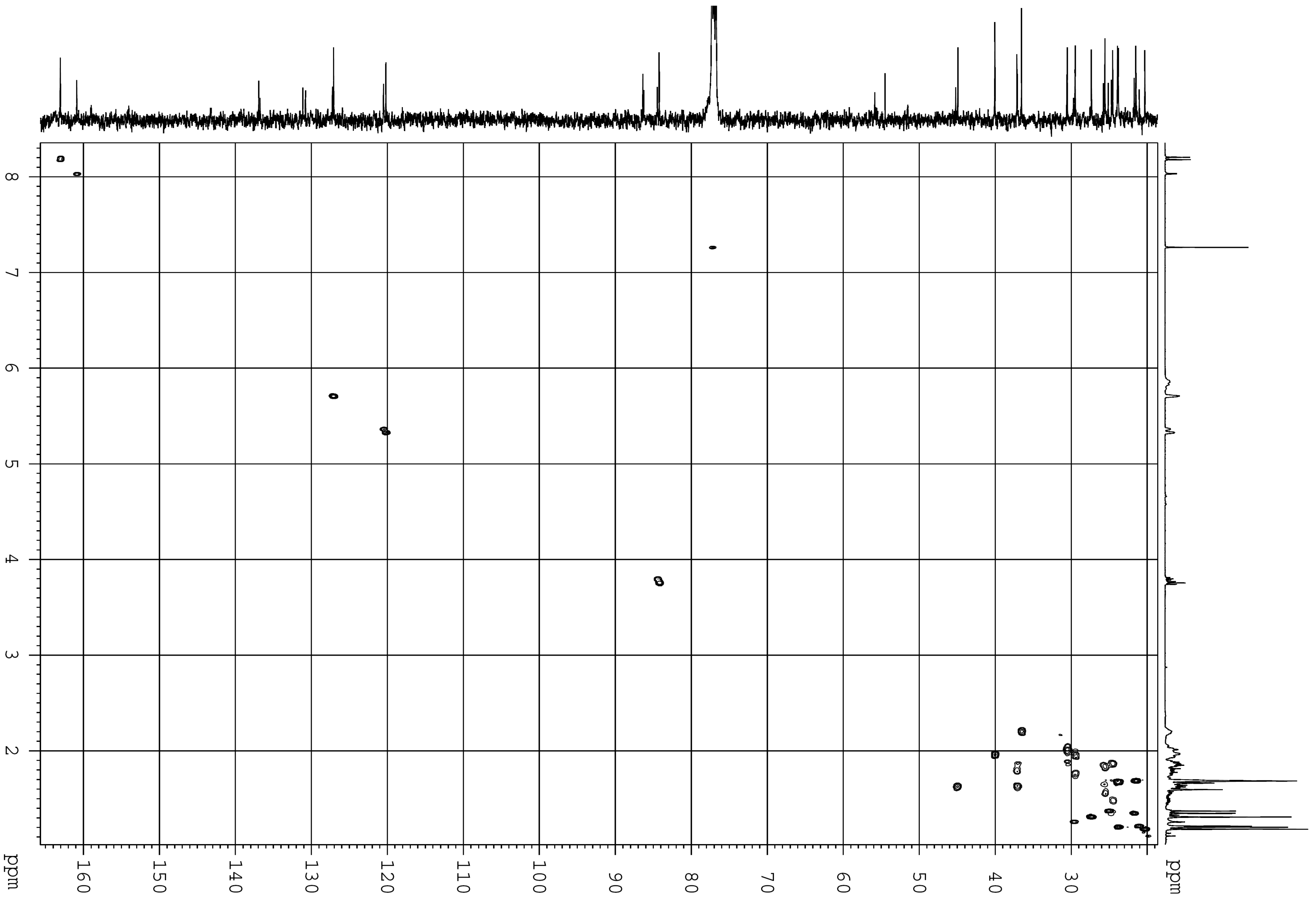

Figure S49. HMBC spectrum of kalihinene F (**6**) in $CDCl_3$.

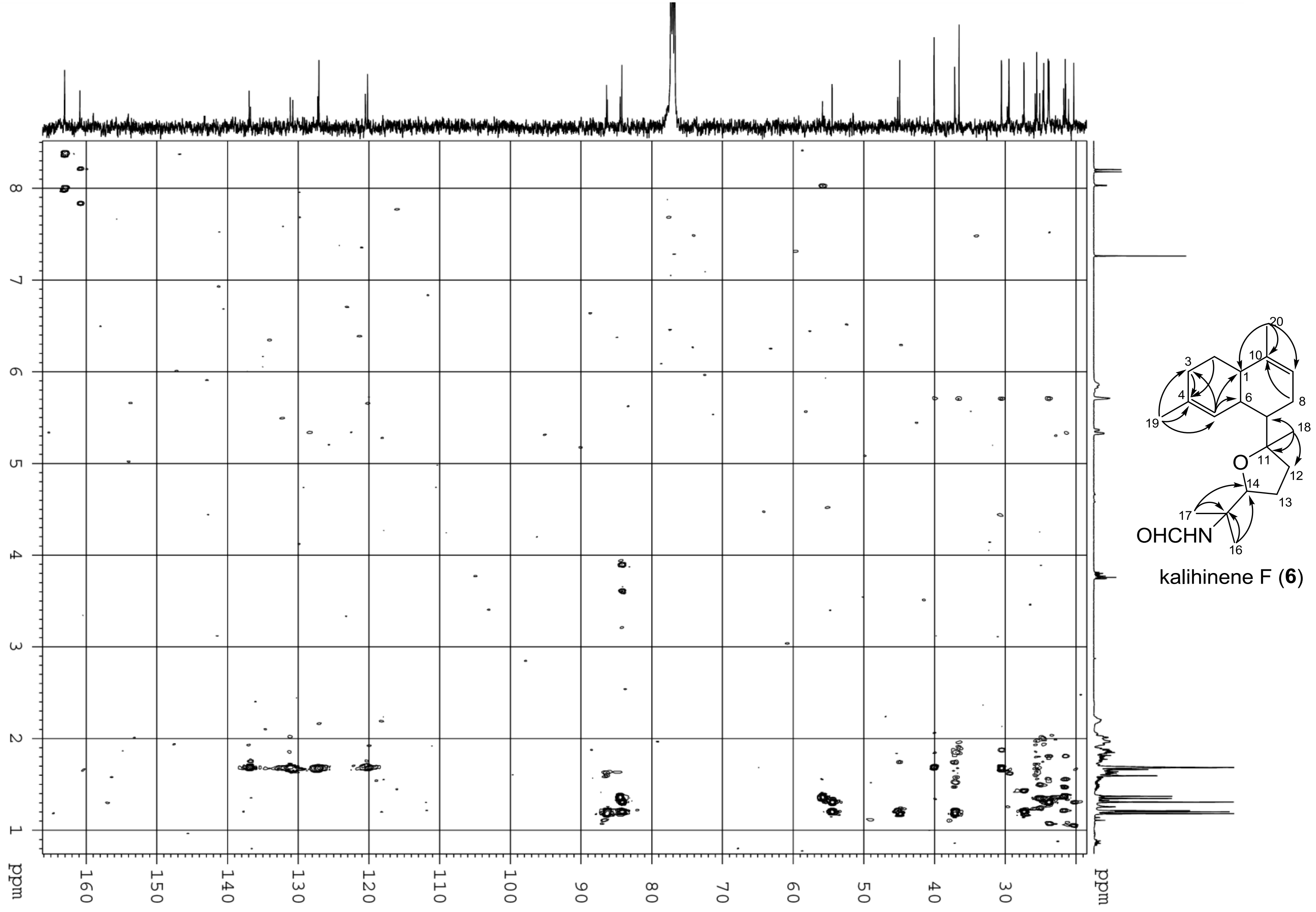

Figure S50. ^{1}H-^{1}H COSY spectrum of kalihinene F (**6**) in $CDCl_3$.

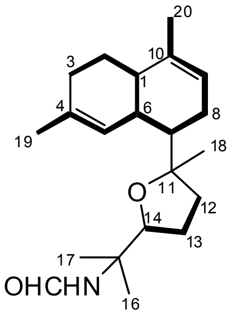

kalihinene F (**6**)

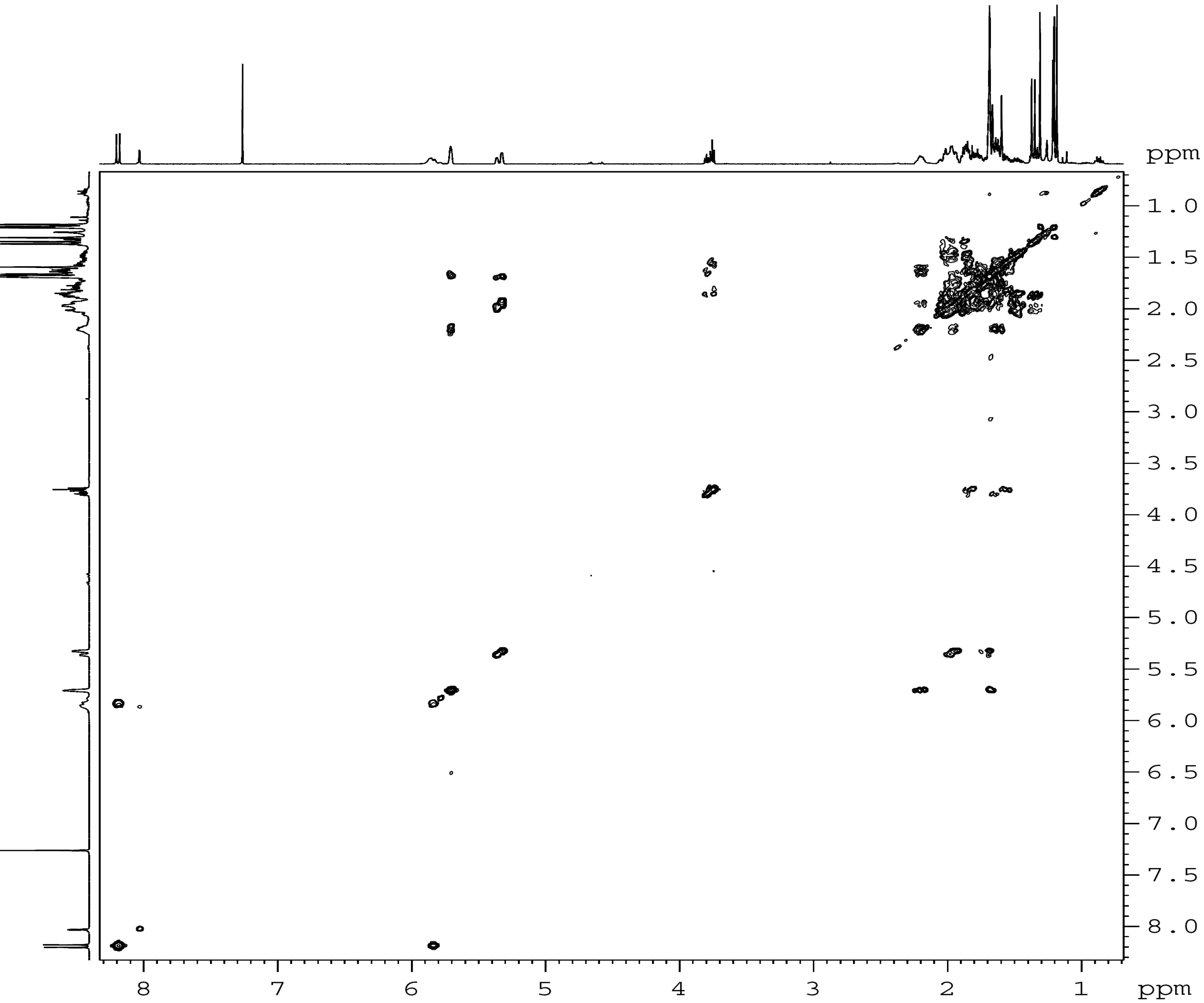

Figure S51. NOESY spectrum of kalihinene F (**6**) in $CDCl_3$.

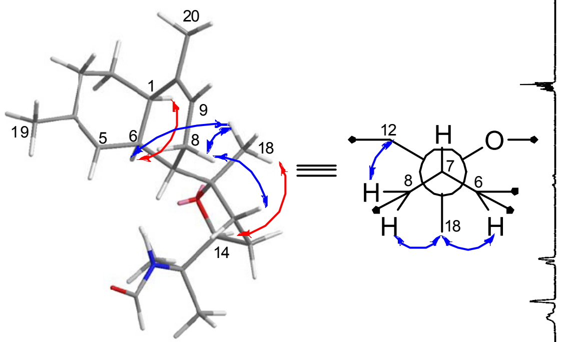

kalihinene F (**6**)

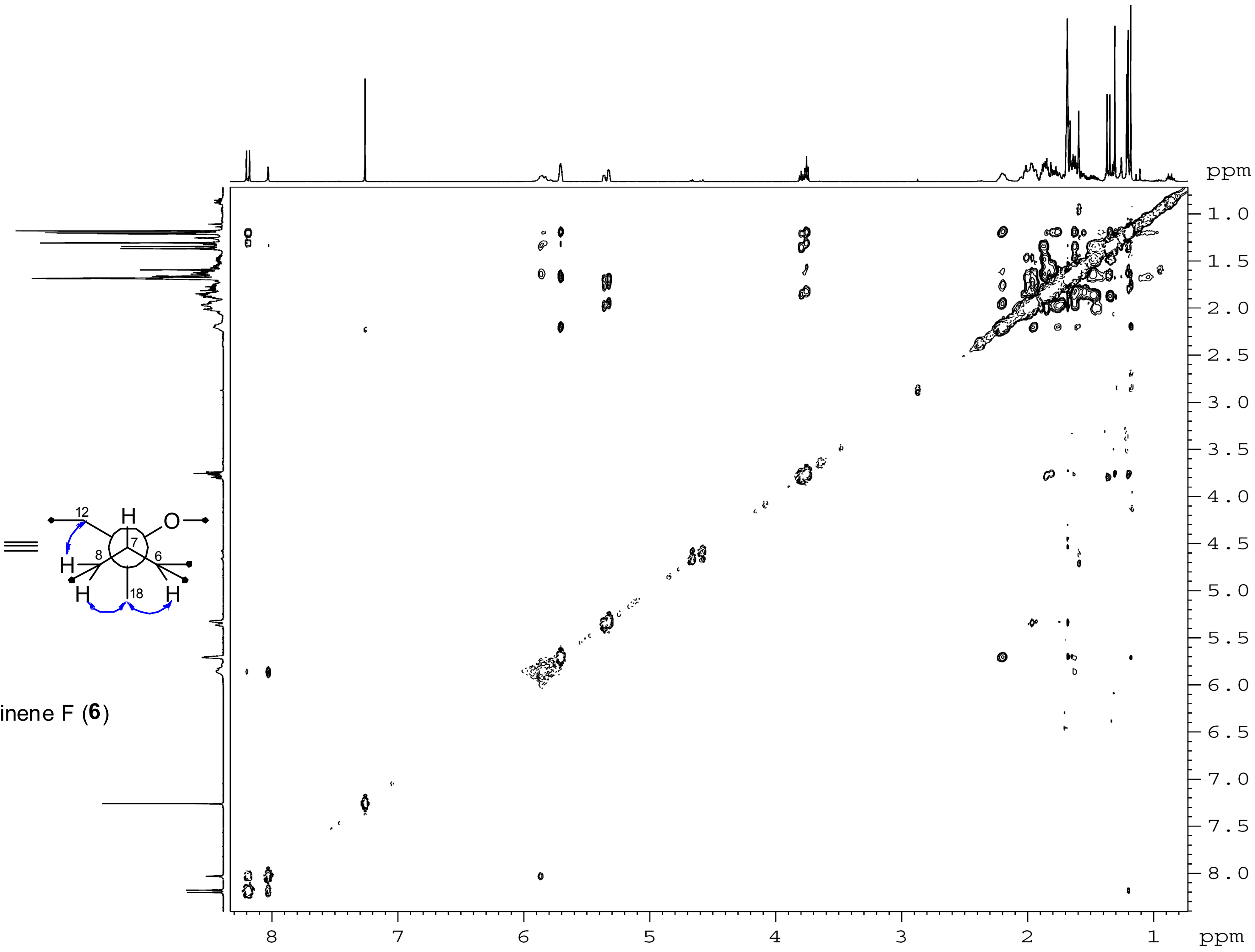

Figure S52. IR spectrum of kalihinene F (**6**).

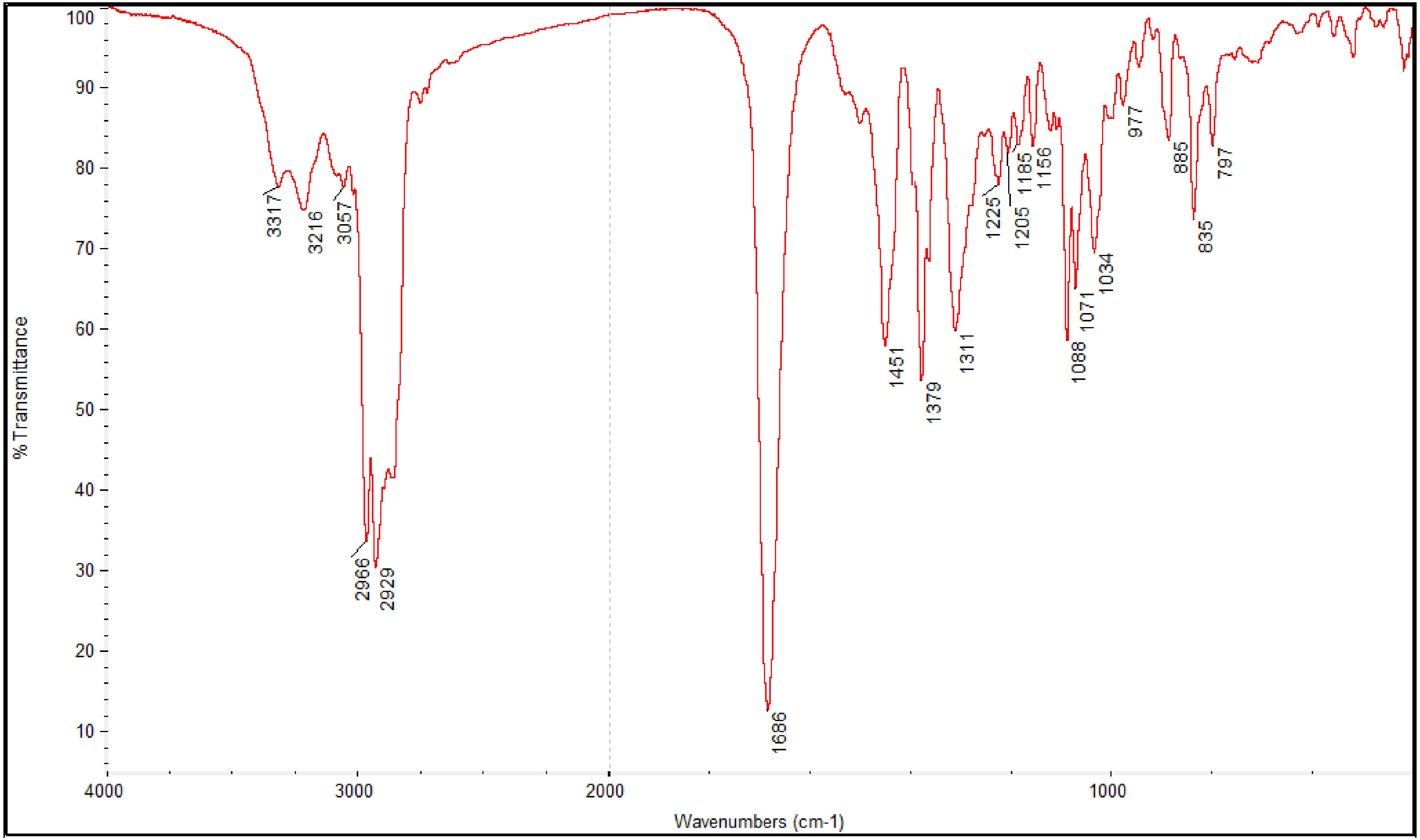

Figure S53. HRESIMS of kalihinene F (**6**).

Elemental Composition Report

Tolerance = 50.0 PPM / DBE: min = -1.5, max = 50.0
Selected filters: None

Monoisotopic Mass, Even Electron Ions
5 formula(e) evaluated with 1 results within limits (up to 50 closest results for each mass)
Elements Used:
C: 10-23 H: 5-40 N: 1-1 O: 1-2 Na: 1-1

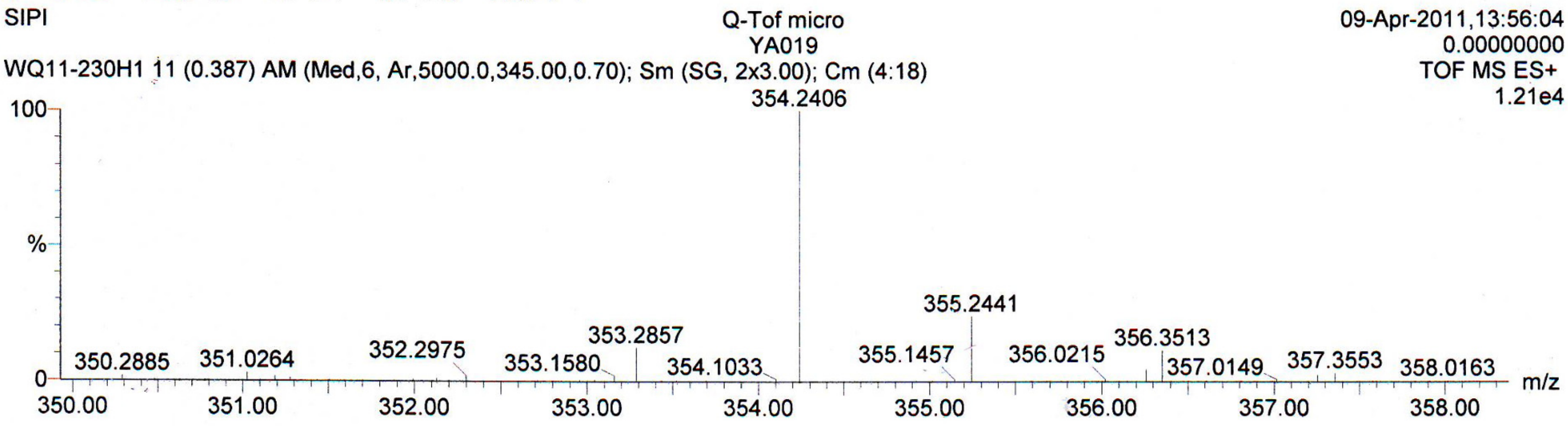

Minimum:	30.00				-1.5		
Maximum:	100.00		5.0	50.0	50.0		
Mass	RA	Calc. Mass	mDa	PPM	DBE	i-FIT	Formula
354.2406	100.00	354.2409	-0.3	-0.8	5.5	18.9	C21 H33 N O2 Na

Figure S54. UV spectrum of kalihinene F (**6**).

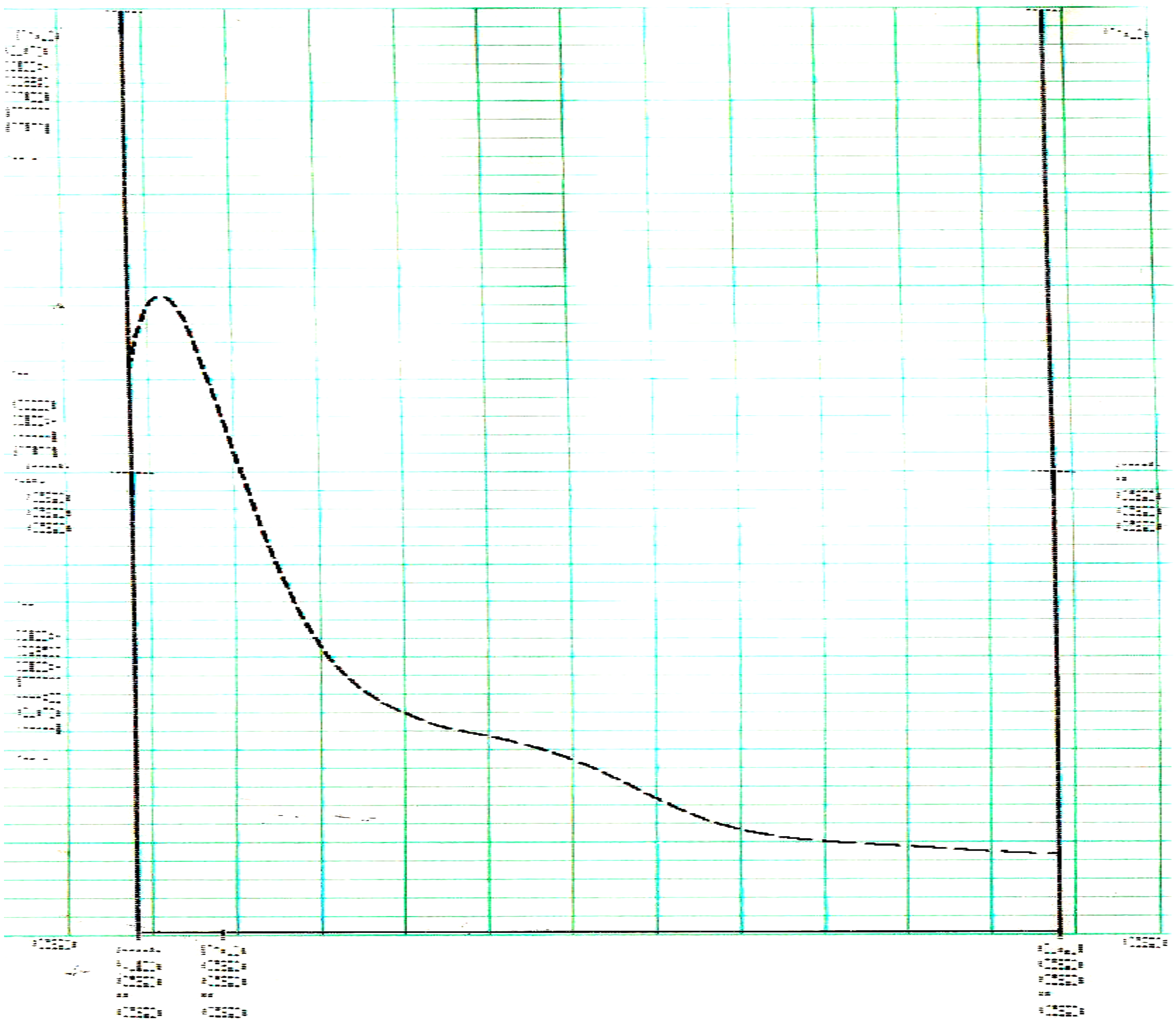

Figure S55. ^{1}H NMR spectrum of kalihipyran C (**7**) in $CDCl_3$.

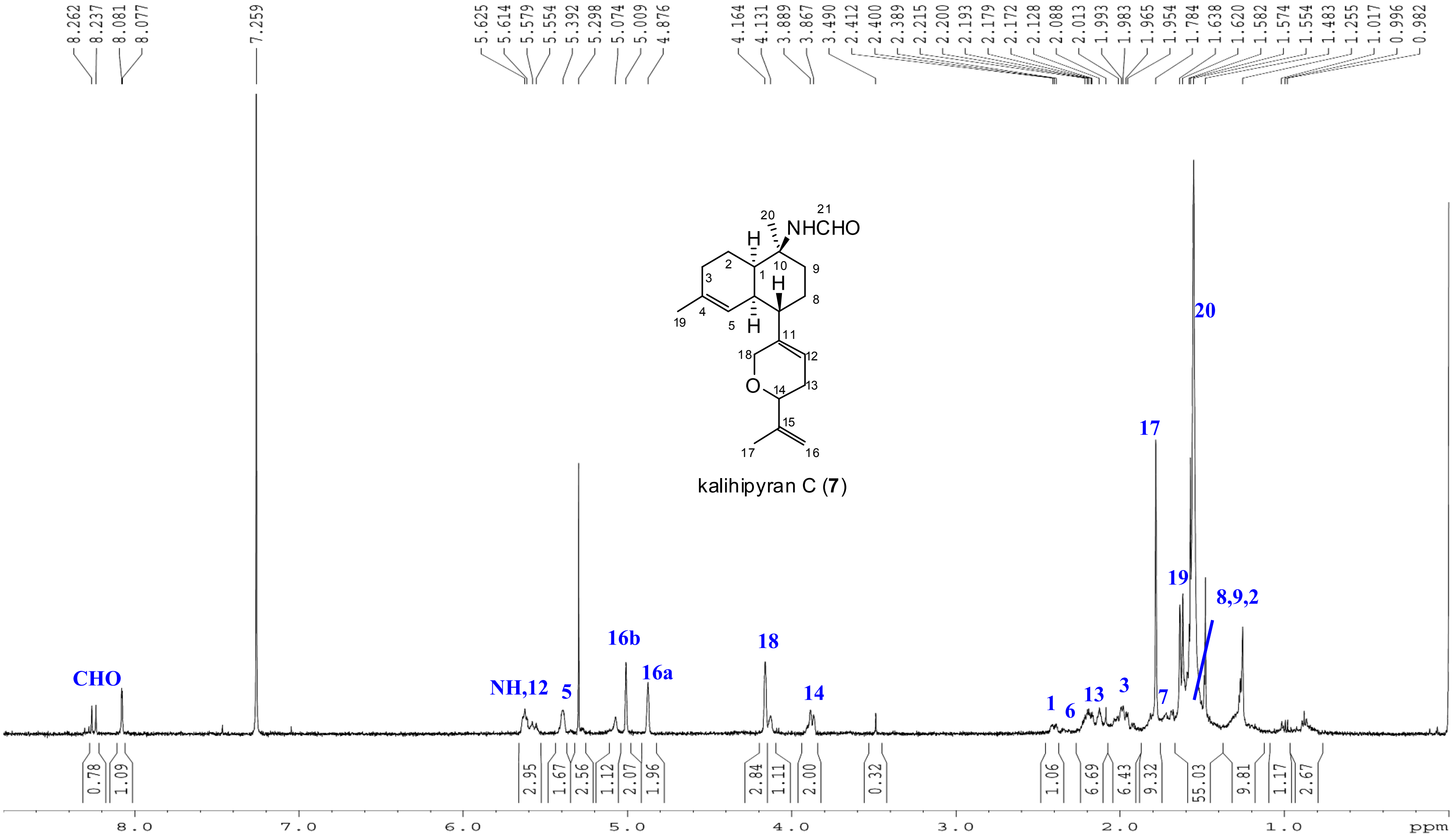

Figure S56. ^{13}C NMR spectrum of kalihipyran C (**7**) in $CDCl_3$.

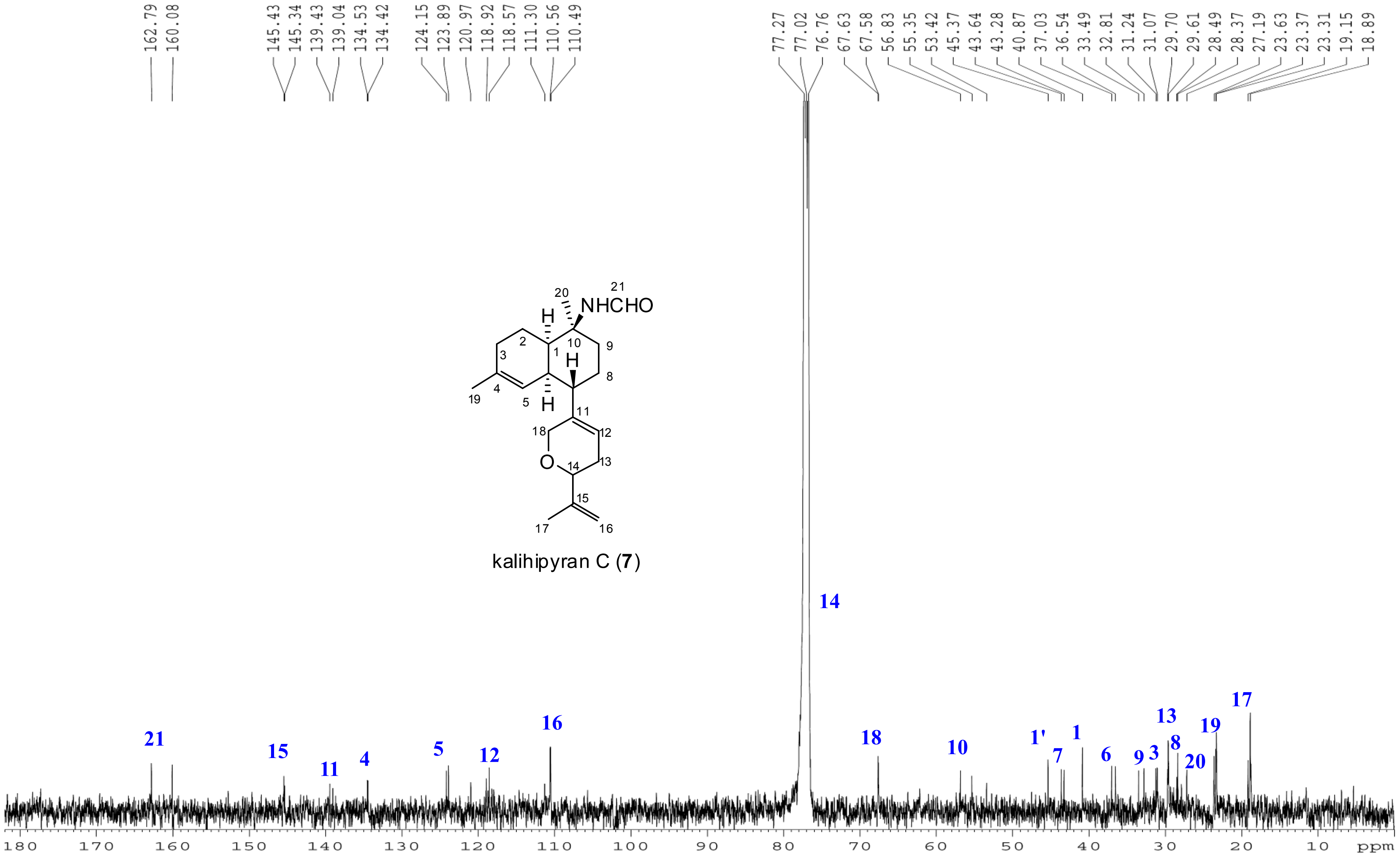

Figure S57. HSQC spectrum of kalihipyran C (**7**) in $CDCl_3$.

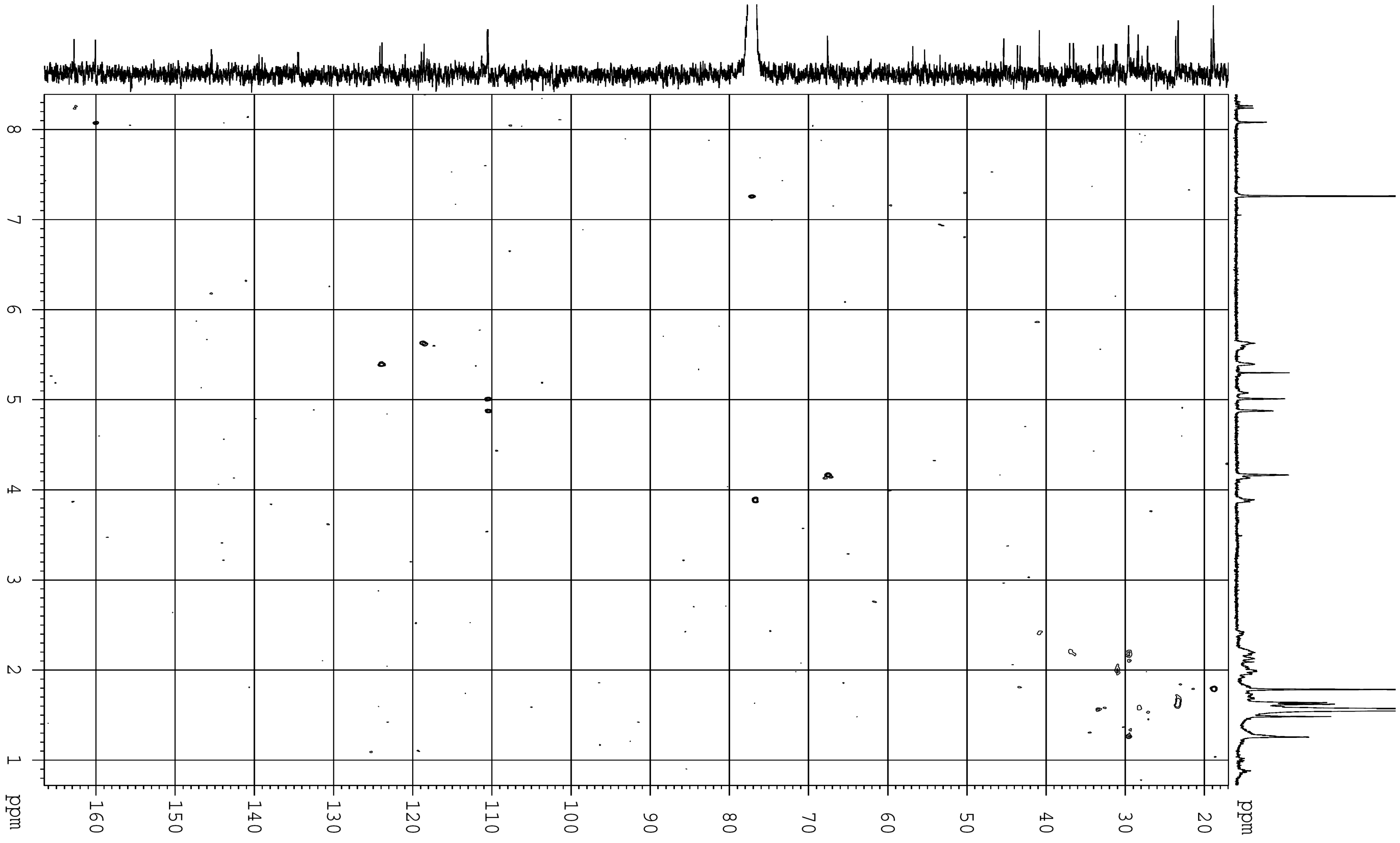

Figure S58. HMBC spectrum of kalihipyran C (**7**) in $CDCl_3$.

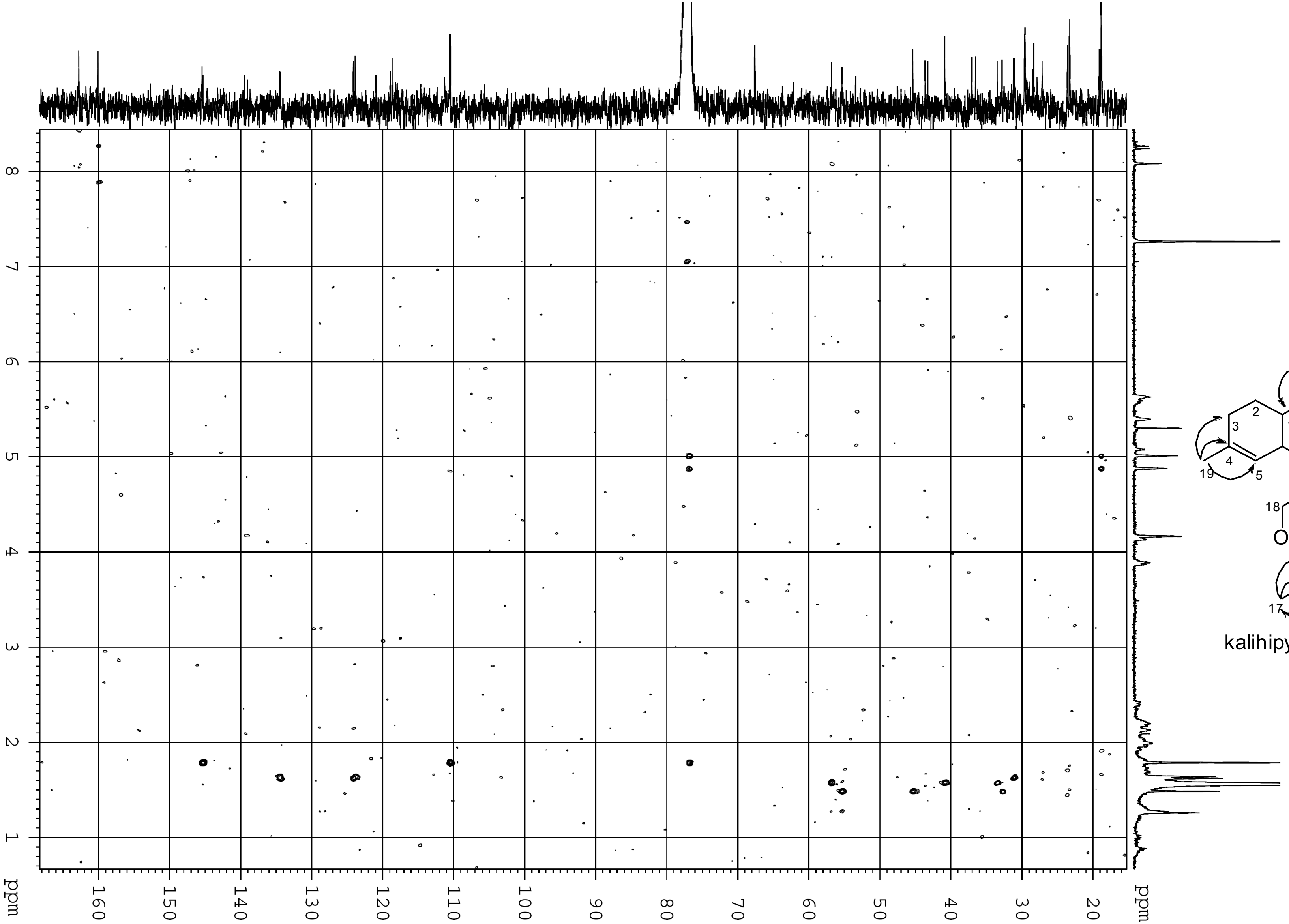

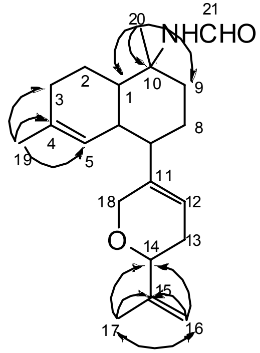

kalihipyran C (**7**)

Figure S59. ^{1}H-^{1}H COSY spectrum of kalihipyran C (**7**) in $CDCl_3$.

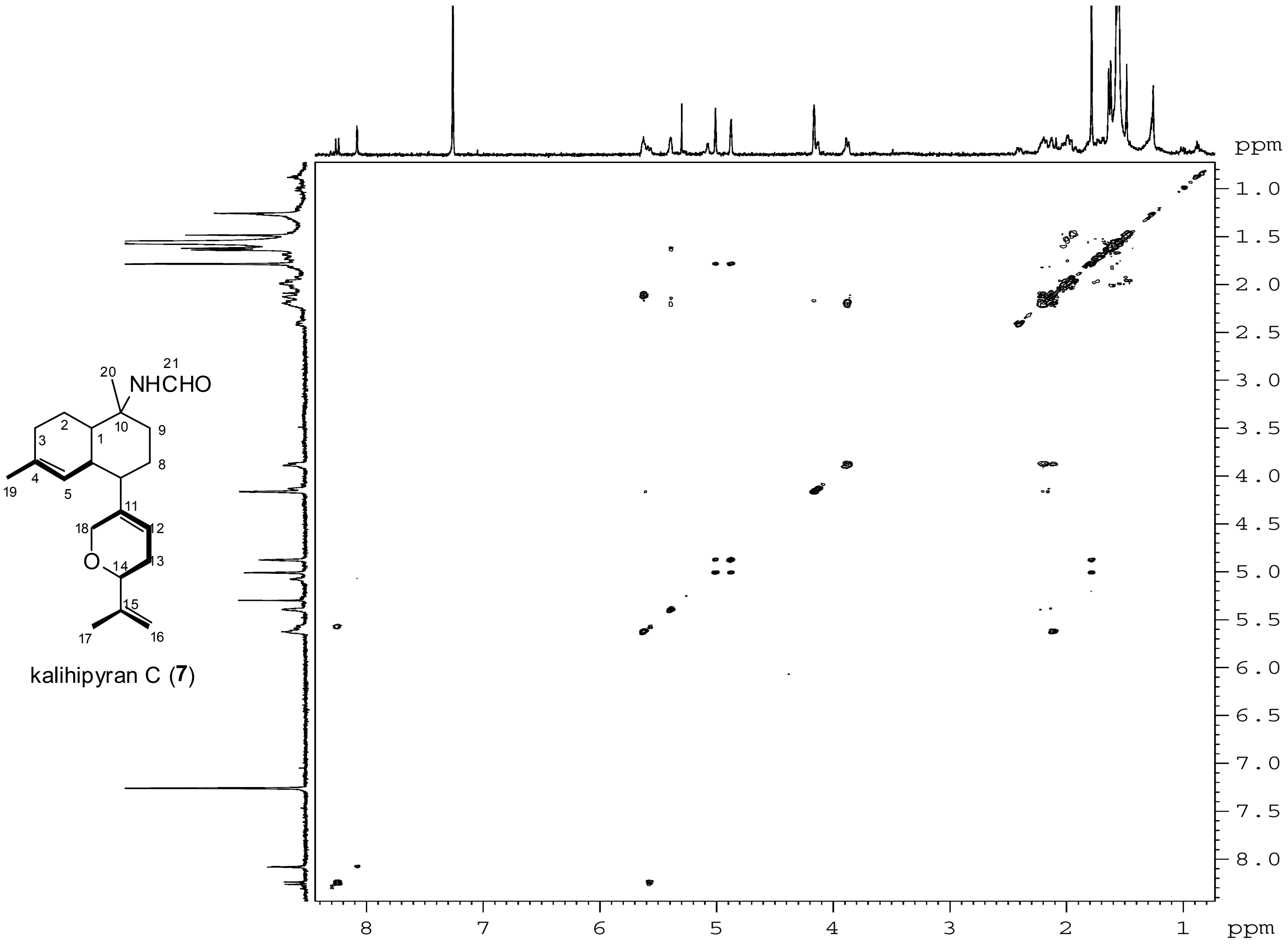

Figure S60. NOESY spectrum of kalihipyran C (**7**) in $CDCl_3$.

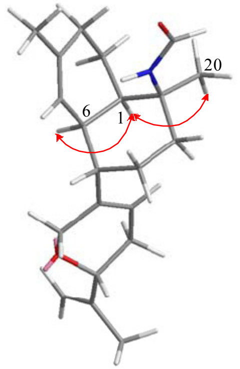

kalihipyran C (**7**)

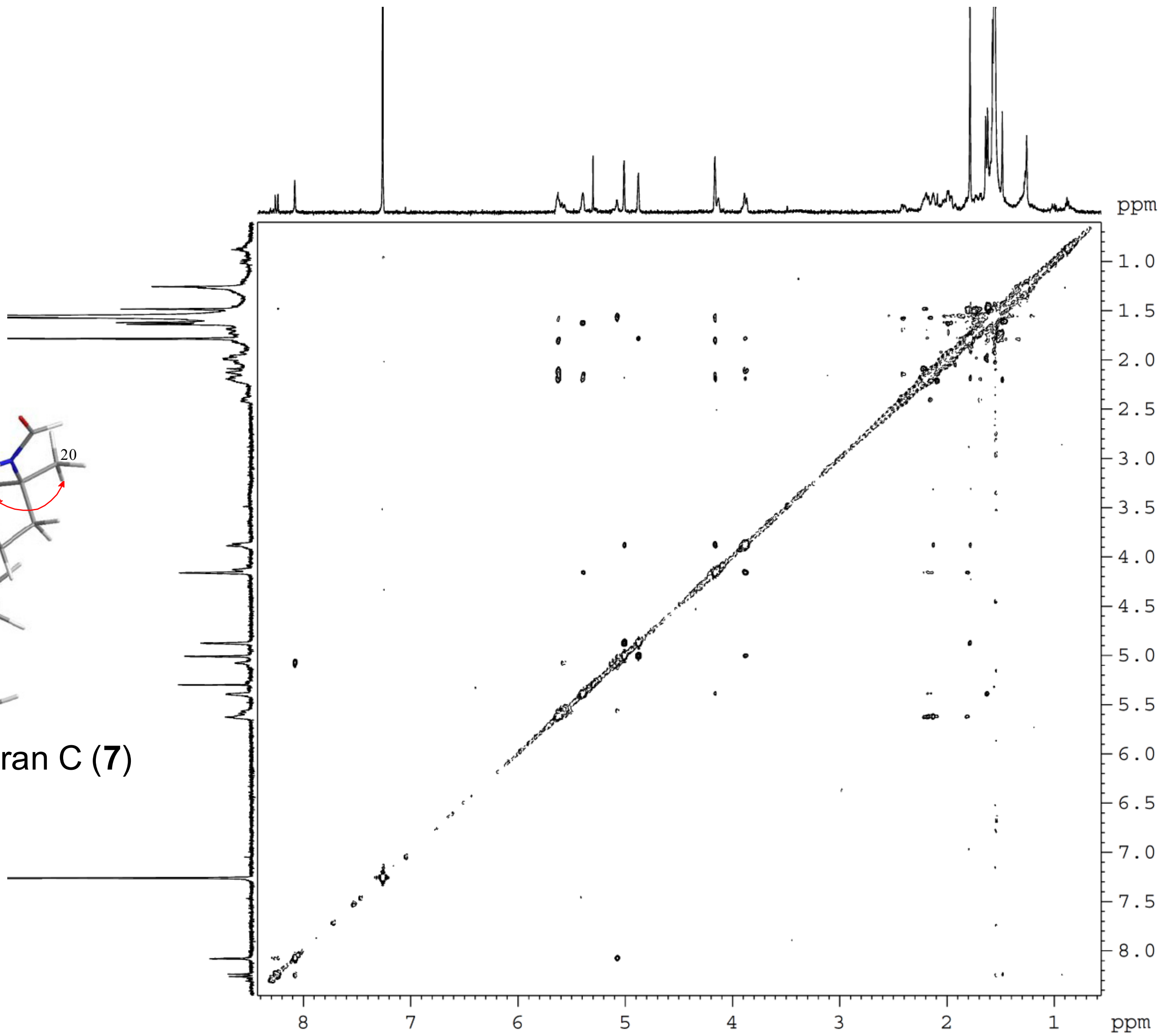

Figure S61. IR spectrum of kalihipyran C (**7**).

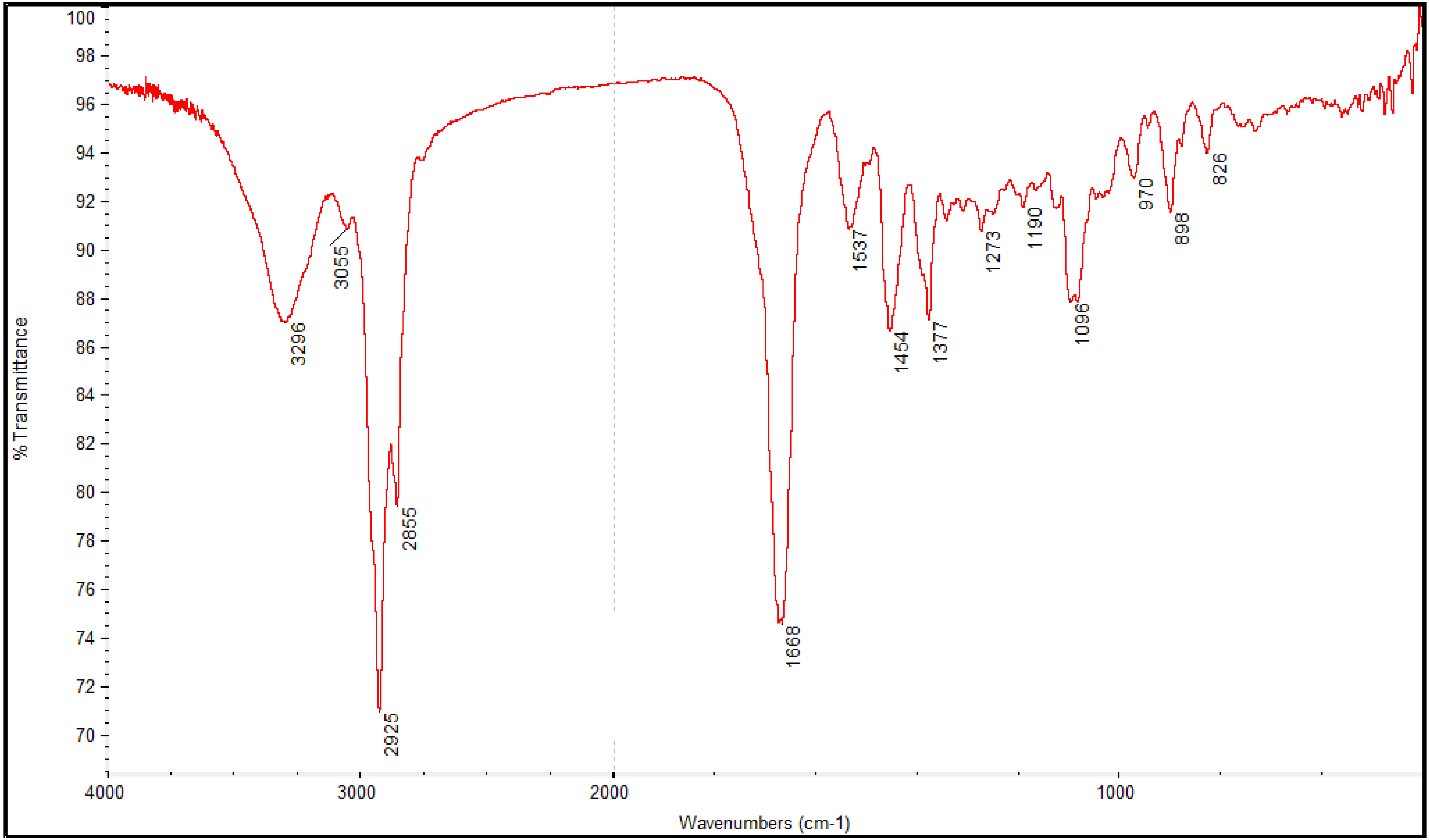

Figure S62. HRESIMS of kalihipyran C (**7**).

Elemental Composition Report

Tolerance = 10.0 PPM / DBE: min = -1.5, max = 50.0
Selected filters: None

Monoisotopic Mass, Even Electron Ions
5 formula(e) evaluated with 1 results within limits (up to 50 closest results for each mass)
Elements Used:
C: 5-25 H: 10-40 N: 1-1 O: 1-2 Na: 1-1

SIPI Q-Tof micro YA019 20-May-2011,13:23:30
0.00000000
WQ11-311H1 63 (2.218) AM (Cen,6, 80.00, Ar,5000.0,360.05,0.70); Sm (SG, 2x3.00); Cm (62:72) TOF MS ES+
1.22e4

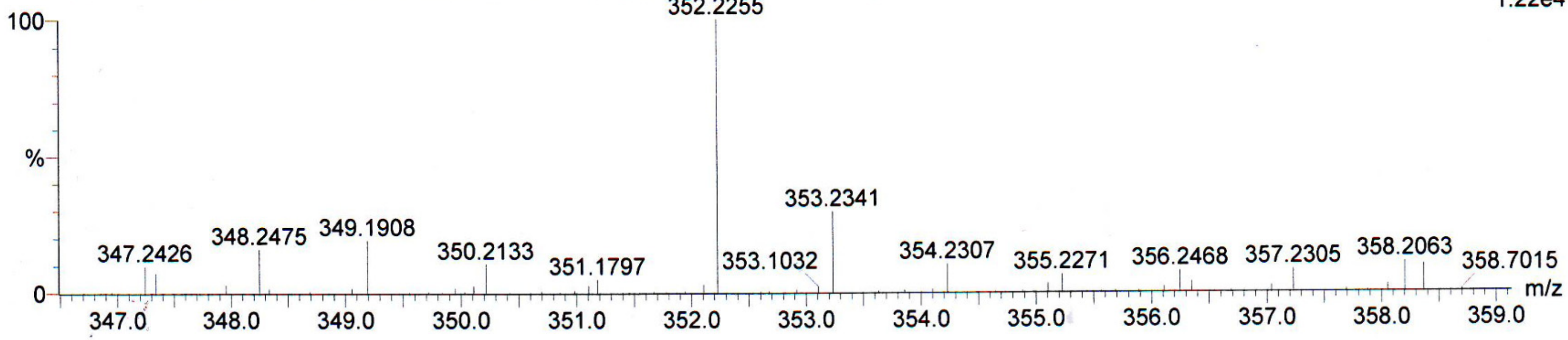

Minimum:	45.00				-1.5		
Maximum:	100.00		5.0	10.0	50.0		
Mass	RA	Calc. Mass	mDa	PPM	DBE	i-FIT	Formula
352.2255	100.00	352.2252	0.3	0.9	6.5	331.8	C21 H31 N O2 Na

Figure S63. UV spectrum of kalihipyran C (**7**).

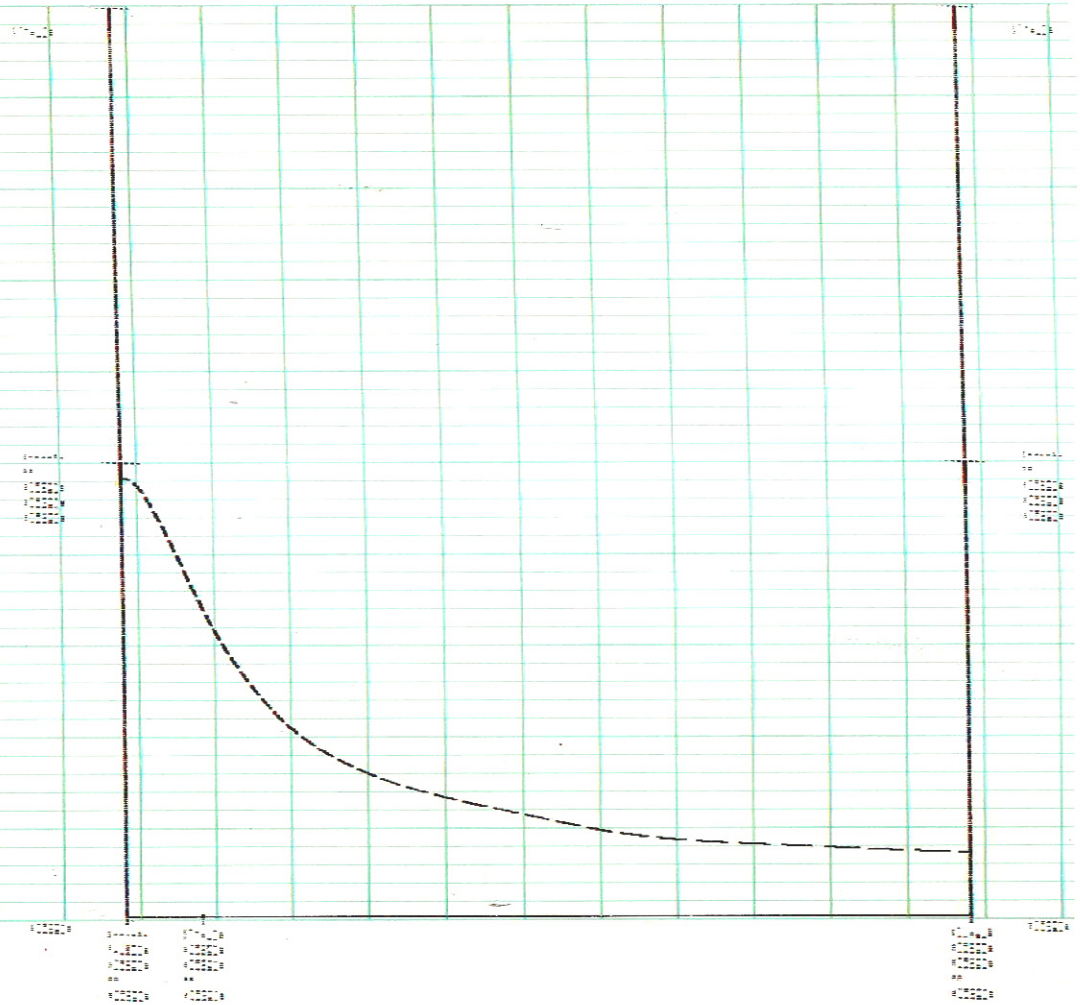

Supplement: Supplementary File 1: — PDF-Document (PDF, 9743 KB) [file marinedrugs-10-01445-s001.pdf]
